# Dominance of Cyclobutadienyl Over Cyclopentadienyl in the Crystal Field Splitting in Dysprosium Single‐Molecule Magnets

**DOI:** 10.1002/anie.202200525

**Published:** 2022-02-26

**Authors:** James P. Durrant, Benjamin M. Day, Jinkui Tang, Akseli Mansikkamäki, Richard A. Layfield

**Affiliations:** ^1^ Department of Chemistry University of Sussex Falmer Brighton BN1 9QR UK; ^2^ State Key Laboratory of Rare Earth Resource Utilization Changchun Institute of Applied Chemistry Chinese Academy of Sciences Changchun 130022 P.R. China; ^3^ NMR Research Unit University of Oulu P. O. Box 8000 90014 Oulu Finland

**Keywords:** Cyclobutadienyl Ligands, Dysprosium, Magnetic Anisotropy, Organometallic Compounds, Single-Molecule Magnets

## Abstract

Replacing a monoanionic cyclopentadienyl (Cp) ligand in dysprosium single‐molecule magnets (SMMs) with a dianionic cyclobutadienyl (Cb) ligand in the sandwich complexes [(η^4^‐Cb′′′′)Dy(η^5^‐C_5_Me_4_^*t*^Bu)(BH_4_)]^−^ (**1**), [(η^4^‐Cb′′′′)Dy(η^8^‐Pn^†^)K(THF)] (**2**) and [(η^4^‐Cb′′′′)Dy(η^8^‐Pn^†^)]^−^ (**3**) leads to larger energy barriers to magnetization reversal (Cb′′′′=C_4_(SiMe_3_)_4_, Pn^†^=1,4‐di(tri‐isopropylsilyl)pentalenyl). Short distances to the Cb′′′′ ligands and longer distances to the Cp ligands in **1**–**3** are consistent with the crystal field splitting being dominated by the former. Theoretical analysis shows that the magnetic axes in the ground Kramers doublets of **1**–**3** are oriented towards the Cb′′′′ ligands. The theoretical axiality parameter and the relative axiality parameter *Z* and *Z*_rel_ are introduced to facilitate comparisons of the SMM performance of **1**–**3** with a benchmark SMM. Increases in *Z* and *Z*_rel_ when Cb′′′ replaces Cp signposts a route to SMMs with properties that could surpass leading systems.

## Graphical abstract

Cyclobutadienyl ligands produce stronger crystal field splitting than cyclopentadienyl ligands in dysprosium organometallic sandwich complexes, leading to improved performance as single‐molecule magnets.

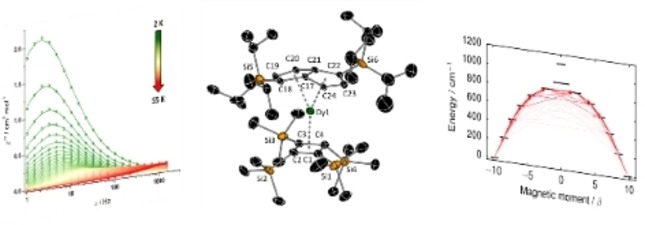

## Introduction

Molecular nanomagnets have potential to play an important role in the second quantum revolution.[^1^, ^2^, ^3^] For instance, observations of magnetic hysteresis in certain types of d‐ and f‐block coordination compound have inspired analogies between the properties of single‐molecule magnets (SMMs) and those of classical magnetic materials.[^4^, ^5^, ^6^, ^7^, ^8^, ^9^, ^10^] The analogy has led to suggestions that SMMs could be incorporated into devices capable of storing digital information, but with the advantage that the sub‐nanometer dimensions of magnetic molecules could permit greater storage densities than can be achieved with extended solids. It has also been shown that the hysteresis in SMMs can be retained in single molecules on surfaces, an important step towards the development of devices.[^11^, ^12^] Furthermore, studies of single‐molecule and single‐atom magnets has unearthed an abundance of rich physics with potential to drive the discovery of quantum technologies.[^13^, ^14^, ^15^, ^16^, ^17^]

Amongst the obstacles to the fabrication of devices containing SMMs is the fact that their performance diminishes with increasing temperature. Strategies for addressing this challenge focus primarily on crystal field engineering, aiming to increase the effective energy barrier (*U*_eff_) to reversal of the magnetization and the magnetic blocking temperature (*T*_B_). Large *U*_eff_ values occur in dysprosium compounds where the ^6^H_15/2_ ground multiplet of Dy^3+^ experiences a strong, highly axial crystal field.[^18^, ^19^, ^20^] In such systems, if the equatorial crystal field is negligible, mixing between the low‐lying *M_J_* states in the ground multiplet is weak and magnetic hysteresis occurs with remanence and coercivity, often equating to a high blocking temperature.

Some of the most striking SMM properties have been identified in dysprosium metallocene cations of the type [(η^5^‐Cp^R^)_2_Dy]^+^ (Cp^R^ = a substituted cyclopentadienyl ligand),[^21^, ^22^, ^23^, ^24^, ^25^, ^26^, ^27^] the molecular structures of which come closest to fulfilling the criteria for high‐temperature performance.[^28^, ^29^, ^30^] The energy barriers and blocking temperatures in these cations can be interpreted in terms of the size of the substituents on the monoanionic [Cp^R^]^−^ ligands, a key finding being that while steric bulk promotes axiality it may also weaken the crystal field. Beyond this simplistic analysis, theoretical studies have revealed that the vibrational modes within the ligand play an important role in magnetic relaxation.[^23^, ^31^]

In light of the observations on dysprosocenium SMMs, it has been suggested that their performance may already have been optimized.^[32]^ Hence, there is a need to go beyond the metallocene paradigm by developing new ligand environments. The benchmark performance for an SMM is currently represented by the energy barrier of 1541 cm^−1^ and blocking temperature of 80 K reported for [(Cp^*i*Pr5^)Dy(Cp*)]^+^ (the 5* cation, Cp*=C_5_Me_5_).^[31]^ Based on a qualitative magneto‐structural correlation, replacing a cyclopentadienyl ligand in a metallocene SMM with a cyclobutadienyl ligand of the type [η^4^‐C_4_R_4_]^2−^ (Cb^R^, R=bulky substituent) should lead to a stronger crystal field. Provided the resulting dysprosium sandwich complexes retain axial geometries, their SMM properties should outperform the analogous cyclopentadienyl‐only compounds.

## Results and Discussion

**Scheme 1:**
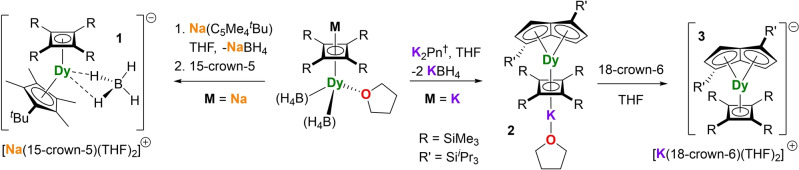
Synthesis of [Na(15‐crown‐5)(THF)_2_][(η^4^‐Cb′′′′)Dy(η^5^‐C_5_Me_4_^*t*^Bu)(BH_4_)] (**1**), [(η^4^‐Cb′′′′)Dy(η^8^‐Pn^†^)K(THF)] (**2**) and [K(18‐c‐6)(THF)_2_][(η^4^‐Cb′′′′)Dy(η^8^‐Pn^†^)] (**3**).

To test the cyclobutadienyl hypothesis, we now report our findings on the dysprosium SMMs [Na(15‐crown‐5)(THF)_2_][(η^4^‐Cb′′′′)Dy(η^5^‐C_5_Me_4_^*t*^Bu)(BH_4_)], [(η^4^‐Cb′′′′)Dy(η^8^‐Pn^†^)K(THF)] and [K(18‐crown‐6)(THF)_2_][(η^4^‐Cb′′′′)Dy(η^8^‐Pn^†^)], where Cb′′′′ is tetra(trimethylsilyl)cyclobutadienyl and Pn^†^ is 1,4‐di(tri‐isopropylsilyl)pentalenyl (Scheme 1).^[33]^ The reaction between the half‐sandwich compound [Na(η^4^‐Cb′′′′)Dy(BH_4_)_2_(THF)]^[34]^ and [Na(C_5_Me_4_^*t*^Bu)], followed by the addition of one equivalent of 15‐crown‐5, resulted in formation of [(η^4^‐Cb′′′′)Dy(η^5^‐C_5_Me_4_^*t*^Bu)(BH_4_)]^−^ (**1**) as the salt of [Na(15‐crown‐5)(THF)_2_]^+^. A similar reaction of [K(η^4^‐Cb′′′′)Dy(BH_4_)_2_(THF)] with [K_2_Pn^†^] yielded the contact ion pair [(η^4^‐Cb′′′′)Dy(η^8^‐Pn^†^)K(THF)] (**2**), which can be isolated and subsequently reacted with 18‐crown‐6 to give [(η^4^‐Cb′′′′)Dy(η^8^‐Pn^†^)]^−^ (**3**) as the salt of [K(18‐crown‐6)(THF)_2_]^+^.

**Figure 1:**
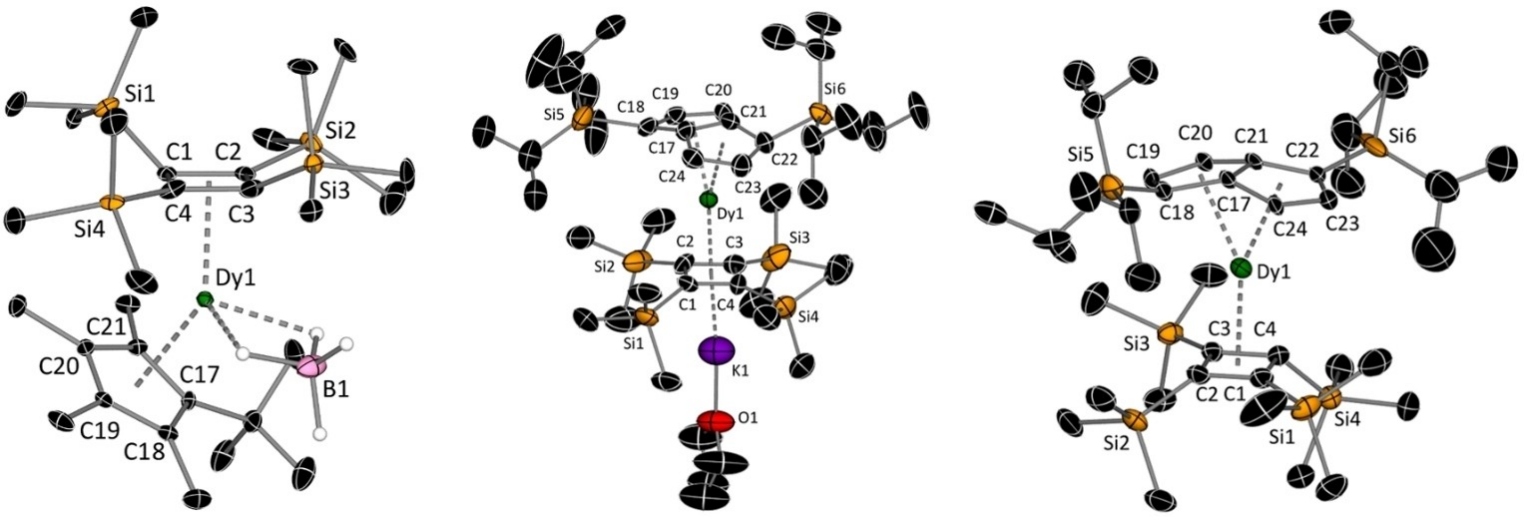
Thermal ellipsoid representations (30 % probability) of the molecular structure of the anion **1** (left), **2** (center) and the anion **3** (right). All carbon‐bound hydrogen atoms are omitted for clarity.

The molecular structures of all compounds were determined by X‐ray crystallography (Figure 1).^[35]^ The anion **1** consists of a bent metallocene motif in which the distance from dysprosium to the centroid of the cyclobutadienyl ligand is shorter by about 0.125 Å than the analogous distance to the cyclopentadienyl ligand, i.e. 2.2728(4) Å versus 2.3975(3) Å, respectively (Figures 1, S28, Tables S1, S2). Slightly asymmetric interactions of both ligands with dysprosium are revealed by Dy−C distances in the range 2.482(7)–2.523(6) Å for the cyclobutadienyl ligand and 2.667(6)–2.700(6) Å for the cyclopentadienyl ligand. The [BH_4_]^−^ ligand in **1** is disordered over two positions, however the hydrogen atoms were located based on residual electron density and freely refined. The Cb−Dy−Cp bending angle is 141.52(2)°. In the contact ion‐pair **2**, the Dy−Cb centroid distance is 2.306(11) Å, which is the same (within statistical error) as the analogous distances to the two centroids of the pentalene ligand, i.e. 2.282(5) Å and 2.300(4) Å, respectively (Figures 1, S29). However, whereas the Dy−C distances to the cyclobutadienyl ligand lie within a relatively narrow range of 2.47(3)–2.57(3) Å, the interaction of dysprosium with the pentalene carbon atoms is highly asymmetric, with short Dy−C distances of 2.385(8) Å and 2.400(8) Å to the bridgehead carbons C17 and C21, respectively, and much longer distances of 2.782(10) Å and 2.831(8) Å to the wing‐tip carbons C19 and C23. The structure of the anion **3** is similar, with Dy−Cb and Dy−Cp distances of 2.294(3), 2.301(6) and 2.296(4) Å, respectively (Figures 1, S30). The Dy−C distances to the Cb′′′′ ligand are 2.469(5)–2.580(5) Å, and 2.405(8)–2.825(14) Å to the Pn^†^ ligand.

Cyclobutadienyl complexes of the f‐elements are very rare, an observation related to the fact that cyclobutadiene pro‐ligands are unstable and known only with bulky silyl substituents.[^36^, ^37^] The silyl substituents are also prone to undergoing activation by deprotonation. Compounds **1**–**3** therefore expand the very small family of lanthanide and actinide complexes of the pristine Cb′′′′ ligand,[^38^, ^39^, ^40^, ^41^] and represent the first lanthanide metallocene‐like sandwich complexes of such a ligand. Viewed from the perspective of improving SMM properties, the broader significance of complex **1** is that if a method of removing the equatorial borohydride ligand can be devised, the (currently) hypothetical species [(η^4^‐Cb′′′′)Dy(η^5^‐C_5_Me_4_^*t*^Bu)] could be synthesized. In this complex, the crystal field should be stronger than in the 5* cation and, hence, its SMM properties should surpass those of the current state‐of‐the‐art.

The relatively short Dy−Cb′′′′ distance in **1** implies that the lanthanide interacts more strongly with the cyclobutadienyl ligand than with the cyclopentadienyl ligand, indicating that the cyclobutadienyl ligand should dominate the crystal field splitting imposed on the ^6^H_15/2_ ground multiplet of Dy^3+^. Comparing **1** with closely related [(Cp^*i*Pr5^)Dy(Cp*)(BH_4_)] (**4**, the precursor to the 5* cation), the Dy−Cp distance in **1** is longer by 0.015–0.033 Å than the analogous distances of 2.364(1) Å and 2.382(1) Å in **4**, suggesting that the Cb′′′′ ligand effectively pushes the [C_5_Me_4_^*t*^Bu]^−^ ligand away from dysprosium in **1**. A similar picture emerges when comparing the structures of **2** and **3** with that of [(η^5^‐Cp*)Dy(η^8^‐Pn^†^)] (**5**),^[42]^ for which the Dy−Cp* and Dy−Pn^†^ centroid distances are 2.344(5) Å and 2.235(3) Å, respectively. The Dy−Cb distances in **2** and **3** are shorter by approximately 0.04–0.05 Å than the Dy−Cp* distance in **4**, and the Dy−Pn^†^ distances are longer by 0.04–0.06 Å, suggesting that stronger interactions between Dy^3+^ and Cb′′′′ occur at the expense of the interactions with the Pn^†^ ligand.

**Figure 2:**
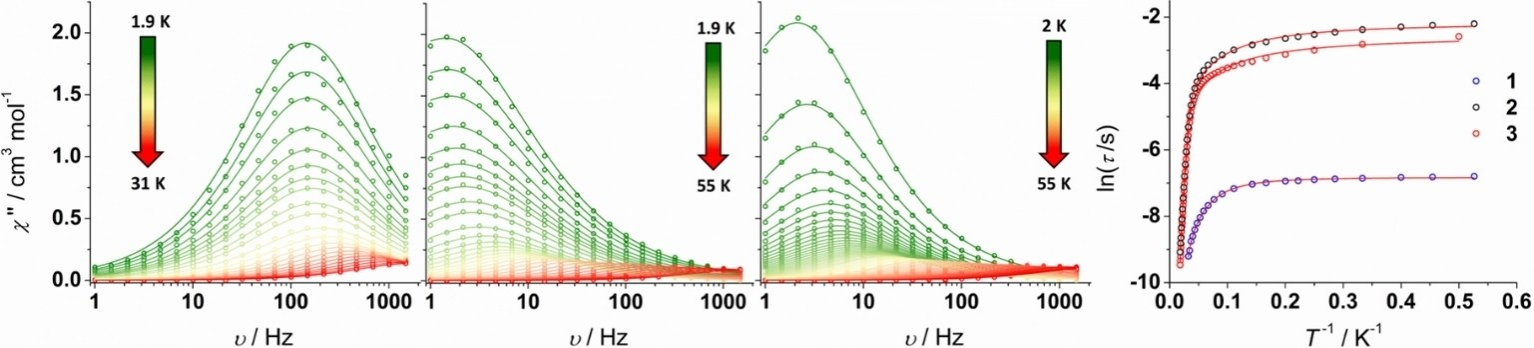
Frequency‐dependence of the imaginary component of the AC susceptibility for **1** (left), **2** (center left) and **3** (center right) at the temperatures shown, and temperature‐dependence of the magnetic relaxation times as ln *τ* versus *T* ^−1^ (right). Red lines are fits using the parameters stated in the text (with *U*_eff_=242 cm^−1^ for **1**). Data were collected in an AC field of 3 Oe and zero DC field.

To determine the extent to which the cyclobutadienyl ligand impacts on the SMM properties of **1**–**3**, each system was studied using AC magnetic susceptibility measurements in zero applied DC field (Figures S38, S39, S46, S47, S54, S55). The static DC field magnetic susceptibility was also measured for each compound in a 1 kOe field and found to be typical of a monometallic dysprosium(III) complex in each case (Figures S34–S37). The frequency‐dependence of the imaginary component of the AC susceptibility, *χ*′′(*ν*), for **1** shows well‐defined maxima in the temperature range 1.9–31 K (Figure 2), indicating SMM behavior. The frequency maximum associated with each temperature does not significantly shift position up to around 7 K, and at higher temperatures the maximum shifts to higher frequencies. In contrast to **1**, the qualitatively similar *χ*′′(*ν*) data for **2** and **3** consist of maxima in the range 1.9–55 K and 1.9–47 K, respectively. For all compounds, the AC susceptibility data suggest that the magnetic relaxation is dominated by quantum tunneling of the magnetization (QTM) at low temperatures, with activated relaxation mechanism(s) becoming dominant at higher temperatures.

From Cole–Cole plots of *χ*′′(*χ*′), the relaxation times (*τ*) were extracted using *α*‐parameters of 0.11–0.22, 0.06–0.35 and 0.09–0.27 for **1**–**3**, respectively (Figures S40–S43, S48–S51, S56–59, Tables S4–S6). Plotting ln(*τ*) against *T* ^−1^ for each compound confirmed that *τ* has a weak temperature dependence at low temperatures indicative of QTM, with a strong temperature dependence at higher temperatures being the hallmark of thermally activated relaxation (Figure 2). A curved crossover region at intermediate temperatures can be taken as evidence for the involvement of Raman relaxation processes, which is slightly more prominent in the case of **1**.

Fits of the data were obtained using the standard equation $\tau^{-1}=\;\tau_{0}^{-1}e^{-U_{eff}/k_{B}T}+CT^{n}+\tau_{QTM}^{-1}$ , where $\tau_{0}^{-1}$ , *C*, *n* and $\tau_{QTM}^{-1}$ are the attempt time, the Raman coefficient, the Raman exponent and the QTM rate, respectively. In the case of **1**, we found that there is no unique fit to the relaxation time data and that adjusted *R* ^2^ values of greater than 0.999 can be achieved with at least two sets of parameters. For example, an excellent (*R* ^2^=0.99948) fit was obtained using *U*_eff_=127(17) cm^−1^, *τ*_0_=9.0(6)×10^−7^ s, *C*=3.5(8) s^−1^ K^−*n*^, *n*=2.17(8) and *τ*_QTM_=1.10(1) ms (Figure S16). However, as we show below with a theoretical analysis, the energy barrier of 127(17) cm^−1^ is about half the energy of 242 cm^−1^ required for the system to relax via the first‐excited Kramers doublet, which would imply an under‐barrier process facilitated by anharmonic phonons. However, since the **g**‐tensors associated with this doublet have appreciable transverse components (*g_x_* =0.23, *g_y_* =0.38, *g_z_* =16.30), a barrier‐crossing transition via this route is probable, suggesting that the *U*_eff_ value of 127(17) cm^−1^ obtained from the fit is spurious. A second fit with *U*_eff_ fixed at 242 cm^−1^ yielded a different (although reasonable) pre‐exponential factor of *τ*_0_=6.0(9)×10^−9^ s, very similar Raman parameters of *C*=1.9(3) s^−1^ K^−*n*^, *n*=2.39(5) and essentially the same *τ*_QTM_ of 1.08(1) ms, with *R* ^2^=0.99918 (Figures 2, S4). The fits for **2** and **3** are less complicated and yielded the following parameters: *U*_eff_=213(3) cm^−1^, *τ*_0_=4.76(5)×10^−7^ s, *C*=0.34(8) s^−1^ K^−*n*^, *n*=1.58(8) and *τ*_QTM_=0.114(5) s for **2**, and; *U*_eff_=222(3) cm^−1^, *τ*_0_=2.69(3)×10^−7^ s, *C*=0.8(2) s^−1^ K^−*n*^, *n*=1.38(9) and *τ*_QTM_=0.076(7) s for **3** (Figures 2, S52, S60). These *U*_eff_ values are also comparable to the energies calculated for the first‐excited Kramers doublets (see below).

The low Raman exponents of *n*≈2 determined from fits of the AC susceptibility are seemingly a hallmark of dysprosium metallocene SMMs.[^9^, ^21^] However, when the rate of spin‐lattice relaxation is dependent on *T* ^2^, phonon‐bottleneck effects could operate.[^43^, ^44^, ^45^] Although unlikely in a system with *S* >1/2, magnetic dilution experiments were undertaken to investigate this possibility. The yttrium compounds [Y{η^4^‐C_4_(SiMe_3_)_4_}(η^5^‐C_5_Me_4_^*t*^Bu)(κ^2^‐BH_4_)][Na(15‐crown‐5)(THF)_2_] ([**4**][Na(15‐crown‐5)(THF)_2_]) and [Y{η^4^‐C_4_(SiMe_3_)_4_}{η^8^‐C_8_(Si(^*i*^Pr_3_)_2_)H_4_}][K(18‐crown‐6)(THF)_2_] ([**6**][K(18‐crown‐6)(THF)_2_]) were therefore synthesized and characterized by IR spectroscopy, multinuclear NMR spectroscopy and X‐ray crystallography (Figures S1–S27, S31–S33, Tables S1, S3). Both **4** and **6** are isostructural to the dysprosium complexes in **1** and **3**, allowing magnetic dilution measurements to be undertaken on samples denoted **1 a** and **3 a**, respectively. The attempted synthesis of the isostructural yttrium analogue of **2** instead yielded [Y{η^4^‐C_4_(SiMe_3_)_4_}{η^8^‐C_8_(Si(^*i*^Pr_3_)_2_)H_4_}K(THF)_2_] (**5**), with two THF ligands per potassium, and with the molecules crystallizing in a different crystallographic space group with significantly different unit cell parameters. Dilution measurements on **2** were, therefore, not undertaken.

The AC susceptibility data on **1 a** (15 % dilute) and **3 a** (10 % dilute) yielded relaxation times with a temperature dependence that could be fitted with Orbach, Raman and QTM terms. As with **1**, fits of *τ* vs. *T* ^−1^ for **1 a** were possible with more than one set of parameters. The fit with *U*_eff_ fixed at 242 cm^−1^ produced *τ*_0_=2.2(3)×10^−8^ s, *C*=0.18(2) s^−1^ K^−*n*^, *n*=3.01(4) and *τ*_QTM_=0.0182(6) s. For **3 a**, *U*_eff_=233(8) cm^−1^, *τ*_0_=1.9(5)×10^−7^ s, *C*=0.04(5) s^−1^ K^−*n*^, *n*=2.3(4) and *τ*_QTM_=0.6(4) s. The effective energy barrier for **3 a** is therefore very similar to that of the non‐dilute analogue. In both dilute samples, the rate of QTM is significantly reduced, consistent with the effects of intermolecular dipolar exchange facilitating relaxation in the non‐dilute samples. Comparison of the *τ* values for **1** and **3** with those for **1 a** and **3 a**, respectively, show that the relaxation is slower in the dilute samples at any given temperature in the measured ranges. This observation indicates that phonon bottleneck effects do not play a part in the magnetic relaxation for **1** and **3** (and probably also **2**), despite the Raman parameter taking values of *n*≈2.

**Figure 3:**
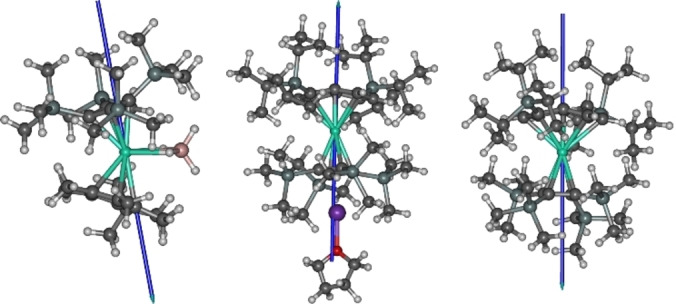
The principal magnetic axes (blue arrows) of the ground Kramers doublets in **1–3**. Dy=green, K=purple, Si=light gray, O=red, C=dark gray, B=pink, H=white.

The similar, moderate *U*_eff_ values in **1**–**3** are likely to be a consequence of the non‐negligible equatorial crystal field originating from the [BH_4_]^−^ ligand in **1** and from the wing‐tip carbon atoms in **2** and **3**. To gain further insight into the relaxation phenomena in **1**–**3**, multireference ab initio calculations were conducted.[^46^, ^47^, ^48^, ^49^] The energies and principal components of the **g**‐tensors of the eight lowest Kramers doublets (KDs) corresponding to the crystal‐field‐split ^6^H_15/2_ ground multiplets of the Dy^3+^ ion calculated for **1**–**3** are listed in Tables S9–S11. The principal magnetic axes are shown in Figure 3.

The ground doublet of **1** is strongly axial, with the first excited doublet calculated to occur at 242 cm^−1^. The principal magnetic axis of the first‐excited doublet is rotated by 8.4° compared to the principal axis of the ground doublet. This suggests that an Orbach mechanism for the relaxation of magnetization will take place via this doublet, giving an effective barrier height of 242 cm^−1^. It is important to note that the direction of the principal magnetic axis of the ground doublet follows the Dy−Cb axis rather than the Dy−Cp axis. This indicates that the crystal field induced by the cyclobutadienyl ligand does indeed dominate over that induced by the cyclopentadienyl ligand, further suggesting that the former type of ligand can produce a stronger crystal field splitting.

Consistent with the AC susceptibility data, the electronic structures of **2** and **3** are very similar. The ground doublets are strongly axial and the axiality is also retained in the lowest excited doublets. The **g**‐tensor of the second excited doublet shows notable transverse components and in the third‐excited doublet the transverse components are very significant. This suggests that the relaxation of magnetization by an Orbach mechanism would take place via the second or third excited doublet. However, in both cases the effective barrier heights determined from the relaxation data are close to the energy of the first excited doublets, which lie at 236 cm^−1^ and 228 cm^−1^ for **2** and **3**, respectively. This indicates that the axiality of the crystal field is somewhat overestimated in the calculations.

**Figure 4:**
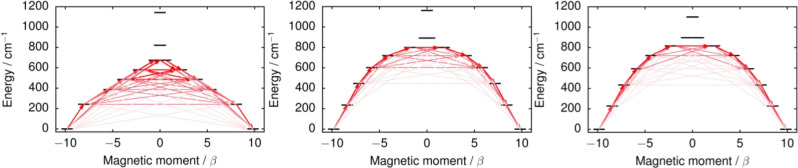
Calculated effective ab initio barriers for the relaxation of magnetization in **1**, **2** and **3**. Stronger red arrows indicate larger absolute value of the transition magnetic moment matrix elements between the respective states. Transitions involving higher‐energy states not involved in the relaxation mechanism are omitted for clarity.

Qualitative relaxation barriers for **1**–**3** were constructed using a well‐established ab initio methodology in which relaxation pathways from one component of the ground doublet with maximum magnetization to its time‐reversed counterpart are considered.^[50]^ The barriers are shown in Figure 4 and the quantitative values of the transition magnetic moment matrix element are listed in Tables S19–S21. In all cases a barrier‐like structure is retained up to the sixth excited doublet indicating a dominant axial crystal field. In the case of **1**, the barrier should be crossed at the first excited doublet, consistent with the analysis of the **g** tensors. In both **2** and **3**, the calculations predict that the barrier is crossed at the earliest in the second‐excited doublet and at the latest in the third‐excited doublet, again consistent with the analysis of the **g**‐tensors. However, based on the experimental data, the barrier is most likely crossed already at the first excited doublet; the transition magnetic moment for this transition is either underestimated in the calculations or the deviations is due to intricacies of the spin‐phonon interactions not properly accounted for by the relaxation model based on transition dipole magnetic moments.

Regarding the effective energy barrier for **1**, whilst under‐barrier relaxation in an SMM has been justified by a finite phonon lifetime due to anharmonic phonon‐phonon interaction,^[51]^ it has also been shown that under‐barrier relaxation can result from a Raman mechanism with an exponential temperature‐dependence.^[52]^ However, in the absence of direct experimental measure of the multiplet splitting, it cannot be verified whether the calculations underestimate the splitting or whether an under‐barrier mechanism is operational in **1**. Considering the uncertainty in the immediate crystal field around the Dy^III^ ion due to the optimized hydrogen atom positions in the [BH_4_]^−^ ligand, possible error in the calculated multiplet splitting seems the most likely explanation. Note that poor fits of ln *τ* vs. *T* ^−1^ are obtained if the *U*_eff_ values for **2** and **3** are fixed at the energies of the second‐excited Kramers doublets (i.e. 447 and 434 cm^−1^, respectively), particularly at lower temperatures (Figures S52, S60).

To explore further the nature of the crystal field environment around the Dy^3+^ ions, the ab initio crystal field parameters were calculated for complexes **1**–**3**. These parameters are listed in Tables S12–S14 using the Iwahara–Chibotaru definition of the equivalent operators.[^53^, ^54^, ^55^] The parameters can be understood qualitatively by considering the leading‐order rank *k*=2 parameters. The diagonal *B*_20_ parameter is negative and relatively large for all complexes **1**–**3**, stabilizing the *M*=±15/2 states.

However, the off‐diagonal *B*_2±1_ and *B*_2±2_ parameters also make appreciable contributions reducing the overall axiality. Here, we introduce the *theoretical axiality factor*, *Z*, defined as the ratio |*B*_20_|/|*B*_2±2_|, to provide a measure of the SMM performance. The parameter *Z* is reminiscent of (indeed, inversely proportional to) the rank‐two rhombicity parameter (*E*/*D*) used in the standard EPR spin Hamiltonian.[^56^, ^57^] Since the benchmark SMM [(Cp^*i*Pr5^)Dy(Cp*)]^+^ has *Z*=39.5,^[31]^ the *relative theoretical axiality factor*, *Z*_rel_=*Z*/39.5, may also be defined (Table 1). Values of *Z*=2.0, 3.8 and 5.2, and *Z*_rel_=0.050, 0.096 and 0.132 are calculated for **1**–**3**, respectively.

**Table 1:** Selected SMM parameters.

Compound	*U*_eff_ [cm^−1^]	*Z* ^[a]^	*Z*_rel_^[b]^	Reference
[(Cp*)Dy(Cp^iPr5^)]^+^	1541(11)	39.5	1	[31]
**1**	242^[c]^	1.96	0.050	This work
**2**	213(3)	3.79	0.096	This work
**3**	222(3)	5.23	0.132	This work
**7**	7(1)	1.81	0.046	[31]
**8**	188	2.88	0.073	[41]

[a] Defined as |*B*_20_|/|*B*_2±2_|. [b] Defined as *Z*/39.5. [c] Fixed from computational studies.

The observation that the axiality factors for **1**–**3** are much lower than the values for [(Cp^*i*Pr5^)Dy(Cp*)]^+^ can be rationalized readily. In the case of **1**, the geometry of the complex is strongly bent and the borohydride ligand occupies an equatorial position. In **2** and **3**, the Pn^†^ ligand envelops the Dy^3+^ ion in a manner that introduces non‐axial contributions to the crystal field via the wing‐tip carbon atoms. The ion‐contact interaction of the cyclobutadienyl ligand with potassium ion in **2** also seems to reduce *Z* and *Z*_rel_. However, it is noteworthy that *Z* and *Z*_rel_ for [(Cp^*i*Pr5^)Dy(Cp*)(BH_4_)] (**7**) are 1.81 and 0.046 (Table S15), i.e. lower than the values calculated for **1**. Similarly, the *Z* and *Z*_rel_ values of 2.88 and 0.073 calculated for [(η^5^‐Cp*)Dy(η^8^‐Pn^†^)] (**8**), which has a *U*_eff_ of 188 cm^–1^,^[42]^ are lower than the values calculated for **2** and **3**, which again shows that cyclobutadienyl ligands are capable of producing a stronger axial crystal field than cyclopentadienyl ligands.

The small values of *Z* and *Z*_rel_ for **1**–**3** are also reflected in their magnetic hysteresis properties. At 1.9 K and with field sweep rates in the range *B*=1.1–8.5 mT s^−1^, very narrow S‐shaped loops were observed for **1** whereas opening in the loops were observed for **2** and **3** between 0–1 T (Figures S45, S53, S61). Small openings in the loops were observed for the dilute sample **1 a** and openings in the the hysteresis loops for **3 a** were observed in the temperature range 1.9–5 K (Figures S69, S77).

## Conclusion

In summary, our analysis of the dysprosium complexes **1**–**3** has shown that the dianionic cyclobutadienyl ligand [η^4^‐Cb′′′′]^2−^ can effectively replace cyclopentadienyl ligands in structurally similar metallocene SMMs, resulting in larger experimental *U*_eff_ values. The effect originates from relatively short dysprosium–cyclobutadienyl distances, which allow the ligand to dominate the crystal field splitting experience by the ^6^H_15/2_ multiplet. A computational study of the electronic structure of **1**–**3** provides supporting evidence that cyclobutadienyl ligands are indeed capable of producing stronger crystal fields than cyclopentadienyl ligands. The increase in the barrier of [(η^4^‐Cb′′′′)Dy(η^5^‐C_5_Me_4_^*t*^Bu)(BH_4_)]^−^ (**1**) relative to that of [(Cp^*i*Pr5^)Dy(Cp*)(BH_4_)] (**4**) is significant in light of the dramatic enhancement in SMM performance observed when **4** is converted into the current benchmark SMM [(Cp^*i*Pr5^)Dy(Cp*)]^+^. Thus, if complexes of the type [(η^4^‐Cb^R^)_2_Dy]^−^ or [(η^4^‐Cb^R^)Dy(η^5^‐Cp^R^)] could be stabilized in sufficiently axial (i.e. near‐linear) geometries, it is possible that they would display stronger crystal field splitting than any previously characterized dysprosium complex, potentially with *Z*_rel_ >1. While the SMM properties of such a hypothetical species would ultimately depend on how strongly the spin system interacts with the lattice vibrations,^[32]^ a [(η^4^‐Cb^R^)_2_Dy]^−^ complex is a clear and viable candidate to surpass the performance record set by [(Cp^*i*Pr5^)Dy(Cp*)]^+^.

1

## Supporting information

As a service to our authors and readers, this journal provides supporting information supplied by the authors. Such materials are peer reviewed and may be re‐organized for online delivery, but are not copy‐edited or typeset. Technical support issues arising from supporting information (other than missing files) should be addressed to the authors.

Supporting Information

## Data Availability

Additional research data supporting this publication are available as supplementary information at 10.25377/sussex.19232580.
