# Supplementary material for: Dominance of Cyclobutadienyl Over Cyclopentadienyl in the Crystal Field Splitting in Dysprosium Single‐Molecule Magnets
Source: Angew Chem Int Ed Engl. 2022 Feb 26;61(17):e202200525. doi: 10.1002/anie.202200525 (PMC9302998; doi:10.1002/anie.202200525)
Supplement: Supplementary file 1 — Supporting Information [file ANIE-61-0-s001.pdf]

Angewandte Chemie
Eine Zeitschrift der Gesellschaft Deutscher Chemiker

Supporting Information

Dominance of Cyclobutadienyl Over Cyclopentadienyl in the Crystal Field Splitting in Dysprosium Single-Molecule Magnets

J. P. Durrant, B. M. Day, J. Tang, A. Mansikkamäki, R. A. Layfield**

Supporting Information

Contents

Synthesis and Characterization	S1-S20
X-ray Crystallography	S21-S29
Magnetic Property Measurements	S30-S79
Computational Details	S80-S98
References	S99

General Considerations

All reactions were carried out under rigorous anaerobic, anhydrous conditions under argon or nitrogen atmospheres and standard Schlenk or glove-box techniques. All solvents were refluxed over an appropriate drying agent for a minimum of three days (molten potassium for benzene, THF and Na/K alloy for hexane), and then distilled, degassed via a minimum of three freeze-pump-thaw cycles, and stored in ampoules over potassium mirrors (benzene and hexane) or activated 4 Å molecular sieves (THF). NMR spectra were recorded on a Varian VNMR S400 spectrometer operating at 30°C unless otherwise stated at frequencies of 400 MHz (^{1}H), 128 MHz (^{11}B), 100 MHz (^{13}C), 106 MHz (^{23}Na) and 80 MHz (^{29}Si). Elemental analyses were carried out at MEDAC Ltd., Surrey, U.K or Microanalytisches Labor Pascher, Germany. 15-crown-5 was purchased from Sigma-Aldrich, degassed and stored over activated 4 Å molecular sieves in an argon glovebox prior to use. 18-Crown-6 was purchased from Sigma-Aldrich, sublimed and stored in an argon glovebox prior to use. Literature procedures were used to synthesize [M{η^4-$C_4(SiMe_3)_4$}(κ^3-$BH_4)_2$(THF)Na], [M{η^4-$C_4(SiMe_3)_4$}(κ^3-$BH_4)_2$(THF)K] (M = Dy, Y), [NaCp^{Me4tBu}] and [K_2Pn(Si^{i}Pr$_{32}$)].[1–3]

Synthesis of Dysprosium Complexes

Synthesis of [Dy{η^4-$C_4(SiMe_3)_4$}(η^5-$C_5Me_4{}^tBu$)(κ^2-BH_4)][Na(15-c-5)(THF)$_2$] ([1][Na(15-c-5)(THF)$_2$])

An off-white solution of [NaCp^{Me4tBu}] (77 mg, 0.39 mmol) in THF (10 mL) was added dropwise to a dark orange solution of [Dy{η^4-$C_4(SiMe_3)_4$}(κ^3-$BH_4)_2$(THF)Na] (242 mg, 0.39 mmol) in THF (20 mL). The resulting dark red solution was left to stir for 20 hours, by which time a white precipitate had deposited. The reaction mixture was filtered, and the solvent removed under reduced pressure to yield an orange powder (195 mg). Red crystals were obtained by layering hexane onto a THF solution of the orange powder and storing the solution at –40 °C for three days, however these crystals were extremely temperature sensitive and would re-dissolve within seconds of being removed from the freezer. Publication-quality X-ray crystallography data could not be obtained, however the composition of the molecule was identified as [Dy{η^4-$C_4(SiMe_3)_4$}{η^5-$C_5(Me_4{}^tBu)$}(κ^3-BH_4)Na(THF)$_4$]. By adding 15-crown-5 (9 mg, 0.041 mmol) dropwise to a dark red solution of [Dy{η^4-$C_4(SiMe_3)_4$}{η^5-$C_5(Me_4{}^tBu)$}(κ^3-BH_4)Na(THF)$_4$] (30 mg, 0.041 mmol) in THF (2 mL), a solution was

obtained and left to stand overnight. The resulting dark red solution was filtered, layered with hexane and stored at –40 °C for three days, resulting in formation of red crystals of [**1**][Na(15-crown-5)(THF)$_2$] suitable for X-ray crystallography (32 mg, 73 % isolated yield). **FTIR ($\bar{\nu}$/cm^{-1}):** 3000-2850 (m, b, C–H), 2450 (m, b, B–H$_T$), 2300-2100 (m, b, B–H$_B$). **Elemental analysis (%):** found (calculated) for $C_{47}H_{97}DyBNaSi_4O_7$: C 51.53 (52.13); H 9.30 (9.03).

Synthesis of [Dy{η^4-C$_4$(SiMe$_3$)$_4$}{η^8-C$_8$(Si(^{i}Pr$_3$)$_2$)H$_4$}K(THF)] (2)

A dark purple solution of [K$_2$Pn(Si(^{i}Pr$_3$)$_2$)] (230 mg, 0.47 mmol) in THF (10 mL) was added dropwise to an orange solution of [Dy{η^4-C$_4$(SiMe$_3$)$_4$}(κ^3-BH$_4$)$_2$(THF)K] (300 mg, 0.47 mmol) in THF (15 mL) cooled to –78 °C. The resulting dark red solution was warmed to room temperature and stirred overnight, by which time a white precipitate had deposited. The reaction mixture was filtered, and the solvent removed under reduced pressure to yield a crude orange powder, subsequently identified as (**2**) (406 mg, 85 % isolated yield). Orange crystals of **2** suitable for X-ray crystallography were obtained by storing a saturated hexane solution at –40 °C for three days (268 mg, 56 % isolated yield). **FTIR ($\bar{\nu}$/cm^{-1}):** 3000-2850 (m, b, C–H). **Elemental analysis (%):** found (calculated) for $C_{46}H_{90}DyKSi_6O$: C 54.16 (53.68); H 9.09 (8.81).

Synthesis of [Dy{η^4-C$_4$(SiMe$_3$)$_4$}{η^8-C$_8$(Si(^{i}Pr$_3$)$_2$)H$_4$}][K(18-c-6)(THF)$_2$] ([3][K(18-c-6)(THF)$_2$])

A solution of 18-crown-6 (33 mg, 0.13 mmol) in THF (3 ml) was added dropwise into a dark red solution of **2** (130 mg, 0.13 mmol) in THF (10 mL) and stirred overnight. The resulting dark red reaction mixture was filtered, and the solvent removed under reduced pressure to yield a crude orange-red powder subsequently identified as [**3**][K(18-crown-6)(THF)$_2$] (143 mg, 83 % isolated yield). Red crystals of [**3**][K(18-crown-6)(THF)$_2$] suitable for X-ray crystallography were obtained by washing the crude material with hexane, and storing a saturated THF solution layered with hexane at –40 °C for three days (112 mg, 65 % isolated yield). **FTIR ($\bar{\nu}$/cm^{-1}):** 3000-2850 (m, b, C–H). **Elemental analysis (%):** found (calculated) for $C_{58}H_{114}DyKSi_6O_7$, [**3**][K(18-crown-6)(THF)$_2$](−THF): C 53.43 (53.85); H 8.87 (8.88).

Synthesis of Yttrium Complexes

Synthesis of [Y{η^4-C$_4$(SiMe$_3$)$_4$}(η^5-C$_5$Me$_4$^{t}Bu)(κ^2-BH$_4$)][Na(15-c-5)(THF)$_2$] ([4][Na(15-crown-5)(THF)$_2$])

Compound [**4**][Na(15-crown-5)(THF)$_2$] was synthesised by following the same procedure as for [**1**][Na(15-crown-5)(THF)$_2$] using [NaCp^{Me4tBu}] (53 mg, 0.26 mmol) and [Y{η^4-C$_4$(SiMe$_3$)$_4$}(κ^3-BH$_4$)$_2$(THF)Na] (150 mg, 0.26 mmol) in THF (20 mL). The reaction mixture was filtered, and the solvent removed in vacuo to yield an orange powder (106 mg). Red crystals were obtained by layering hexane onto a THF solution of the orange powder and storing the solution at –40 °C for three days. However, these crystals almost immediately re-dissolve upon removal from the freezer. Publication-quality X-ray crystallography data could not be obtained, however the basic atomic connectivity was determined to be [Y{η^4-C$_4$(SiMe$_3$)$_4$}{η^5-C$_5$(Me$_4$^{t}Bu)}(κ^2-BH$_4$)Na(THF)$_4$]. By adding 15-crown-5 (44 mg, 0.20 mmol) dropwise to a dark red solution of [Y{η^4-C$_4$(SiMe$_3$)$_4$}{η^5-C$_5$(Me$_4$^{t}Bu)}(κ^2-BH$_4$)Na(THF)$_4$] (106 mg, 0.17 mmol) in THF (3 mL), a solution was obtained and left to stand overnight. The resulting dark red solution was filtered, layered with hexane and stored at –40 °C for three days, resulting in formation of red crystals of [**4**][Na(15-crown-5)(THF)$_2$] suitable for X-ray crystallography (107 mg, 64 % isolated yield). **FTIR ($\bar{\nu}$/cm^{-1}):** 3000-2850 (m, b, C-H), 2400-2300 (w, d, B-H$_T$), 2150-2000 (w, bs, B-H$_B$). **Elemental analysis (%):** found (calculated) for $C_{43}H_{89}YBNaSi_4O_6$, [**4**][Na(15-crown-5)(THF)$_2$] (−THF): C 55.22 (55.11); H 9.58 (9.57).

Multinuclear NMR spectroscopy of [**4**][Na(15-crown-5)(THF)$_2$] revealed the presence of two species in solution, which we attribute to the formation of separated and contact-ion pairs in a ratio of approximately 3:1. **^{1}H NMR** (δ/ppm, D$_8$-THF): Major: 3.69 (s, C<u>H$_2$</u> crown, 24H, overlapped with minor component), 2.42 (s, Me$_2$, 6H), 2.12 (s, Me$_2$, 6H), 1.27 (s, ^{t}Bu, 9H), 0.10 (s, SiMe$_3$, 36H). Minor: 3.69 (s, CH$_2$ crown, 24H, overlapped with major component)), 2.18 (s, Me$_2$, 3H), 1.97 (s, Me$_2$, 3H), 1.35 (s, ^{t}Bu, 4H), 0.14 (s, SiMe$_3$, 26H). **^{13}C{^{1}H} NMR** (δ/ppm, D$_8$-THF): Major: 129.04 (<u>C</u>$_4$ ring), 117.63 (<u>C</u>$_5$ ring), 116.49 (<u>C</u>$_5$ ring), 69.71 (<u>C</u>H$_2$), 68.38 (<u>C</u>H$_2$O), 34.76 (<u>C</u>(CH$_3$)$_3$), 33.16 (C(<u>C</u>H$_3$)$_3$), 26.53 (<u>C</u>H$_2$), 17.22 (<u>Me</u>$_2$), 13.40 (<u>Me</u>$_2$), 5.43 (Si<u>Me</u>$_3$). Signals corresponding to

the C_5 ring carbon attached to the ^{t}Bu group and for the minor component could not be observed, except for a signal at 5.35 (Si<u>Me</u>$_3$). **^{11}B{^{1}H} NMR** (δ/ppm, D_8-THF): Major: −24.87 (s, FWHM = 55 Hz, BH_4). Minor: −20.85 (s, FWHM = 154 Hz, BH_4). **^{11}B NMR** (δ/ppm, D_8-THF): Major: −24.86 (quint., $^1J_{BH}$ = 85 Hz, BH_4). Minor: −26.67 (s, BH_4). **^{29}Si{^{1}H} NMR** (δ/ppm, D_8-THF): −21.07. **^{23}Na NMR** (δ/ppm, D_8-THF): −5.82 (FWHM = 362 Hz).

Synthesis of magnetically dilute [1][Na(15-crown-5)(THF)$_2$] (1a)
The magnetically dilute sample was synthesized by combining [**1**][Na(15-crown-5)(THF)$_2$] and [**4**][Na(15-crown-5)(THF)$_2$] in a 9:1 molar ratio, followed by recrystallization from a saturated THF solution layered with hexane at –40 °C. ICP-MS revealed the dysprosium content to be 15%.

^{1}H NMR (δ/ppm, D_8-THF): Major: 2.45 (s, Me_2, 6H), 2.16 (s, Me_2, 6H), 1.60 (s, C<u>H$_2$</u> crown, 20H), 1.30 (s, ^{t}Bu, 9H), 0.14 (s, $SiMe_3$, 36H). **^{13}C{^{1}H} NMR** (δ/ppm, D_8-THF): 129.8 (d, $^1J_{YC}$ = 5.0 Hz, <u>C</u>$_4$ ring), 127.93 (d, $^1J_{YC}$ = 0.8 Hz, <u>C</u>$_5$ ring), 117.67 (d, $^1J_{YC}$ = 1.5 Hz, <u>C</u>$_5$ ring), 116.54 (d, $^1J_{YC}$ = 1.5 Hz, <u>C</u>$_5$ ring), 35.80 (<u>C</u>(CH_3)$_3$), 33.21 (C(<u>C</u>H$_3$)$_3$), 17.27 (<u>Me</u>$_2$), 13.46 (<u>Me</u>$_2$), 5.49 (Si<u>Me</u>$_3$). **^{11}B{^{1}H} NMR** (δ/ppm, D_8-THF): Major: −20.11 (s, FWHM = 62 Hz, BH_4). Minor: −24.79 (s, FWHM = 27 Hz, BH_4). **^{11}B NMR** (δ/ppm, D_8-THF): Major: −20.11 (quint., $^1J_{BH}$ = 90 Hz, BH_4). Minor: −24.79 (quint., $^1J_{BH}$ = 79 Hz, BH_4). **^{29}Si{^{1}H} NMR** (δ/ppm, D_8-THF): −21.04. **FTIR ($\bar{\nu}$/cm^{-1}):** 3000-2850 (m, b, C-H), 2400-2300 (w, d, B-H_T), 2150-2000 (w, bs, B-H_B).

Synthesis of [Y{η^4-C$_4$(SiMe$_3$)$_4$}{η^8-C$_8$(Si(^{i}Pr$_3$)$_2$)H$_4$}K(THF)$_2$] (5)
Compound **5** was synthesised by following the same procedure as for **2** using [K_2Pn(Si(^{i}Pr$_3$)$_2$)] (229 mg, 0.46 mmol) and [Y{η^4-C$_4$(SiMe$_3$)$_4$}(κ^3-BH$_4$)$_2$(THF)K] (265 mg, 0.46 mmol). The reaction mixture was filtered, and the solvent removed in vacuo to yield a crude orange powder, subsequently identified as (**5**) (367 mg, 77 % isolated yield). Orange crystals of **5** suitable for X-ray crystallography were obtained by storing a saturated hexane solution at –40 °C for three days (218 mg, 46 % isolated yield).

^{1}H NMR (δ/ppm, D_8-THF): 6.81 (dd, $^1J_{YH}$ = 0.8, $^3J_{HH}$ = 3.0 Hz, Pn <u>H</u>, 2H), 5.19 (d, $^3J_{HH}$ = 3.1 Hz, Pn <u>H</u>, 2H), 3.62 (m, THF, 5H), 1.78 (m, THF, 5H), 1.24 (m, ^{i}Pr C<u>H</u>), 1.06 (d, $^3J_{HH}$ = 7.4 Hz, ^{i}Pr C<u>H$_3$</u>, 18H), 1.03 (d, $^3J_{HH}$ = 7.3 Hz, ^{i}Pr C<u>H$_3$</u>, 18H), 0.08 (s, Si<u>Me$_3$</u>, 36H). **^{13}C{^{1}H} NMR** (δ/ppm, D_8-THF): 144.06 (Pn bridgehead <u>C</u>), 130.83 (Pn <u>C</u>H), 130.68 (d, $^1J_{YC}$ = 5.0 Hz, <u>C</u>$_4$ ring), 98.48 (Pn <u>C</u>H), 89.26 (Pn <u>C</u>-Si), 68.38 (THF), 26.54 (THF), 20.89 (^{i}Pr <u>C</u>H$_3$), 20.52 (^{i}Pr <u>C</u>H$_3$), 13.77 (^{i}Pr <u>C</u>H), 5.26 (Si<u>Me</u>$_3$). **^{29}Si{^{1}H} NMR** (δ/ppm, D_8-THF): 0.55 (Pn <u>Si</u>^{i}Pr$_3$), −23.66 (<u>Si</u>Me$_3$). **FTIR ($\bar{\nu}$/cm^{-1}):** 3000-2850 (m, b, C–H). **Elemental analysis (%):** found (calculated) for $C_{50}H_{98}YKSi_6O_2$: C 58.41 (58.43); H 9.71 (9.61).

Synthesis of [Y{η^4-C$_4$(SiMe$_3$)$_4$}{η^8-C$_8$(Si(^{i}Pr$_3$)$_2$)H$_4$}][K(18-crown-6)(THF)$_2$] ([6][K(18-crown-6)(THF)$_2$])
Compound [**6**][K(18-crown-6)(THF)$_2$] was synthesised by following the same procedure as for [**3**][K(18-crown-6)(THF)$_2$] using 18-crown-6 (51 mg, 0.19 mmol) and **5** (200 mg, 0.19 mmol). The resulting dark red reaction mixture was filtered, and the solvent removed in vacuo to yield a crude orange-red powder subsequently identified as [**6**][K(18-crown-6)(THF)$_2$] (198 mg, 79 % isolated yield). Red crystals of [**6**][K(18-crown-6)(THF)$_2$] were obtained by washing the crude material with hexane, and storing a saturated THF solution layered with hexane at –40 °C for three days (148 mg, 59 % isolated yield).

^{1}H NMR (δ/ppm, D_8-THF): 6.81 (dd, $^1J_{YH}$ = 0.9, $^3J_{HH}$ = 3.1 Hz, Pn <u>H</u>, 2H), 5.19 (d, $^3J_{HH}$ = 3.1 Hz, Pn <u>H</u>, 2H), 3.62 (s, C<u>H$_2$</u> crown, 24H), 1.24 (m, ^{i}Pr C<u>H</u>), 1.06 (d, $^3J_{HH}$ = 7.3 Hz, ^{i}Pr C<u>H$_3$</u>, 18H), 1.03 (d, $^3J_{HH}$ = 7.3 Hz, ^{i}Pr C<u>H$_3$</u>, 18H), 0.08 (s, Si<u>Me$_3$</u>, 36H). **^{13}C{^{1}H} NMR** (δ/ppm, D_8-THF): 144.05 (d, $^1J_{YC}$ = 2.3 Hz, Pn bridgehead <u>C</u>), 130.82 (Pn <u>C</u>H), 130.72 (d, $^1J_{YC}$ = 5.9 Hz, <u>C</u>$_4$ ring), 98.47 (Pn <u>C</u>H), 89.28 (Pn <u>C</u>-Si), 71.30 (<u>C</u>H$_2$ crown), 68.38 (THF), 26.55 (THF), 20.91 (^{i}Pr <u>C</u>H$_3$), 20.53 (^{i}Pr <u>C</u>H$_3$), 13.78 (^{i}Pr <u>C</u>H), 5.28 (Si<u>Me</u>$_3$). **^{29}Si{^{1}H} NMR** (δ/ppm, D_8-THF): 0.54 (Pn <u>Si</u>^{i}Pr$_3$), −23.65 (<u>Si</u>Me$_3$). **FTIR ($\bar{\nu}$/cm^{-1}):** 3000-2850 (m, b, C–H). Despite several attempts, satisfactory elemental analysis could not be obtained for this compound, the samples being consistently low in carbon. A representative result is: found (%) (calculated) for $C_{62}H_{122}YKSi_6O_8$: C 53.65 (57.63); H 8.94 (9.52). The NMR

spectroscopy data of this compound are, however, consistent with the molecular structure determined by X-ray crystallography.

Synthesis of magnetically dilute [3][K(18-crown-6)(THF)$_2$] (3a)

The magnetically dilute sample was synthesized by combining [**6**][K(18-crown-6)(THF)$_2$] and [**3**][K(18-crown-6)(THF)$_2$] in a 9:1 molar ratio, followed by recrystallization from a saturated THF solution layered with hexane at –40 °C. ICP-MS revealed the dysprosium content to be 9.7%.

^{1}H NMR (δ/ppm, D$_8$-THF): 6.83 (d, $^{3}J_{HH}$ = 2.6 Hz, Pn H, 2H), 5.20 (d, $^{3}J_{HH}$ = 3.0 Hz, Pn H, 2H), 3.97 (s, CH$_2$ crown, 24H), 3.62 (m, THF, 10H), 1.78 (m, THF, 10H), 1.25 (m, ^{i}Pr CH), 1.07 (d, $^{3}J_{HH}$ = 7.3 Hz, ^{i}Pr CH$_3$, 18H), 1.04 (d, $^{3}J_{HH}$ = 7.2 Hz, ^{i}Pr CH$_3$, 18H), 0.09 (s, SiMe$_3$, 36H). **^{13}C{^{1}H} NMR** (δ/ppm, D$_8$-THF): 144.07 (d, $^{1}J_{YC}$ = 2.7 Hz, Pn bridgehead C), 130.85 (Pn CH), 130.74 (d, $^{1}J_{YC}$ = 5.6 Hz, C$_4$ ring), 98.49 (Pn CH), 89.29 (Pn C-Si), 71.65 (CH$_2$ crown), 68.39 (THF), 26.56 (THF), 20.92 (^{i}Pr CH$_3$), 20.54 (^{i}Pr CH$_3$), 13.80 (^{i}Pr CH), 5.30 (SiMe$_3$). **^{29}Si{^{1}H} NMR** (δ/ppm, D$_8$-THF): 0.55 (Pn Si^{i}Pr$_3$), −23.63 (SiMe$_3$). **FTIR ($\bar{\nu}$/cm^{-1}):** 3000-2850 (m, b, C–H).

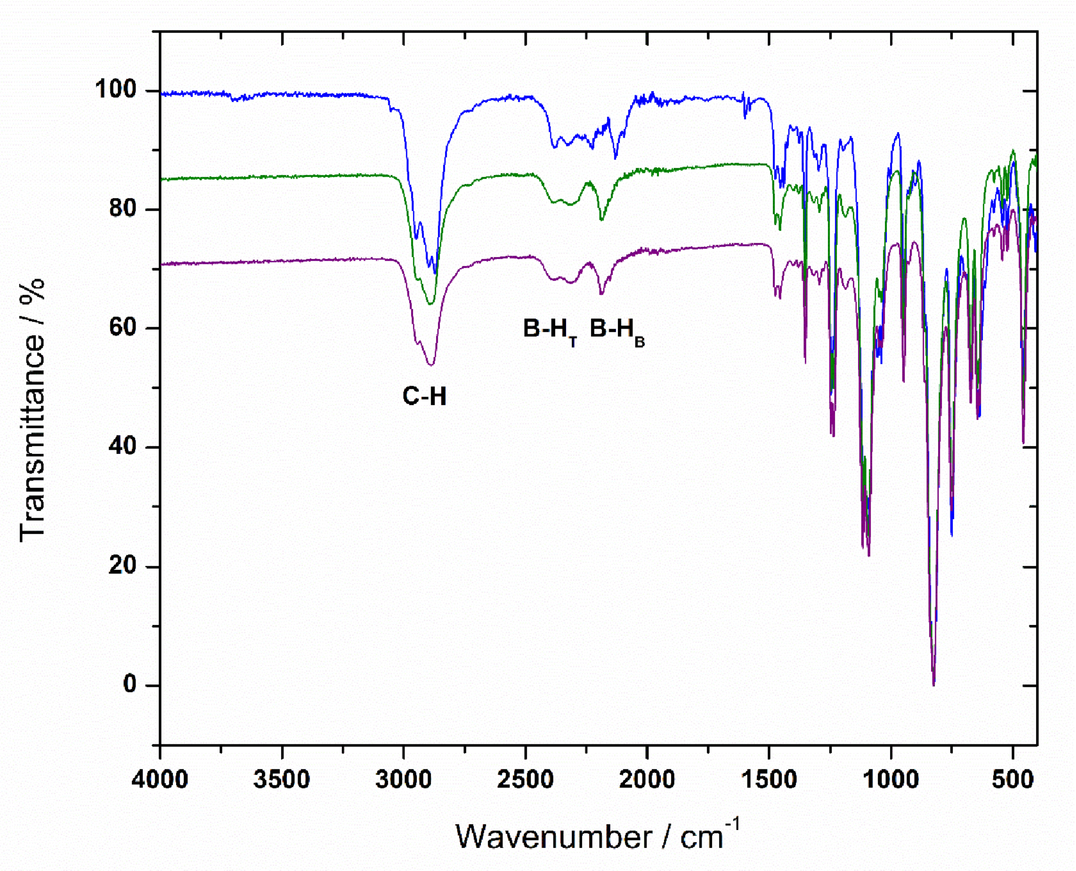

Figure S1. FTIR spectra of [**1**][Na(15-c-5)(THF)$_2$] (blue), [**4**][Na(15-c-5)(THF)$_2$] (green) and **1a** (purple).

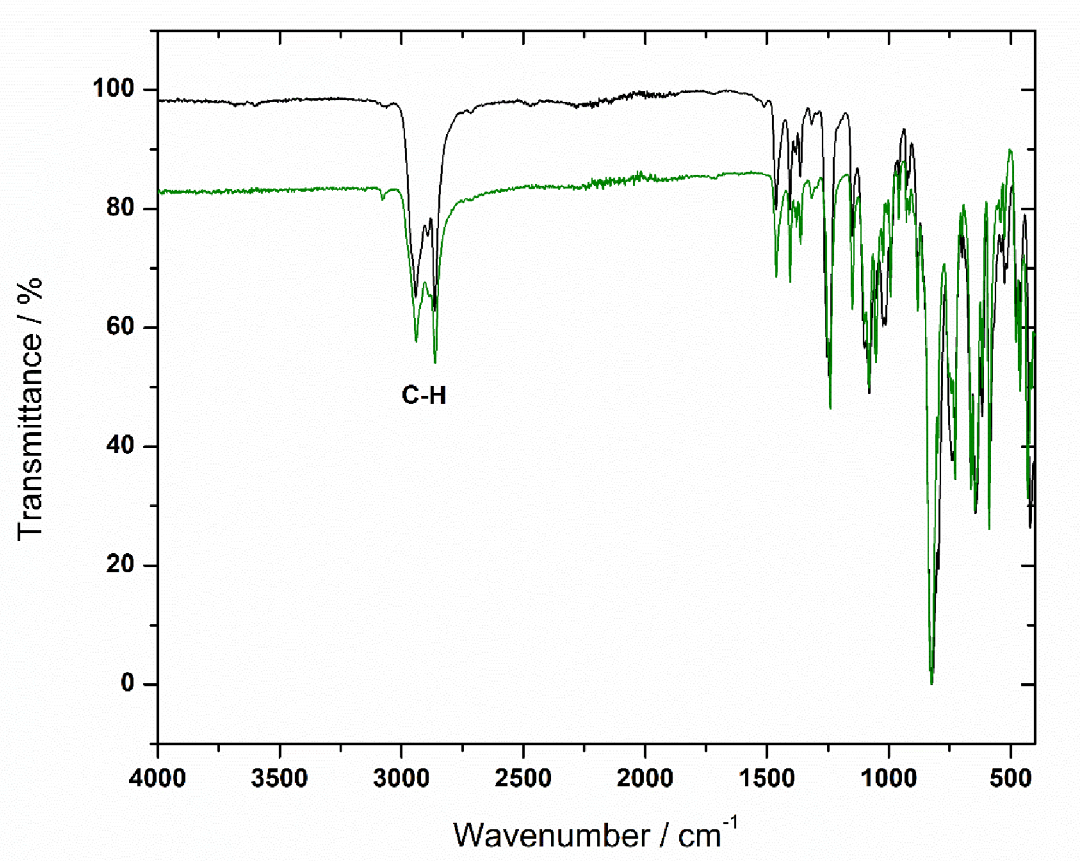

Figure S2. FTIR spectrum of **2** (black) and **5** (green).

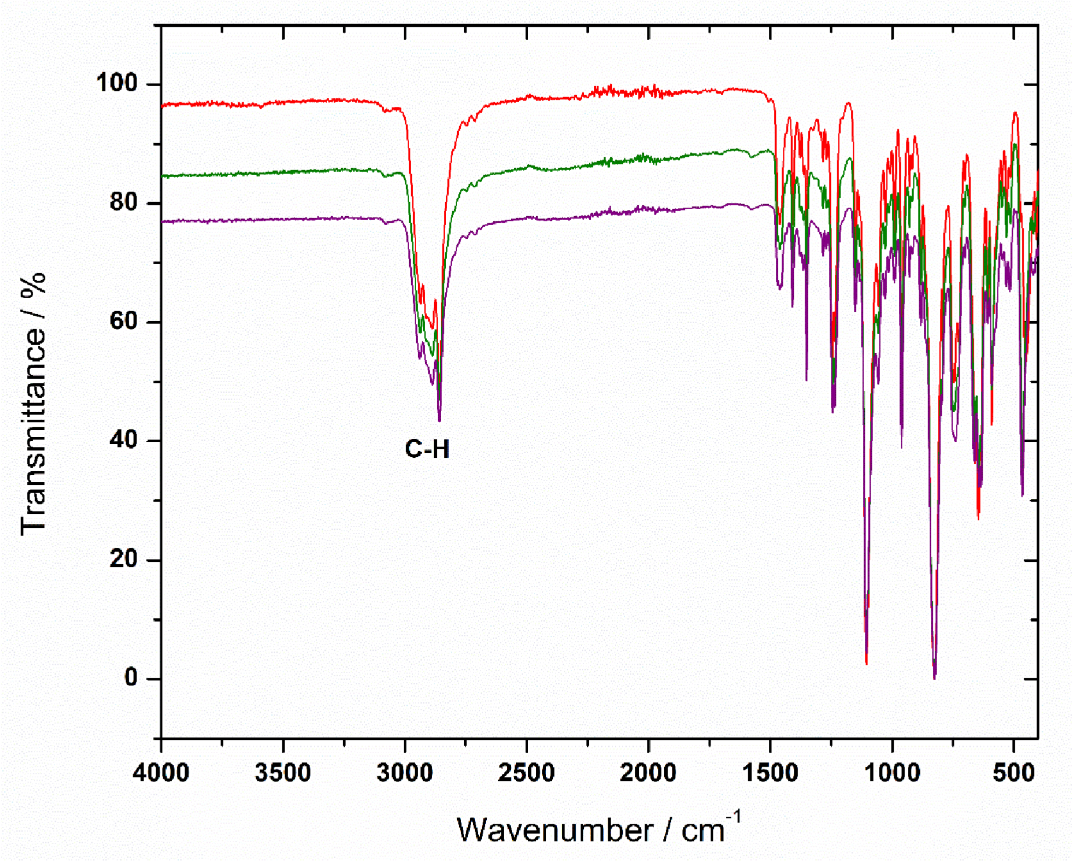

Figure S3. FTIR spectra of [**3**][K(18-c-6)(THF)$_2$] (red), [**6**][K(18-c-6)(THF)$_2$] (green) and **3a** (purple).

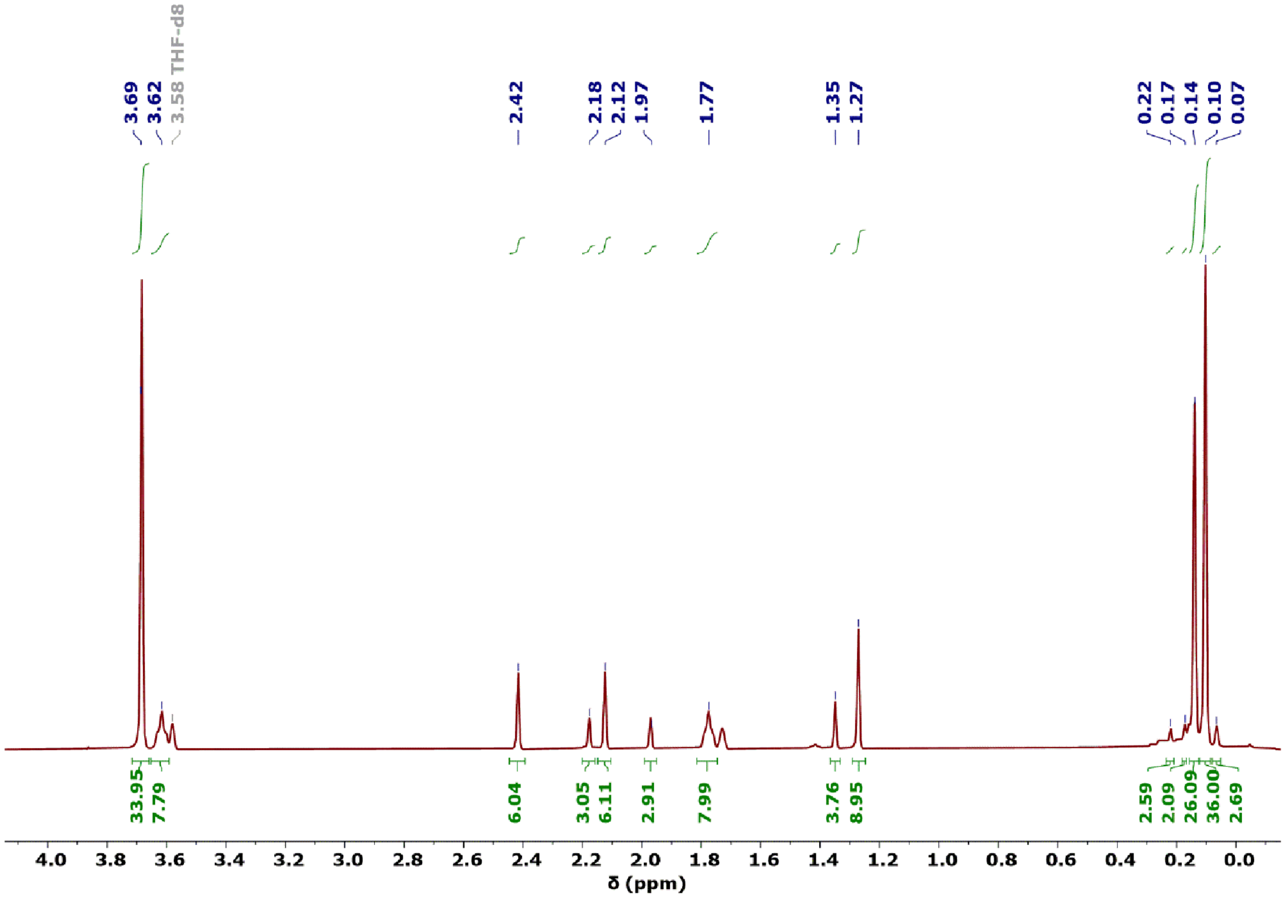

Figure S4. ^{1}H NMR spectrum of [**4**][Na(15-c-5)(THF)$_2$] in D_8-THF.

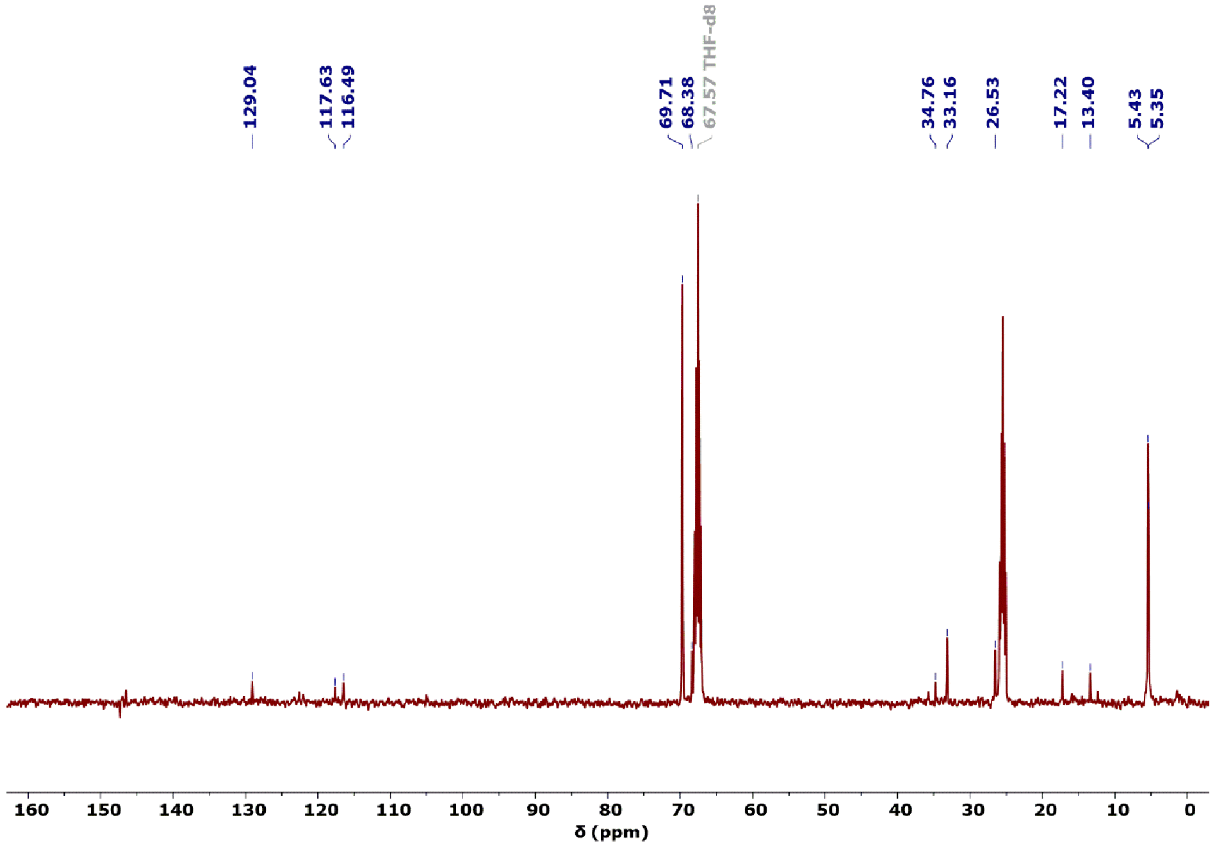

Figure S5. $^{13}C\{^{1}H\}$ NMR spectrum of [**4**][Na(15-c-5)(THF)$_2$] in D_8-THF.

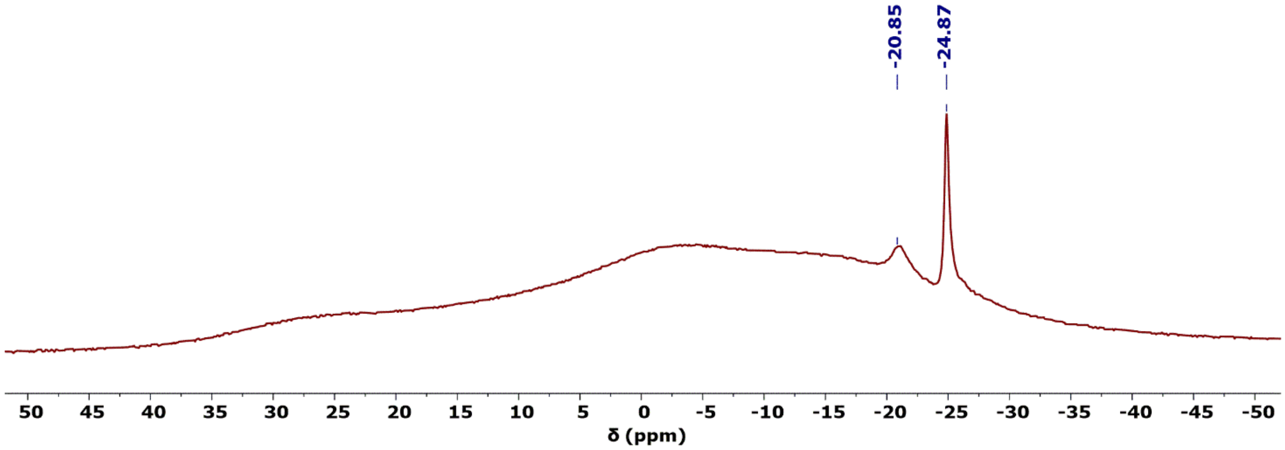

Figure S6. $^{11}B\{^{1}H\}$ NMR spectrum of [**4**][Na(15-c-5)(THF)$_2$] in D_8-THF.

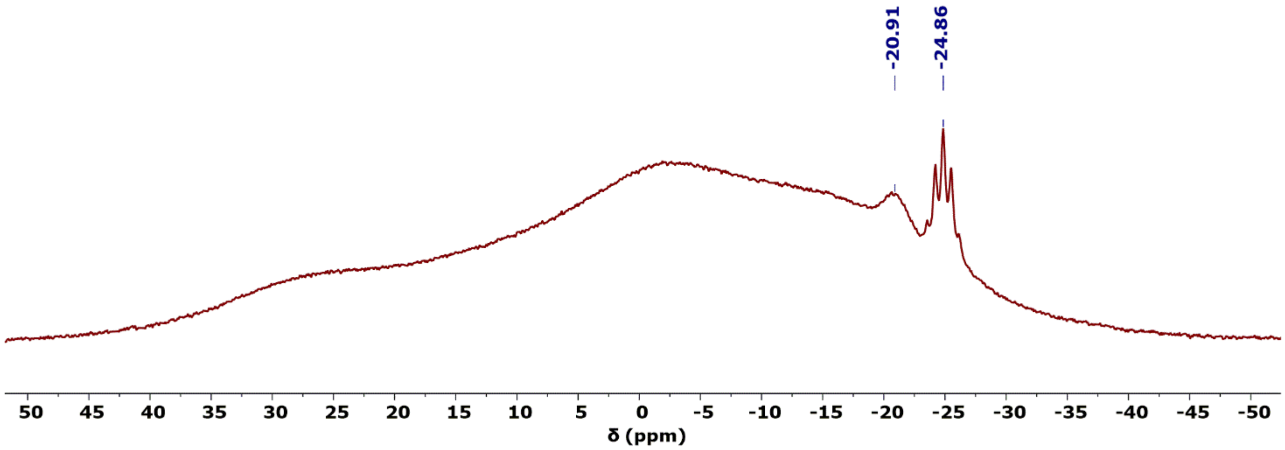

Figure S7. ^{11}B NMR spectrum of [**4**][Na(15-c-5)(THF)$_2$] in D_8-THF.

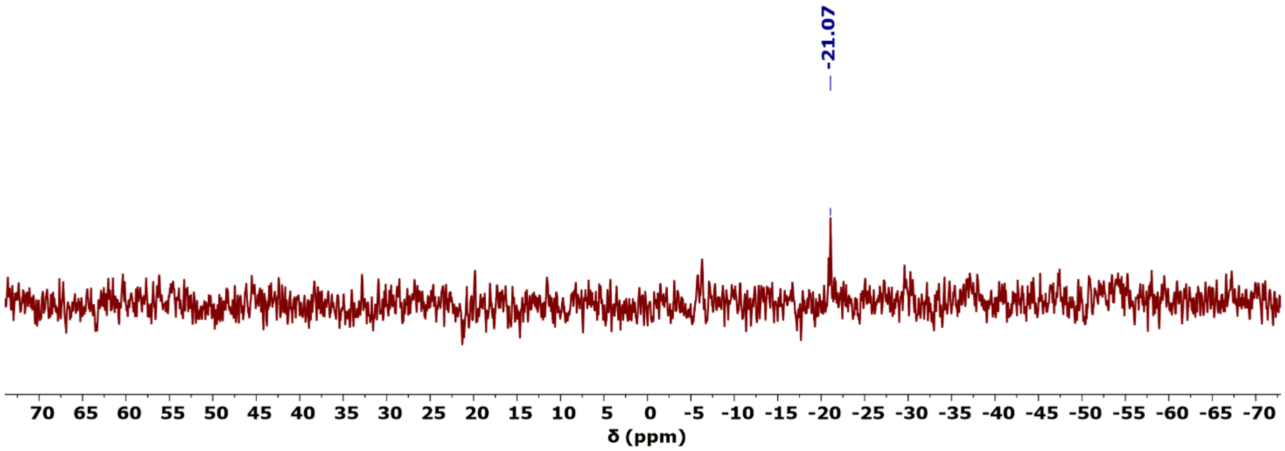

Figure S8. $^{29}Si\{^{1}H\}$ NMR spectrum of [**4**][Na(15-c-5)(THF)$_2$] in D_8-THF.

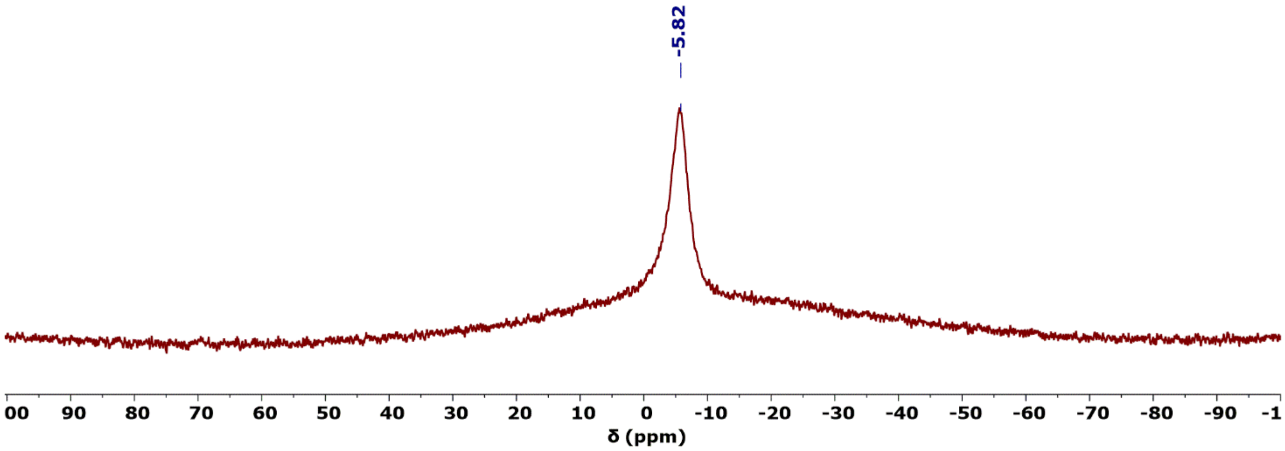

Figure S9. $^{23}Na\{^{1}H\}$ NMR spectrum of [**4**][Na(15-c-5)(THF)$_2$] in D_8-THF.

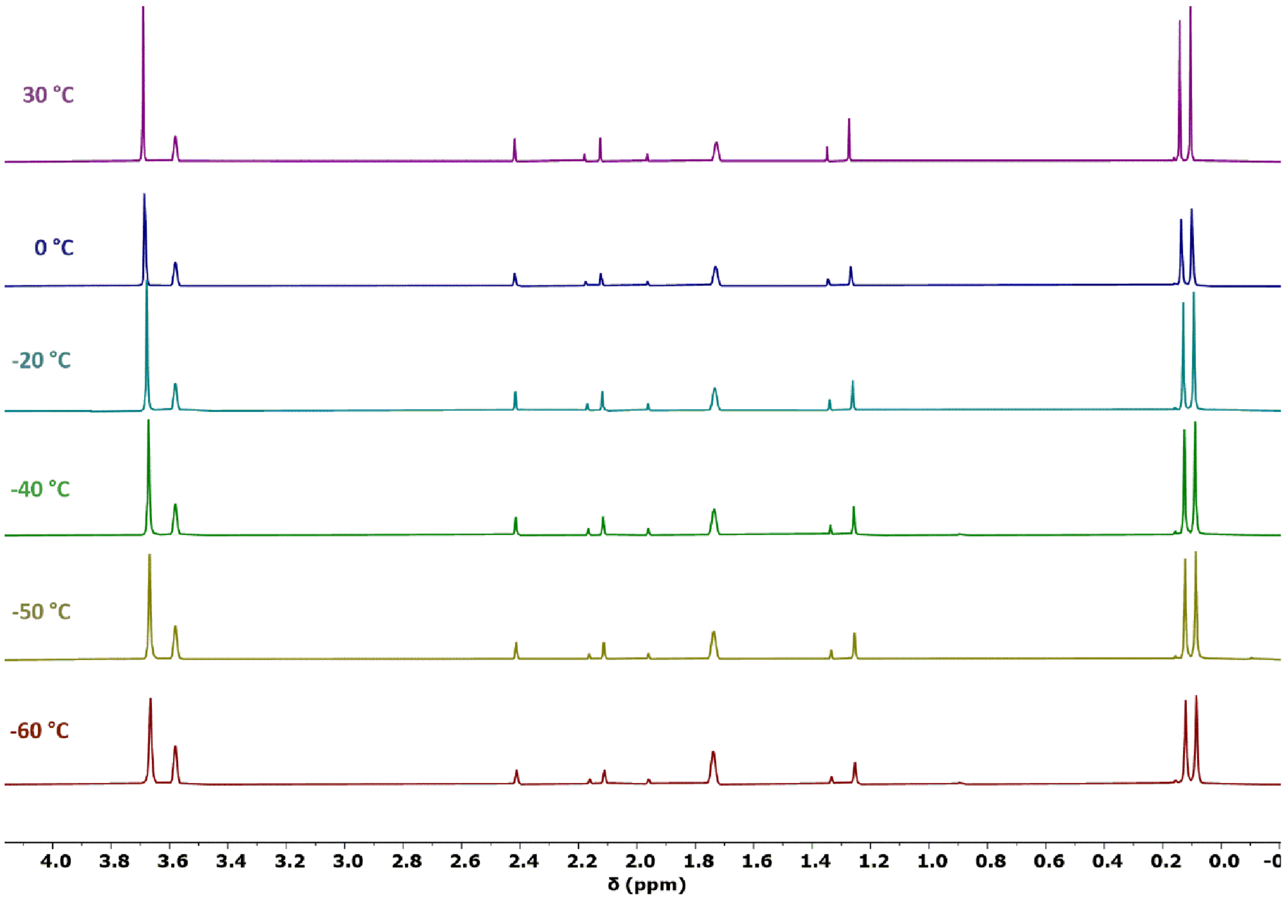

Figure S10 Variable temperature ^{1}H NMR spectra of [**4**][Na(15-c-5)(THF)$_2$] in D_8-THF from +30 to −60 °C.

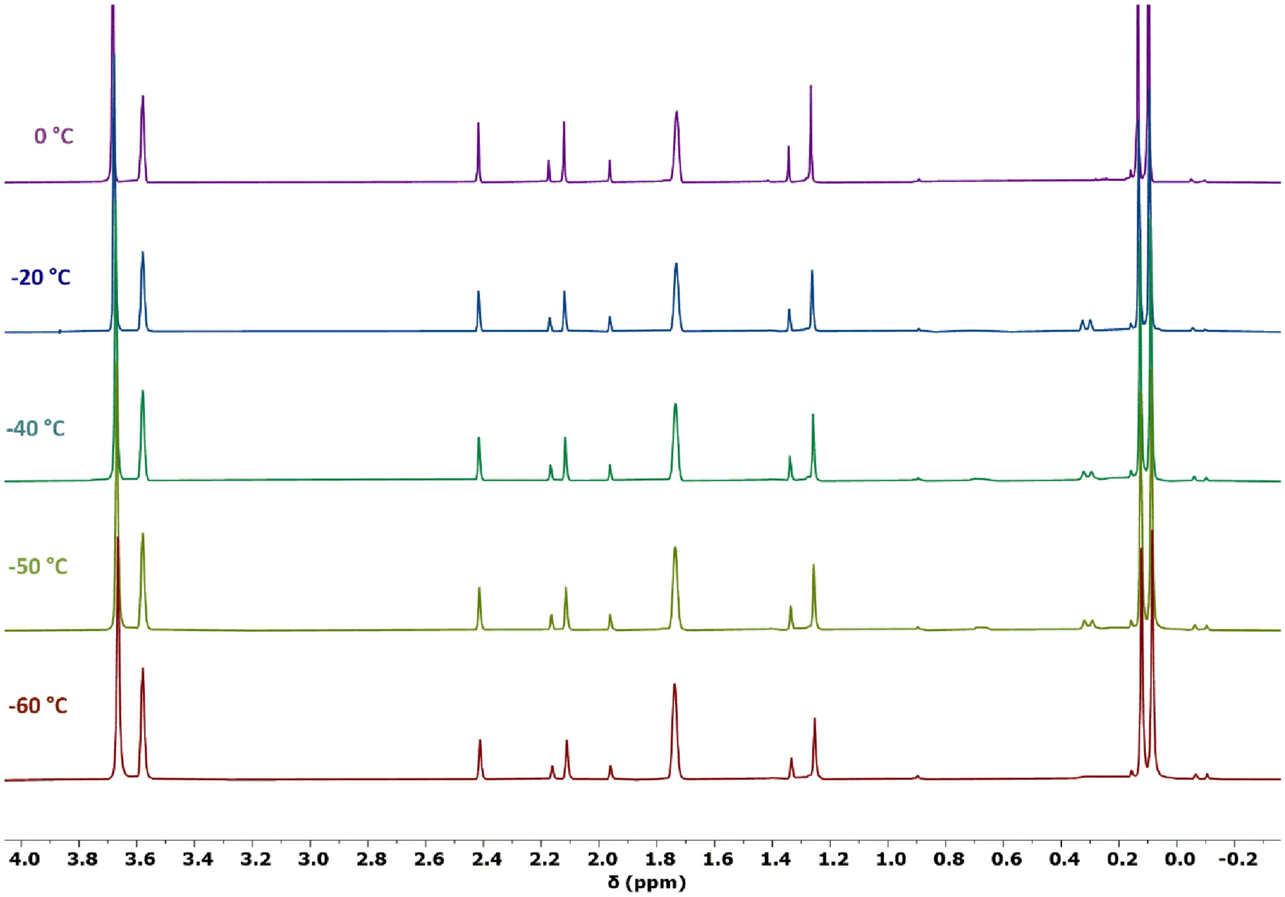

Figure S11. Variable temperature $^{1}H\{^{11}B\}$ NMR spectra of [**4**][Na(15-c-5)(THF)$_2$] in D_8-THF from 0 to −60 °C.

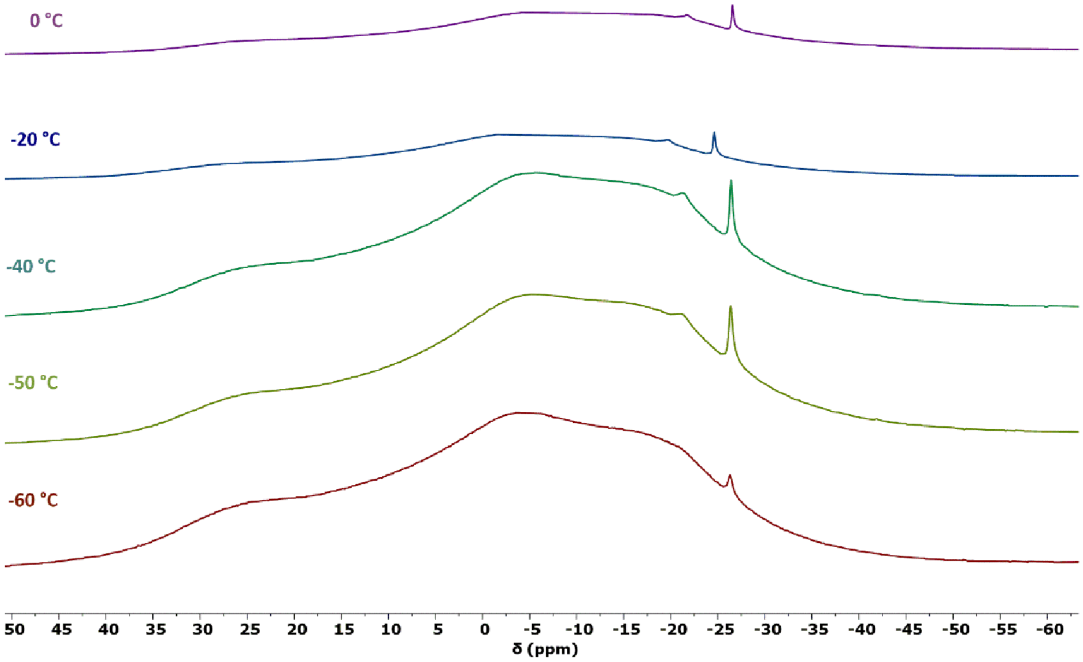

Figure S12. Variable temperature $^{11}B\{^{1}H\}$ NMR spectra of [**4**][Na(15-c-5)(THF)$_2$] in D_8-THF from 0 to −60 °C.

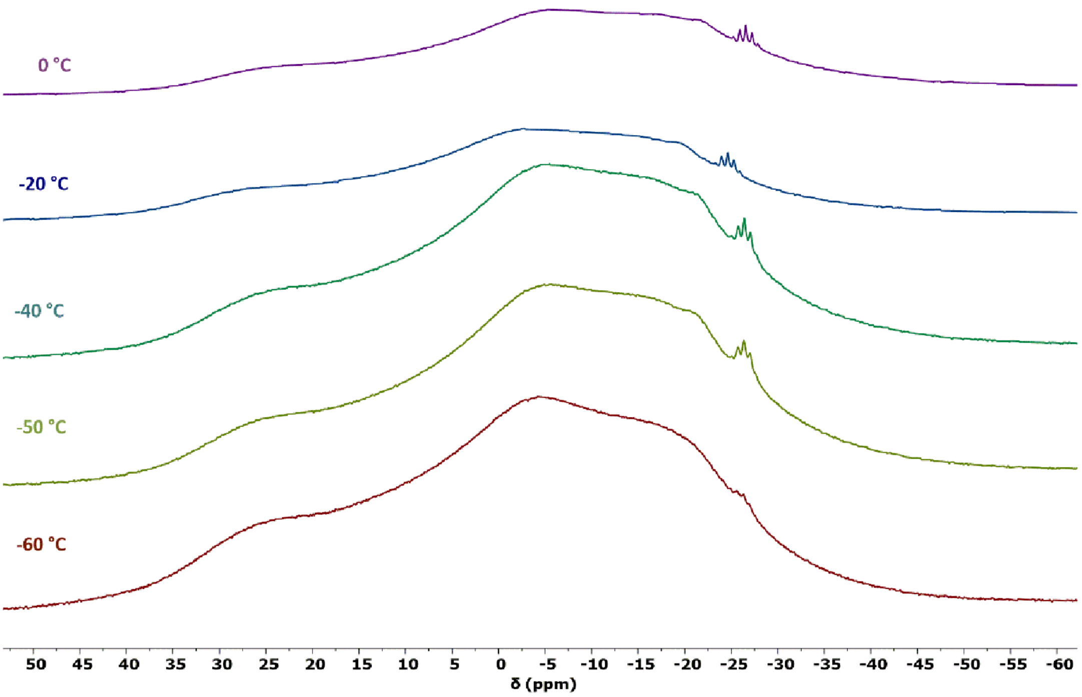

Figure S13. Variable temperature ^{11}B NMR spectra of [**4**][Na(15-c-5)(THF)$_2$] in D_8-THF from 0 to −60 °C.

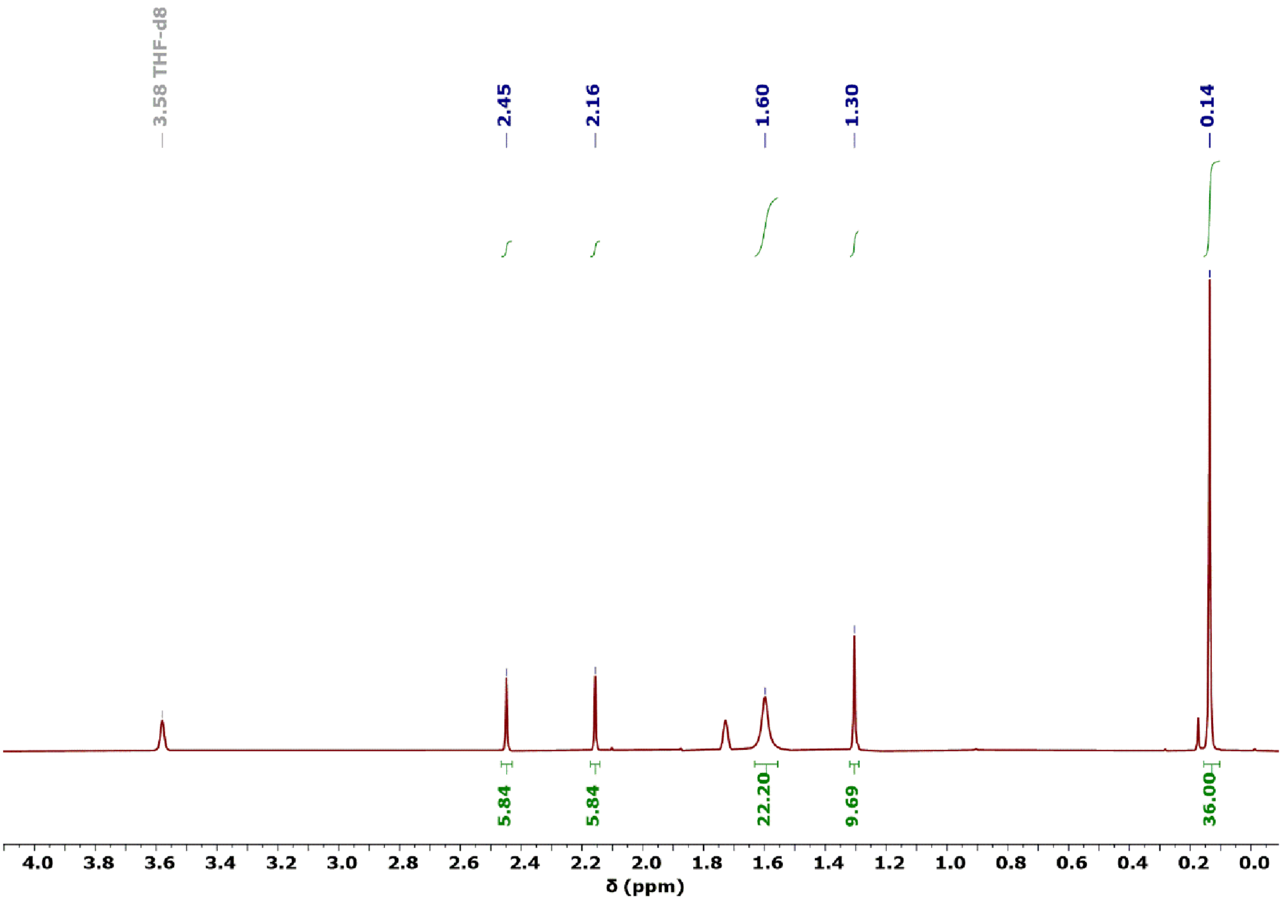

Figure S14. ^{1}H NMR spectrum of **1a** in D_8-THF.

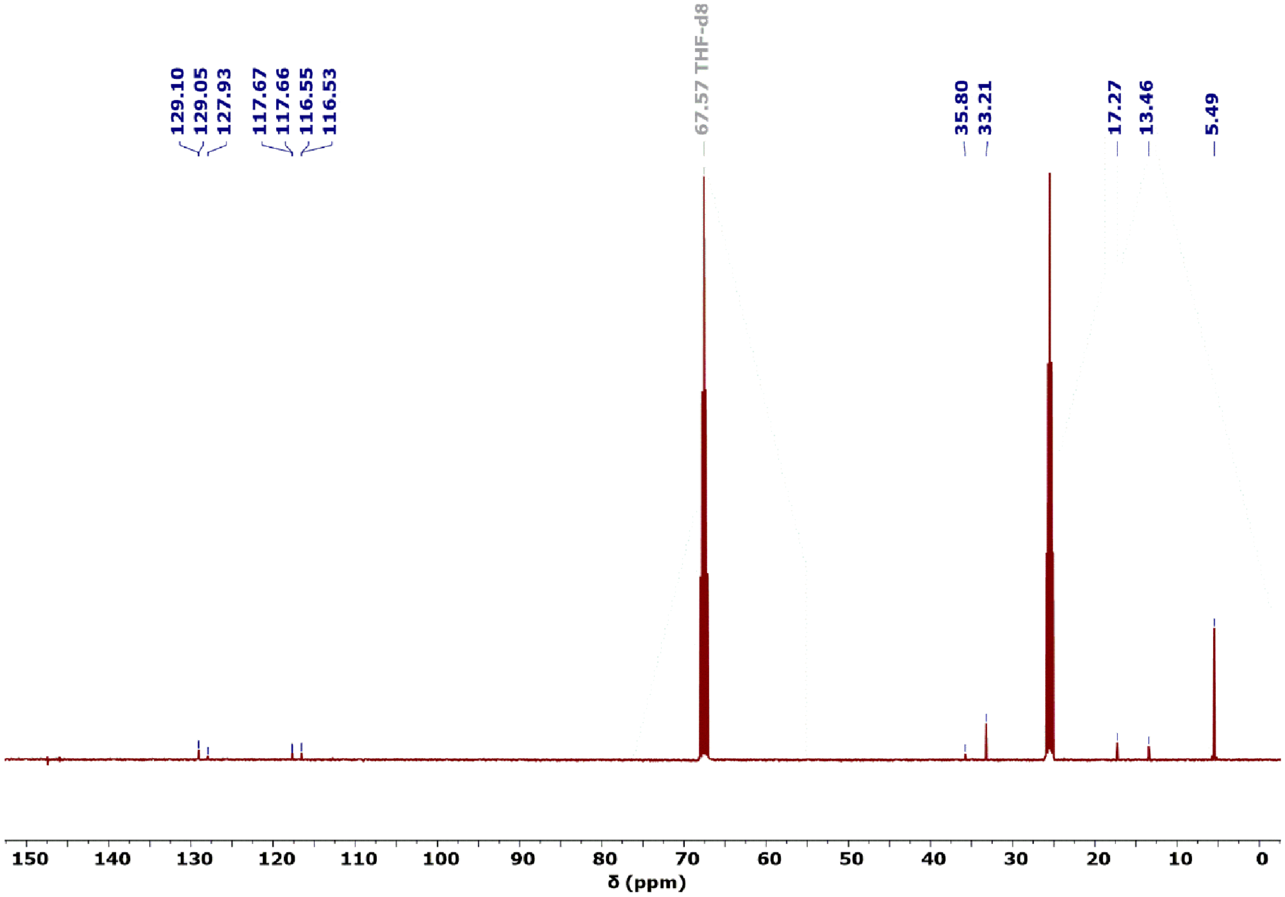

Figure S15. $^{13}C\{^{1}H\}$ NMR spectrum of **1a** in D_8-THF.

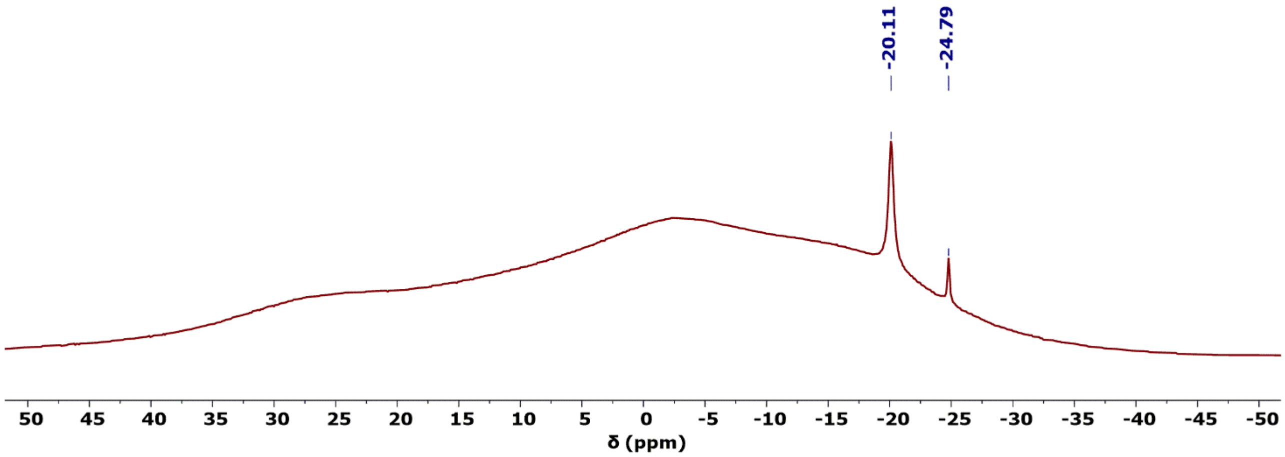

Figure S16. $^{11}B\{^{1}H\}$ NMR spectrum of **1a** in D_8-THF.

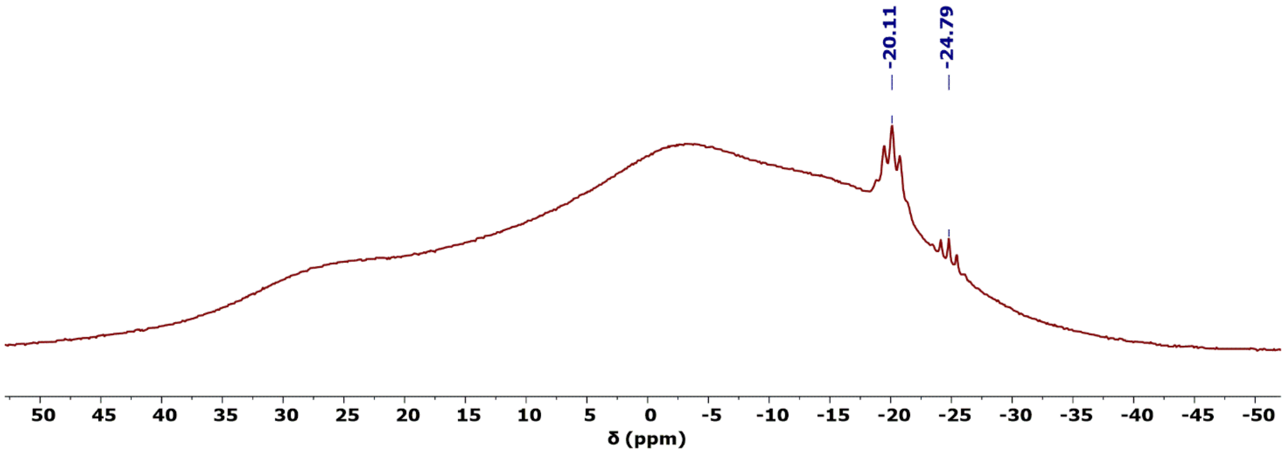

Figure S17. ^{11}B NMR spectrum of **1a** in D_8-THF.

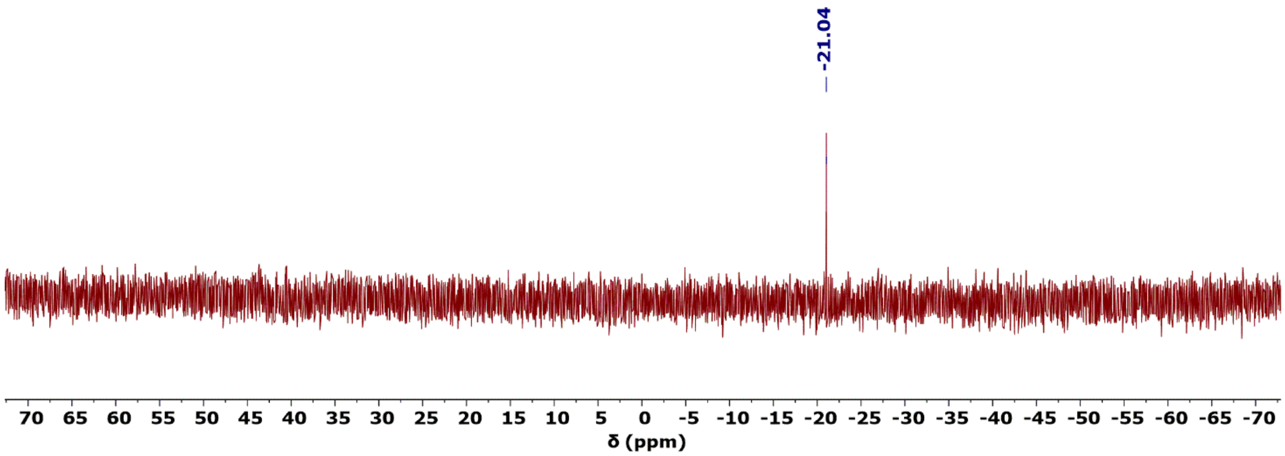

Figure S18. $^{29}Si\{^{1}H\}$ NMR spectrum of **1a** in D_8-THF.

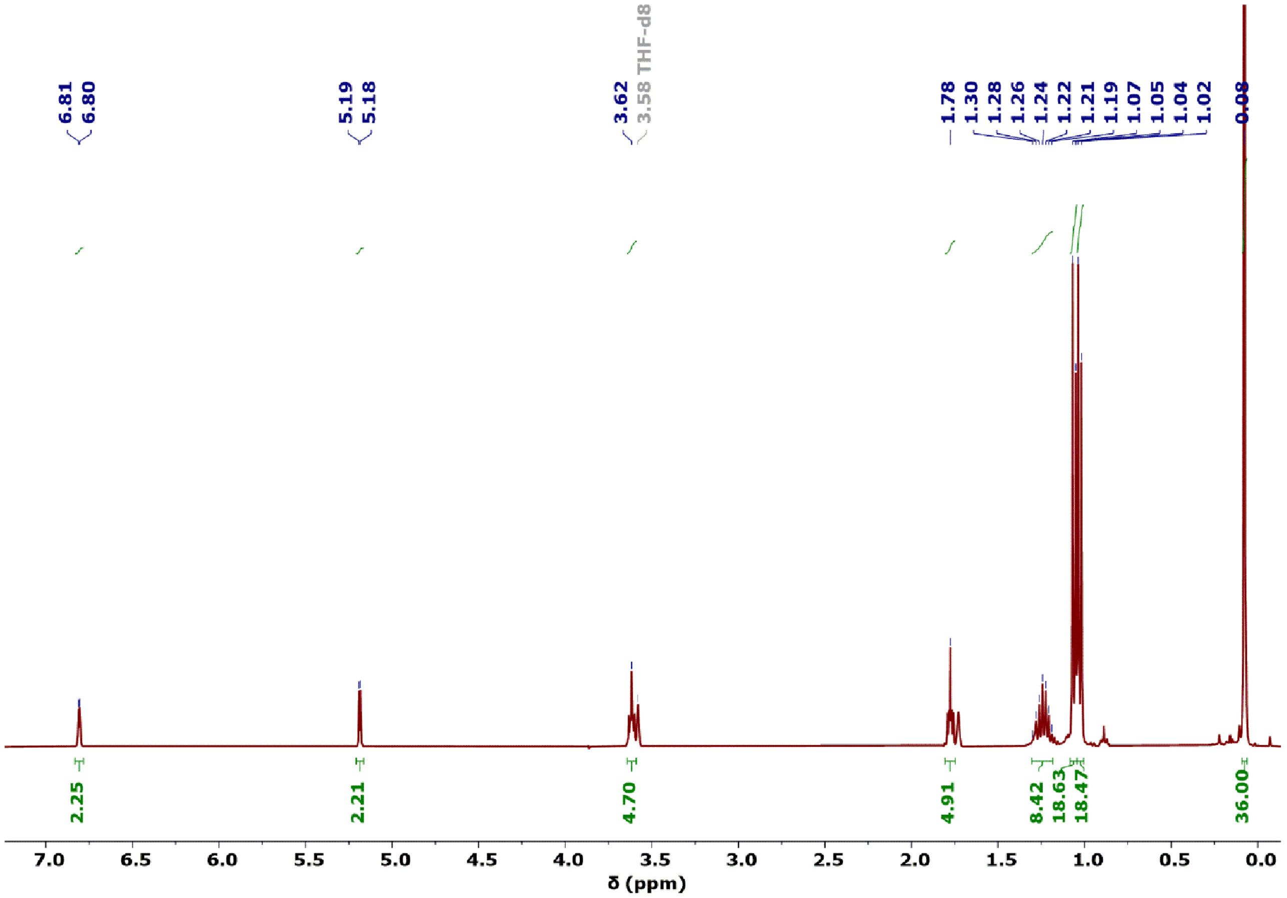

Figure S19. ^{1}H NMR spectrum of **5** in D_8-THF.

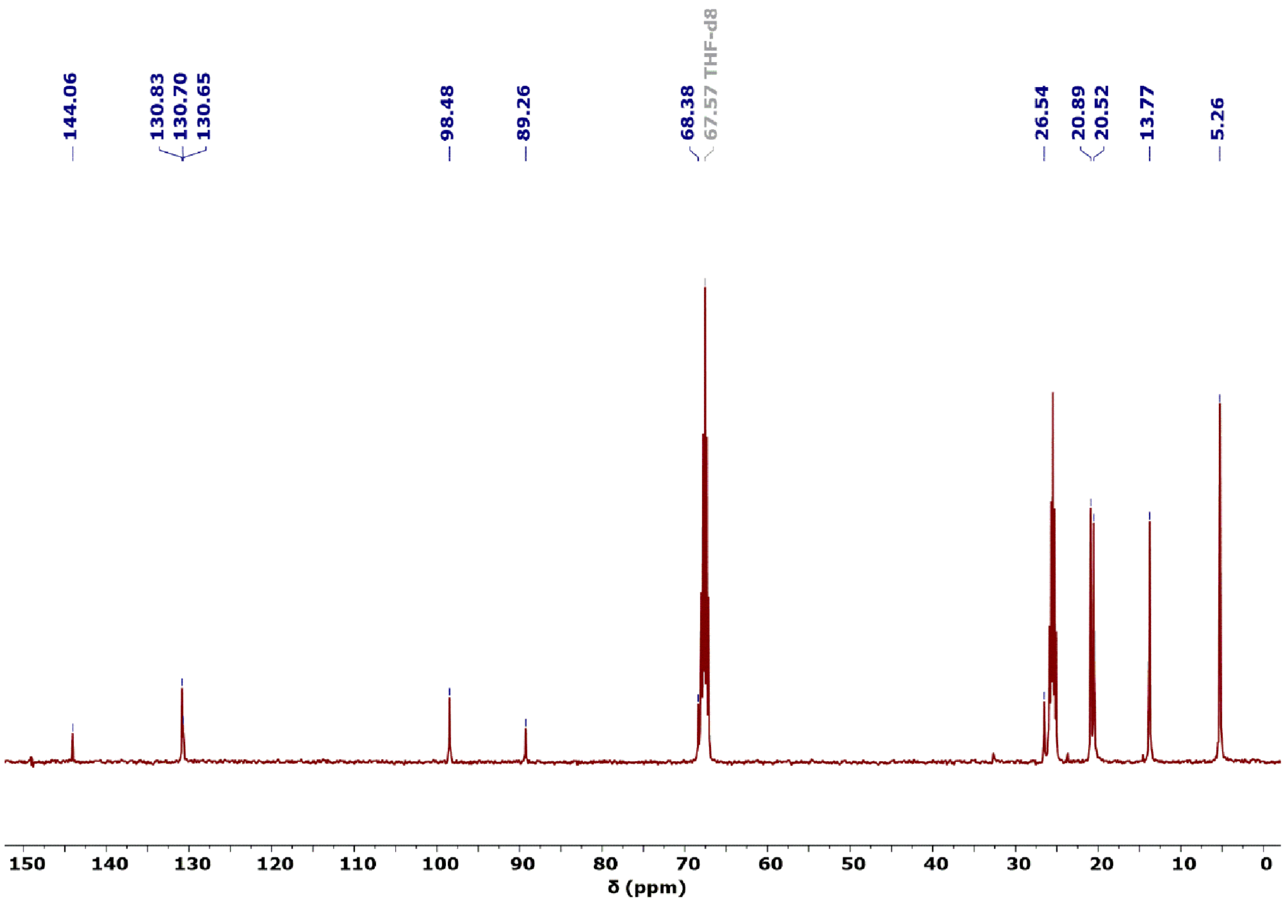

Figure S20. $^{13}C\{^1H\}$ NMR spectrum of **5** in D_8-THF.

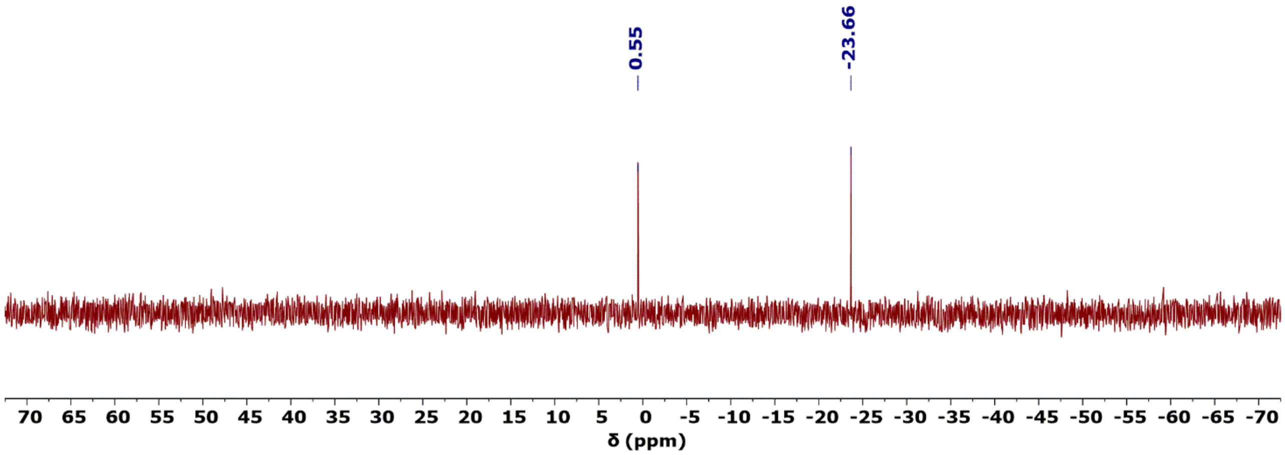

Figure S21. $^{29}Si\{^1H\}$ NMR spectrum of **5** in D_8-THF.

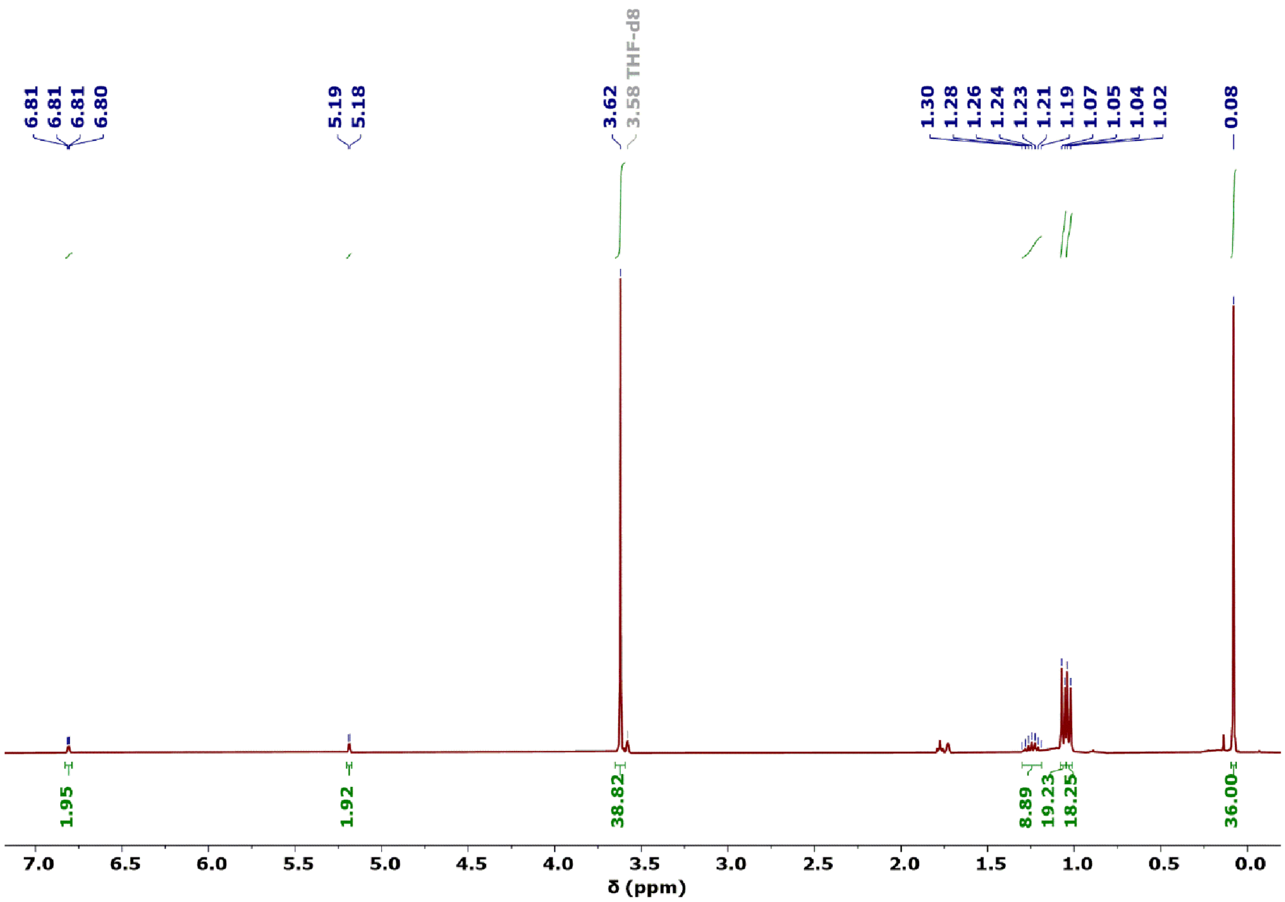

Figure S22. ^{1}H NMR spectrum of [**6**][K(18-c-6)(THF)$_2$] in D_8-THF.

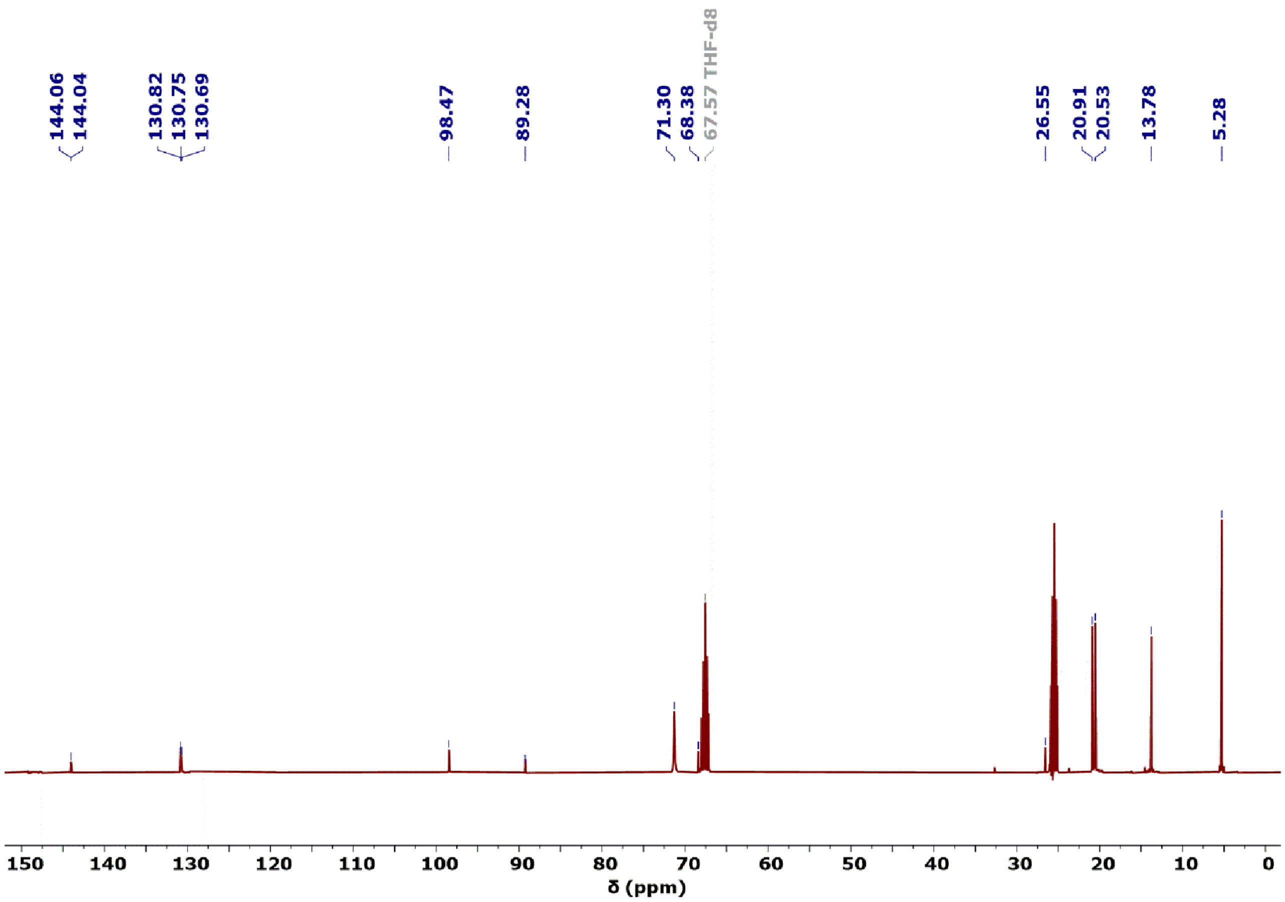

Figure S23. $^{13}C\{^{1}H\}$ NMR spectrum of [**6**][K(18-c-6)(THF)$_2$] in D_8-THF.

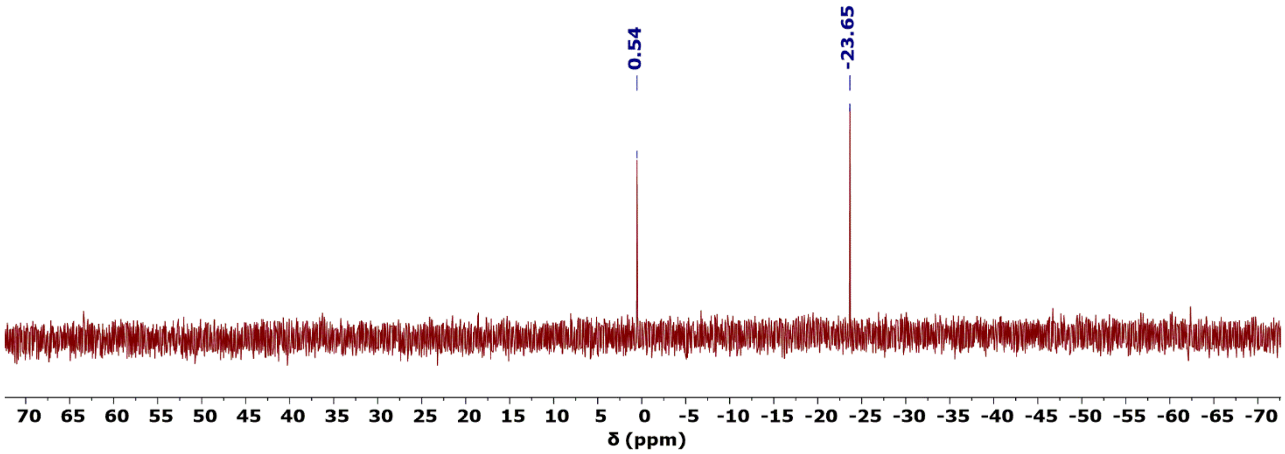

Figure S24. $^{29}Si\{^{1}H\}$ NMR spectrum of [**6**][K(18-c-6)(THF)$_2$] in D_8-THF.

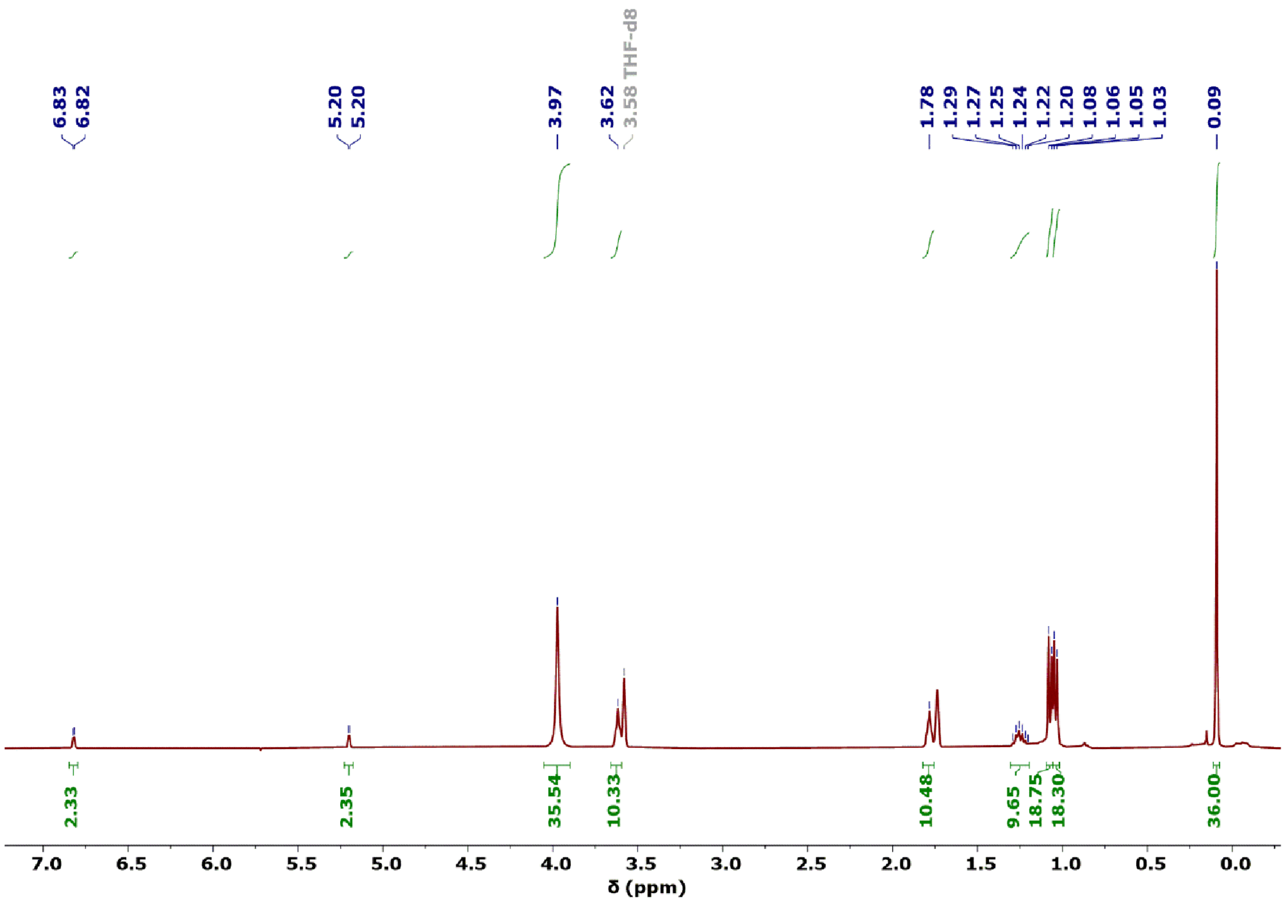

Figure S25. ^{1}H NMR spectrum of **3a** in D_8-THF.

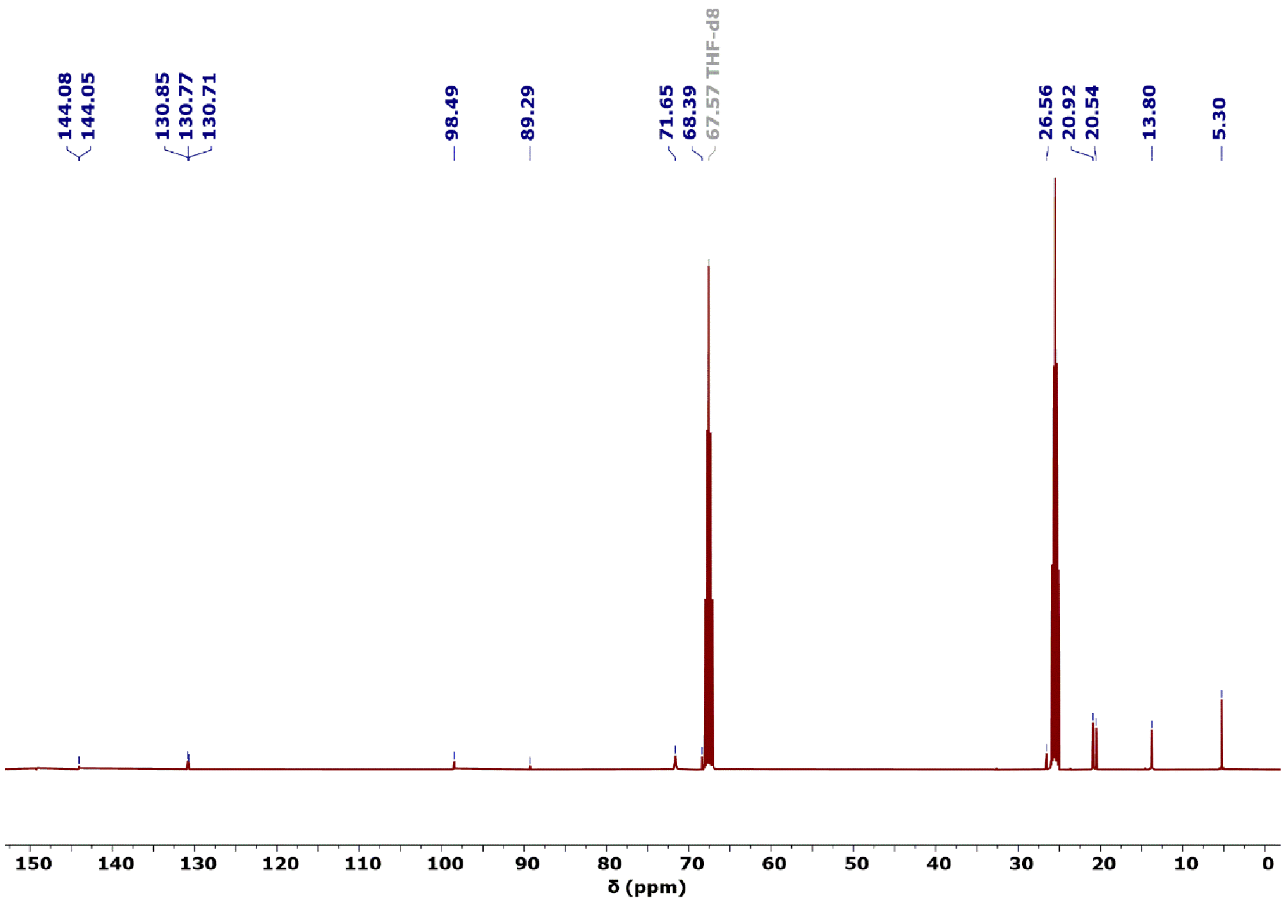

Figure S26. $^{13}C\{^1H\}$ NMR spectrum of **3a** in D_8-THF.

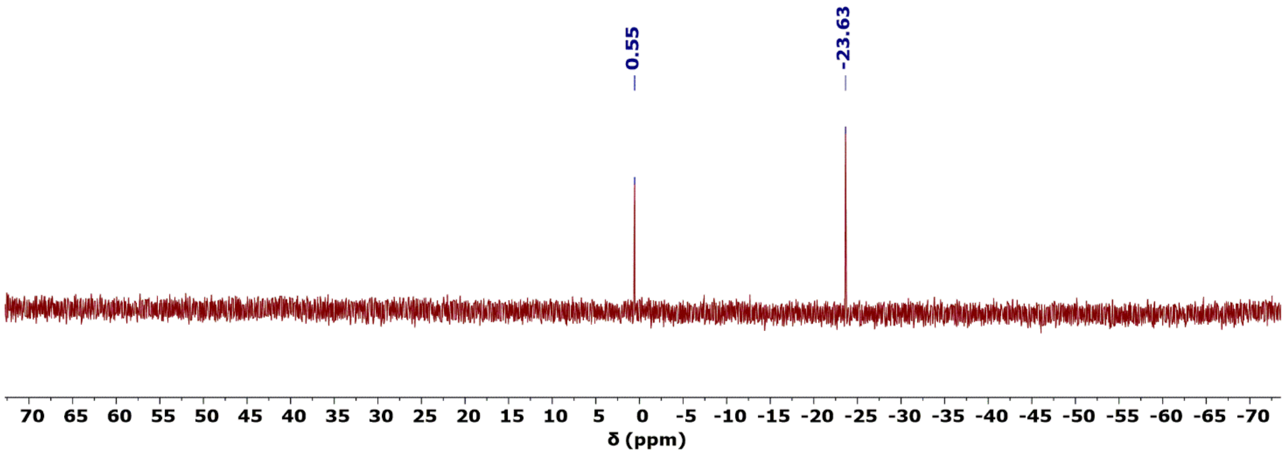

Figure S27. $^{29}Si\{^1H\}$ NMR spectrum of **3a** in D_8-THF.

X-ray Crystallography

Measurements on **1**-**4** and **6** were carried out on an Agilent Gemini Ultra diffractometer using CuKα radiation (λ = 1.54184 Å) at 100 K. Data for **5** were measured on a Bruker D8 Venture diffractometer using GaKα radiation (λ = 1.3418 Å) at 95 K. Structures were solved in Olex2 with SHELXT[4–6] using intrinsic phasing and were refined with SHELXL using least squares minimisation. Anisotropic thermal parameters were used for the non-hydrogen atoms and isotropic parameters for the hydrogen atoms. Hydrogen atoms on carbons were added geometrically and refined using a riding model. For [**1**][Na(15-c-5)(THF)$_2$], the hydrogen atoms from the borohydride were located based on residual electron density and were refined freely. In the case of **2**, the structure was found to be disordered over a mirror plane and so was modelled free from special position constraints with 50 % occupancy. For [**6**][K(18-c-6)(THF)$_2$], extremely weak high-angle diffraction prevented the collection of publishable-quality data, however the atom connectivity was established.

Table S1. Crystal data and structure refinement for all compounds.

	**[1] [Na(15-c-5)(THF)$_2$]**	**2**	**[3] [K(18-c-6)(THF)$_2$]**	**[4] [Na(15-c-5)(THF)$_2$]**	**5**	**[6] [K(18-c-6)(THF)$_2$]**
CCDC ref. code	2058819	2058818	2058817	2133570	2133571	2133572
formula	$C_{47}H_{97}DyNaBO_7Si_4$	$C_{46}H_{90}DyKOSi_6$	$C_{70}H_{138}DyKO_{10}Si_6$	$C_{47}H_{97}YNaBO_7Si_4$	$C_{50}H_{98}YKO_2Si_6$	$C_{70}H_{138}YKO_{10}Si_6$
formula weight	1082.90	1029.31	570.60	1009.31	1024.32	1434.33
crystal system	monoclinic	monoclinic	triclinic	monoclinic	monoclinic	triclinic
space group	$P2_1/c$	$P2_1/m$	P–1	$P2_1/c$	$C2/c$	P–1
a (Å)	16.5631(2)	10.3825(2)	13.5715(7)	16.4042(2)	13.357(3)	13.5082(9)
b (Å)	14.5735(2)	21.3004(4)	14.7132(6)	14.6279(2)	20.678(3)	14.7014(11)
c (Å)	23.7561(3)	12.4091(3)	24.4618(12)	23.8840(2)	21.454(3)	24.4627(11)
α (°)	90	90	74.369(4)	90	90	98.438(5)
β (°)	98.7160(10)	91.536(2)	81.883(4)	98.4630(10)	90.104(8)	97.850(4)
γ (°)	90	90	62.547(5)	90	90	117.440(7)
V (Å^3)	5668.08(13)	2743.30(10)	4173.4(4)	5668.77(12)	5925.5(16)	4169.9(5)
Z	4	2	2	4	4	2
T (K)	100(1)	99.8(8)	100(1)	100(1)	95.0	100
ρ_{calc} (g/cm^3)	1.269	1.246	1.202	1.183	1.148	1.142
F (000)	2292.0	1086.0	1614.0	2184.0	2216.0	1556.0
Reflections collected	30974	17150	37150	79006	39885	69645
Independent reflections	10384	5157	14665	10976	6004	14952
R_{int} (%)	4.87	3.58	5.13	4.89	3.59	21.84
GOF on F^2	1.178	1.054	0.953	1.140	1.085	1.224
$R_1{}^a$	0.0690	0.0561	0.0539	0.0551	0.0571	0.1596
$R_w{}^b$	0.1501	0.1575	0.1386	0.1162	0.1544	0.4829

^{a} $R_1[I>2\sigma(I)] = \sum||F_o|-|F_c||/\sum|F_o|$; ^{b} $R_w[\text{all data}] = [\sum\{w(F_o{}^2-F_c{}^2)^2\}/\sum\{w(F_o{}^2)^2\}]^{1/2}$

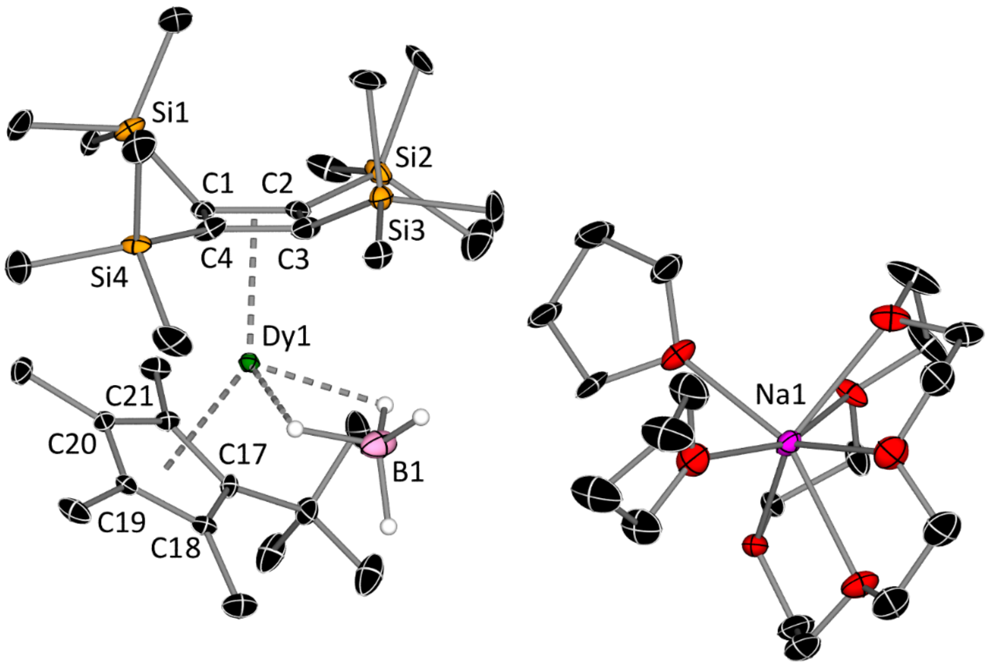

Figure S28. Thermal ellipsoid representations (30% probability) of the molecular structure of [**1**][Na(15-crown-5)(THF)$_2$]. H = white, B = light pink, C = black, O = red, Na = pink, Si = orange, Dy = green. For clarity, disordered components have been omitted and only the hydrogen atoms bound to boron are shown.

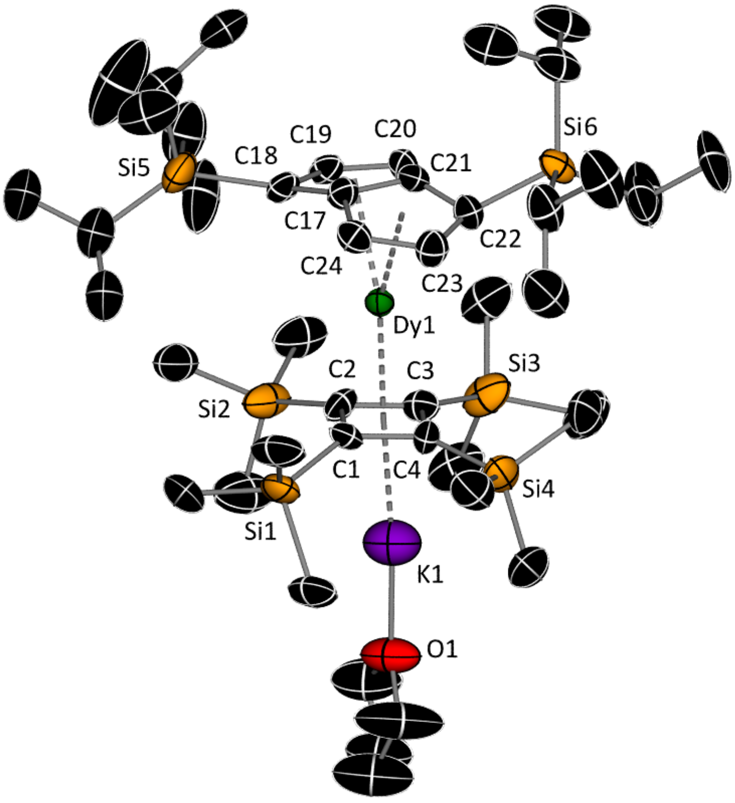

Figure S29. Thermal ellipsoid representations (30% probability) of the molecular structure of **2**. C = black, O = red, K = purple, Si = orange, Dy = green. For clarity, hydrogen atoms have been omitted.

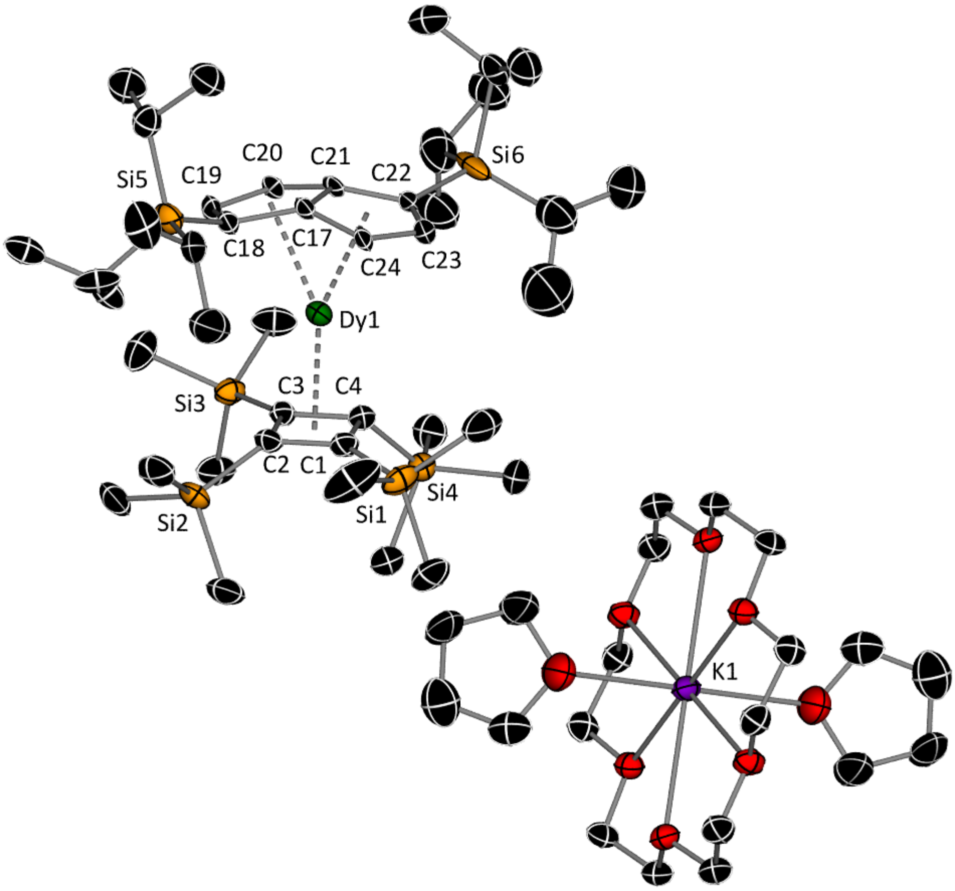

Figure S30. Thermal ellipsoid representations (30% probability) of the molecular structure of [**3**][K(18-crown-6)(THF)$_2$]. C = black, O = red, K = purple, Si = orange, Dy = green. For clarity, disordered components, two THF molecules in the lattice and hydrogen atoms have been omitted.

Table S2. Selected bond lengths and angles for [**1**][Na(15-crown-5)(THF)$_2$], **2** and [**3**][K(18-crown-6)(THF)$_2$].

	**[1][Na(15-crown-5)(THF)$_2$]**†	**2**	**[3][K(18-crown-6)(THF)$_2$]**†
Dy–C(η^4-Cb) (Å)	Dy1–C1: 2.486(6)<br>Dy1–C2: 2.513(7)<br>Dy1–C3: 2.523(6)<br>Dy1–C4: 2.482(7)	Dy1–C1: 2.49(2)<br>Dy1–C2: 2.47(3)<br>Dy1–C3: 2.56(3)<br>Dy1–C4: 2.57(3)	Dy1–C1: 2.514(5)<br>Dy1–C2: 2.580(5)<br>Dy1–C3: 2.526(5)<br>Dy1–C4: 2.469(5)
Dy–(η^4-Cb$_{cent}$) (Å)	2.2728(4)	2.306(11)	2.294(3)
Dy–C(η^5-Cp) (Å)	Dy1–C17: 2.688(6)<br>Dy1–C18: 2.700(6)<br>Dy1–C19: 2.691(6)<br>Dy1–C20: 2.681(7)<br>Dy1–C21: 2.667(6)	N/A	N/A
Dy–(η^5-Cp$_{cent}$) (Å)	2.3975(3)	N/A	N/A
Dy–C(η^8-Pn) (Å)	N/A	Dy1–C17: 2.385(8)<br>Dy1–C18: 2.712(10)<br>Dy1–C19: 2.782(10)<br>Dy1–C20: 2.642(10)<br>Dy1–C21: 2.400(8)<br>Dy1–C22: 2.683(9)<br>Dy1–C23: 2.831(8)<br>Dy1–C24: 2.661(7)	Dy1–C17: 2.413(10)<br>Dy1–C18: 2.698(10)<br>Dy1–C19: 2.825(14)<br>Dy1–C20: 2.651(16)<br>Dy1–C21: 2.405(8)<br>Dy1–C22: 2.698(7)<br>Dy1–C23: 2.814(9)<br>Dy1–C24: 2.644(11)
Dy–(η^8-Pn$_{cent}$) (Å)	N/A	2.282(5)<br>2.300(4)	2.301(6)<br>2.296(4)
K–C(η^4-Cb$_{cent}$) (Å)	N/A	2.371(12)	N/A
(η^4-Cb$_{cent}$)–Dy–(η^5-Cp$_{cent}$) (°)	141.52(2)	N/A	N/A
(η^4-Cb$_{cent}$)–Dy–(η^8-Pn$_{cent}$) (°)	N/A	156.4(3)<br>154.2(3)	152.89(12)<br>157.46(17)

† From the highest occupancy component of the crystal structure.

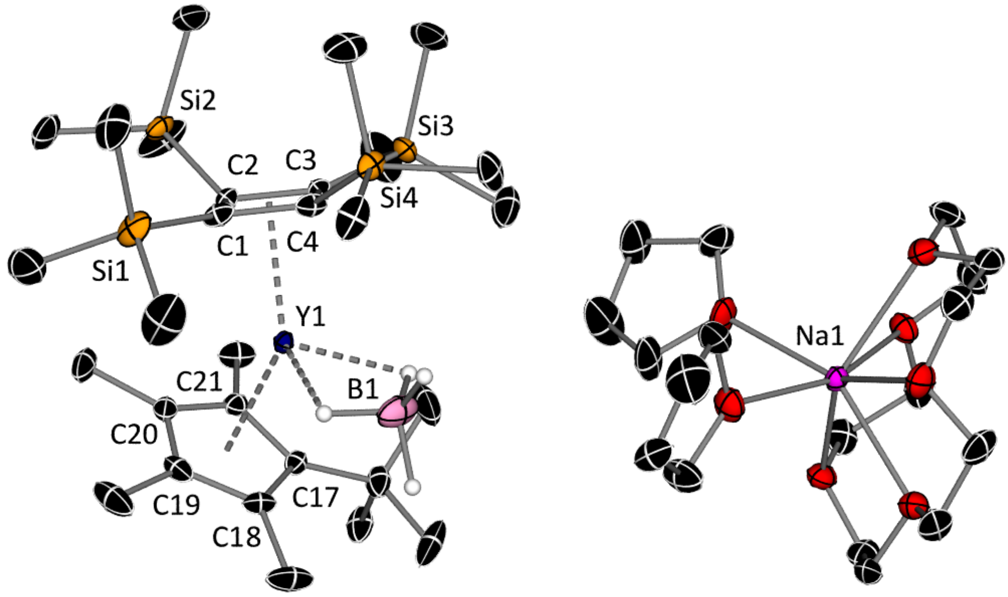

Figure S31. Thermal ellipsoid representations (30% probability) of the molecular structure of [**3**][Na(15-crown-5)(THF)$_2$]. H = white, B = light pink, C = black, O = red, Na = pink, Si = orange, Y = blue. For clarity, disordered components have been omitted and only the hydrogen atoms bound to boron are shown.

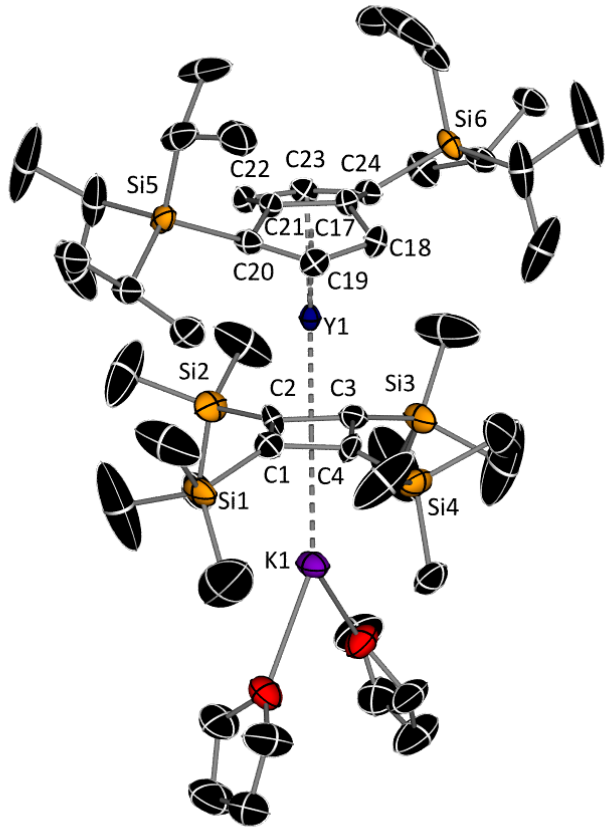

Figure S32. Thermal ellipsoid representations (30% probability) of the molecular structure of **5**. C = black, O = red, K = purple, Si = orange, Y = blue. For clarity, disordered components and hydrogen atoms have been omitted.

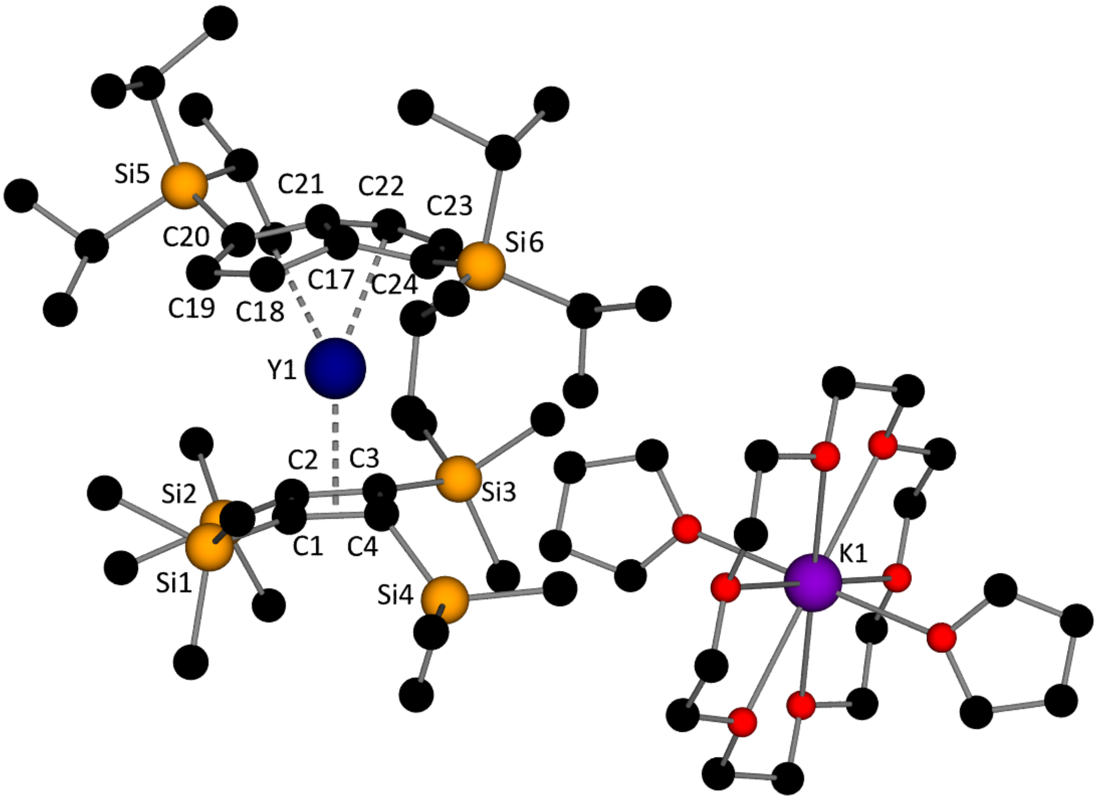

Figure S33. Ball and stick representation of the molecular structure of [**6**][K(18-crown-6)(THF)$_2$]. C = black, O = red, K = purple, Si = orange, Y = blue. For clarity, hydrogen atoms have been omitted.

Table S3. Selected bond lengths and angles for [**4**][Na(15-crown-5)(THF)$_2$], **5** and [**6**][K(16-crown-6)(THF)$_2$].

	[**4**][Na(15-crown-5)(THF)$_2$]	**5**†	[**6**][K(16-crown-6)(THF)$_2$]
Y–C(η^4-Cb) (Å)	Y1–C1: 2.493(3)<br>Y1–C2: 2.485(3)<br>Y1–C3: 2.503(3)<br>Y1–C4: 2.526(3)	Y1–C1: 2.55(2)<br>Y1–C2: 2.55(2)<br>Y1–C3: 2.55(2)<br>Y1–C4: 2.55(2)	Y1–C1: 2.508(9)<br>Y1–C2: 2.477(11)<br>Y1–C3: 2.521(10)<br>Y1–C4: 2.56(1)
Y–(η^4-Cb$_{cent}$) (Å)	2.2696(3)	2.3137(5)	2.2927(8)
Y–C(η^5-Cp) (Å)	Y1–C17: 2.696(3)<br>Y1–C18: 2.702(4)<br>Y1–C19: 2.680(4)<br>Y1–C20: 2.660(3)<br>Y1–C21: 2.659(3)	N/A	N/A
Y–(η^5-Cp$_{cent}$) (Å)	2.3936(3)	N/A	N/A
Y–C(η^8-Pn) (Å)	N/A	Y1–C17: 2.392(6)<br>Y1–C18: 2.63(2)<br>Y1–C19: 2.791(6)<br>Y1–C20: 2.705(7)<br>Y1–C21: 2.392(6)<br>Y1–C22: 2.63(2)<br>Y1–C23: 2.791(6)<br>Y1–C24: 2.705(7)	Y1–C17: 2.396(8)<br>Y1–C18: 2.679(12)<br>Y1–C19: 2.682(13)<br>Y1–C20: 2.671(11)<br>Y1–C21: 2.3965(8)<br>Y1–C22: 2.6365(8)<br>Y1–C23: 2.7736(9)<br>Y1–C24: 2.6341(11)
Y–(η^8-Pn$_{cent}$) (Å)	N/A	2.2827(5)<br>2.2827(5)	2.2703(9)<br>2.2688(8)
K–C(η^4-Cb$_{cent}$) (Å)	N/A	2.7167(11)	N/A
(η^4-Cb$_{cent}$)–Y–(η^5-Cp$_{cent}$) (°)	140.686(12)	N/A	N/A
(η^4-Cb$_{cent}$)–Y–(η^8-Pn$_{cent}$) (°)	N/A	155.180(6)<br>155.180(6)	154.21(4)<br>158.09(4)

† From the highest occupancy component of the crystal structure.

Magnetic Property Measurements

Magnetic measurements were recorded on a Quantum Design MPMS-XL7 SQUID magnetometer equipped with a 7 T magnet. The samples were restrained in eicosane and sealed in 7 mm NMR tubes. Direct current (DC) magnetic susceptibility measurements were performed on polycrystalline samples of [**1**][Na(15-crown-5)$(THF)_2$] (16.0 mg), **2** (30.5 mg) and [**3**][K(18-crown-6)$(THF)_2$] (31.3 mg) in the temperature range 1.9-300 K and using an applied field of 1000 Oe. AC susceptibility measurements were performed using an AC field of 3 Oe in zero DC field. Diamagnetic corrections were made using Pascal's constants for all the constituent atoms.[7] Measurements on diluted samples were performed using polycrystalline samples of [**1a**] [Na(15-crown-5)$(THF)_2$] (37.0 mg) and [**3a**][K(18-crown-6)$(THF)_2$] (67.1 mg).

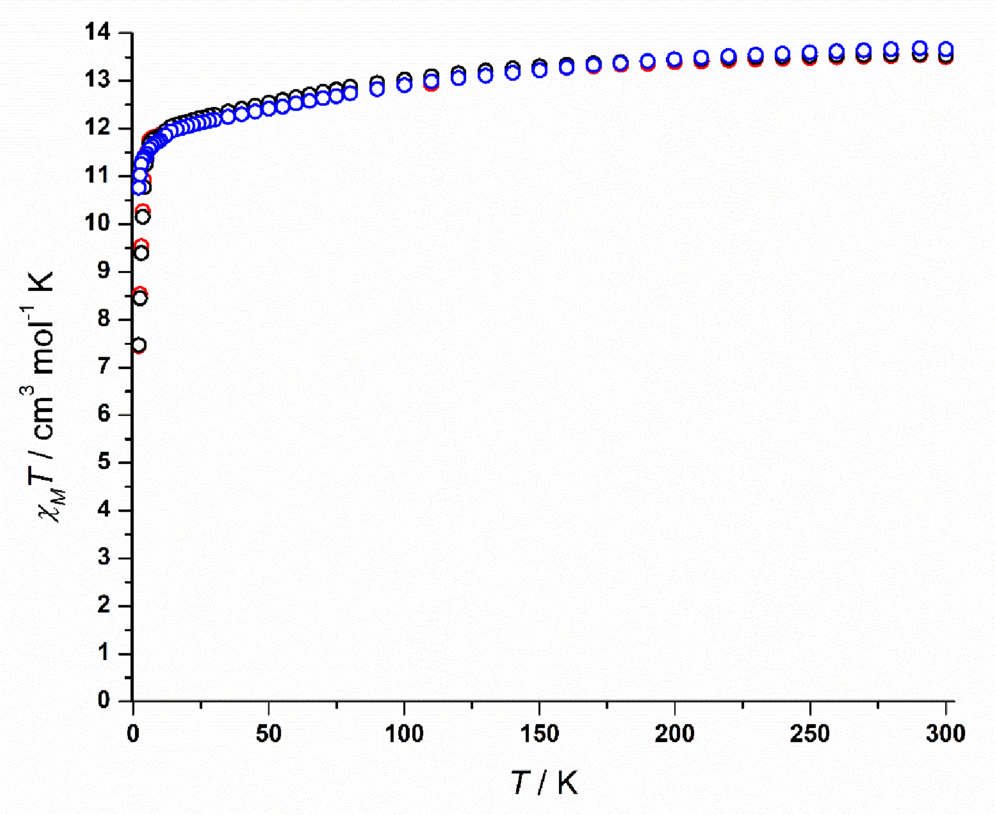

Figure S34. $\chi_M T(T)$ in an applied field of 1000 Oe for [**1**][Na(15-crown-5)$(THF)_2$] (blue circles), **2** (black circles) and [**3**][K(18-crown-6)$(THF)_2$] (red circles).

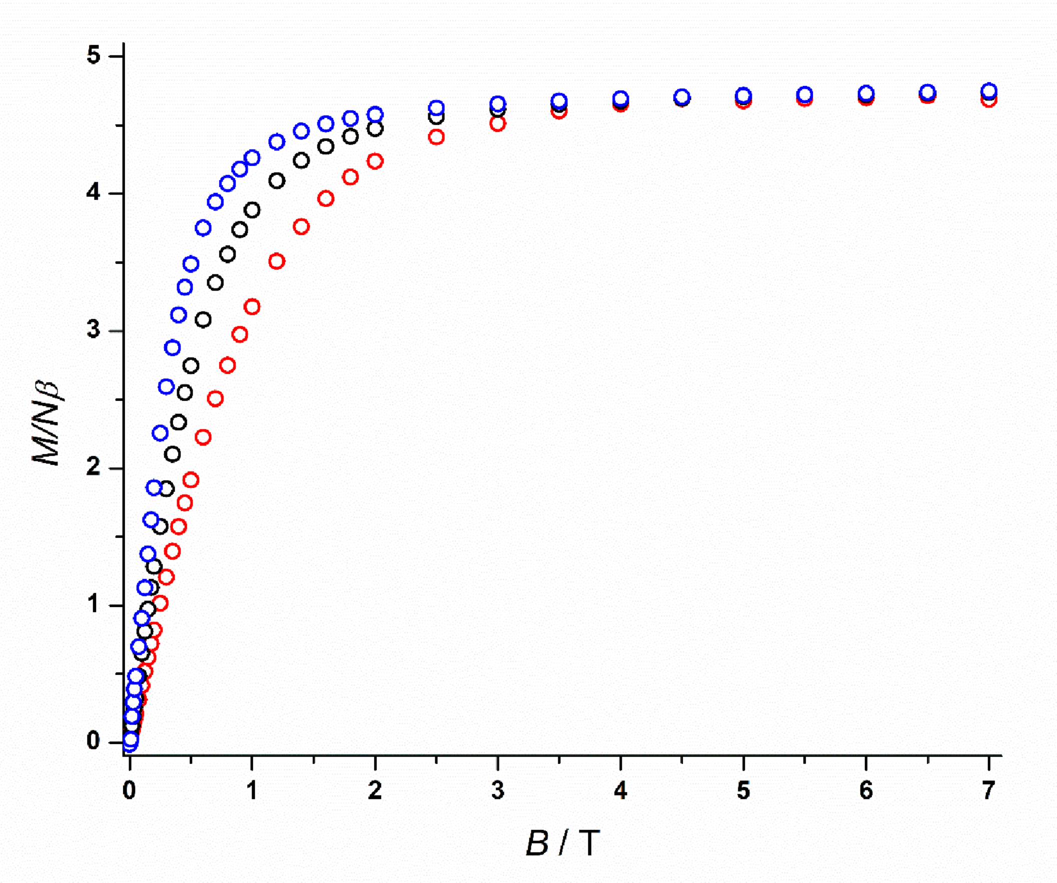

Figure S35. Field (*H*) dependence of the magnetization (*M*) at 1.9 K (blue circles), 3.0 K (black circles) and 5.0 K (red circles) for [**1**][Na(15-crown-5)(THF)$_2$]. *M* = 4.75 *N*β at 1.9 K and 7 T.

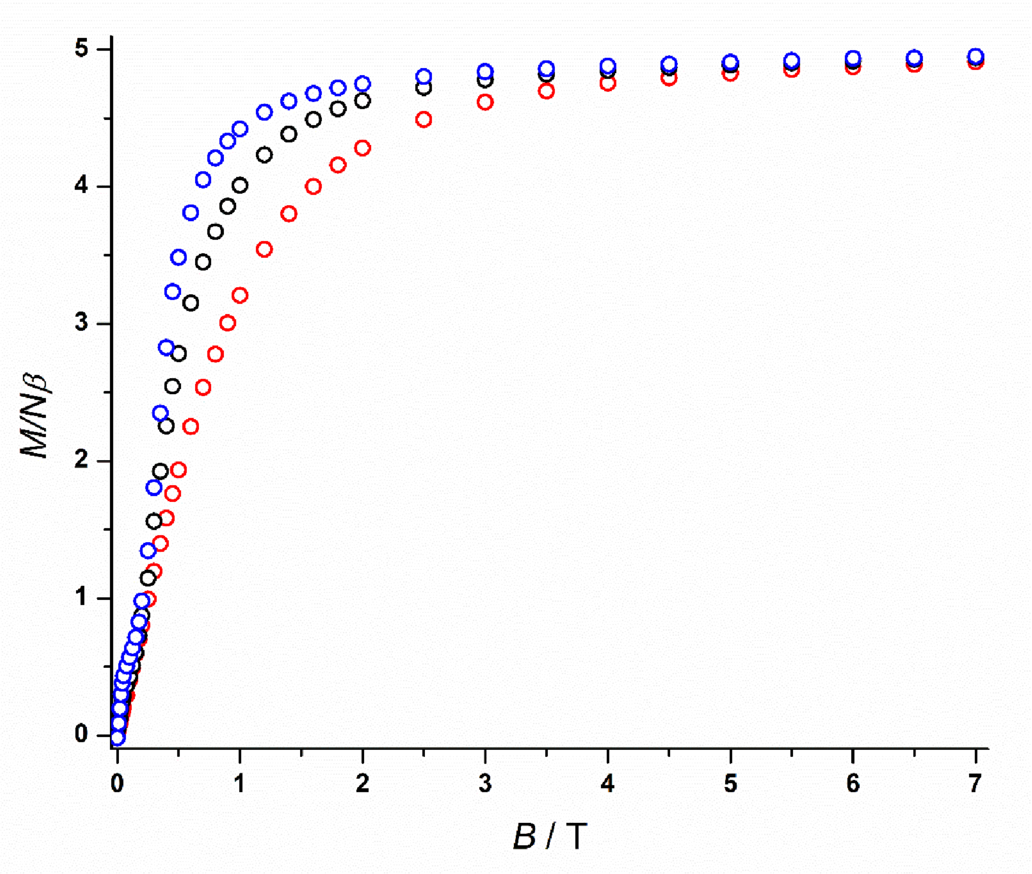

Figure S36. Field (*H*) dependence of the magnetization (*M*) at 1.9 K (blue circles), 3.0 K (black circles) and 5.0 K (red circles) for **2**. *M* = 4.95 *N*β at 1.9 K and 7 T.

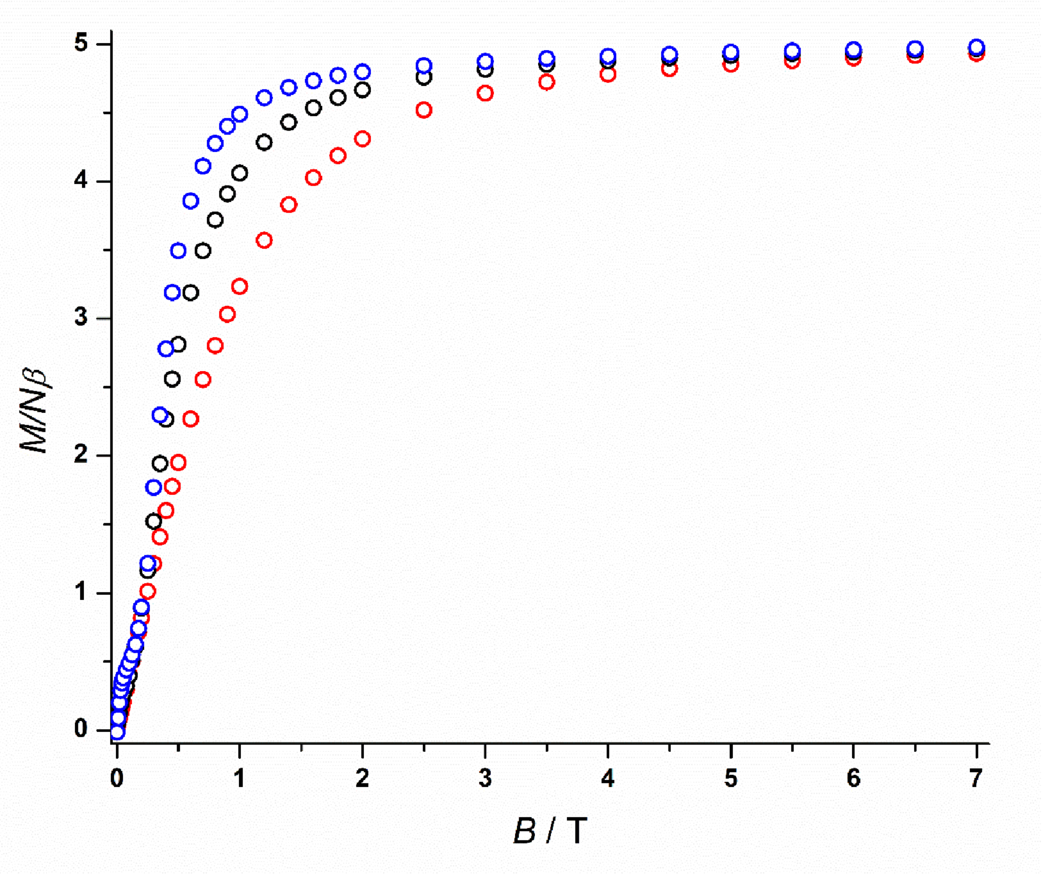

Figure S37. Field (H) dependence of the magnetization (M) at 1.9 K (blue circles), 3.0 K (black circles) and 5.0 K (red circles) for [**3**][K(18-crown-6)(THF)$_2$]. M = 4.98 $N\beta$ at 1.9 K and 7 T.

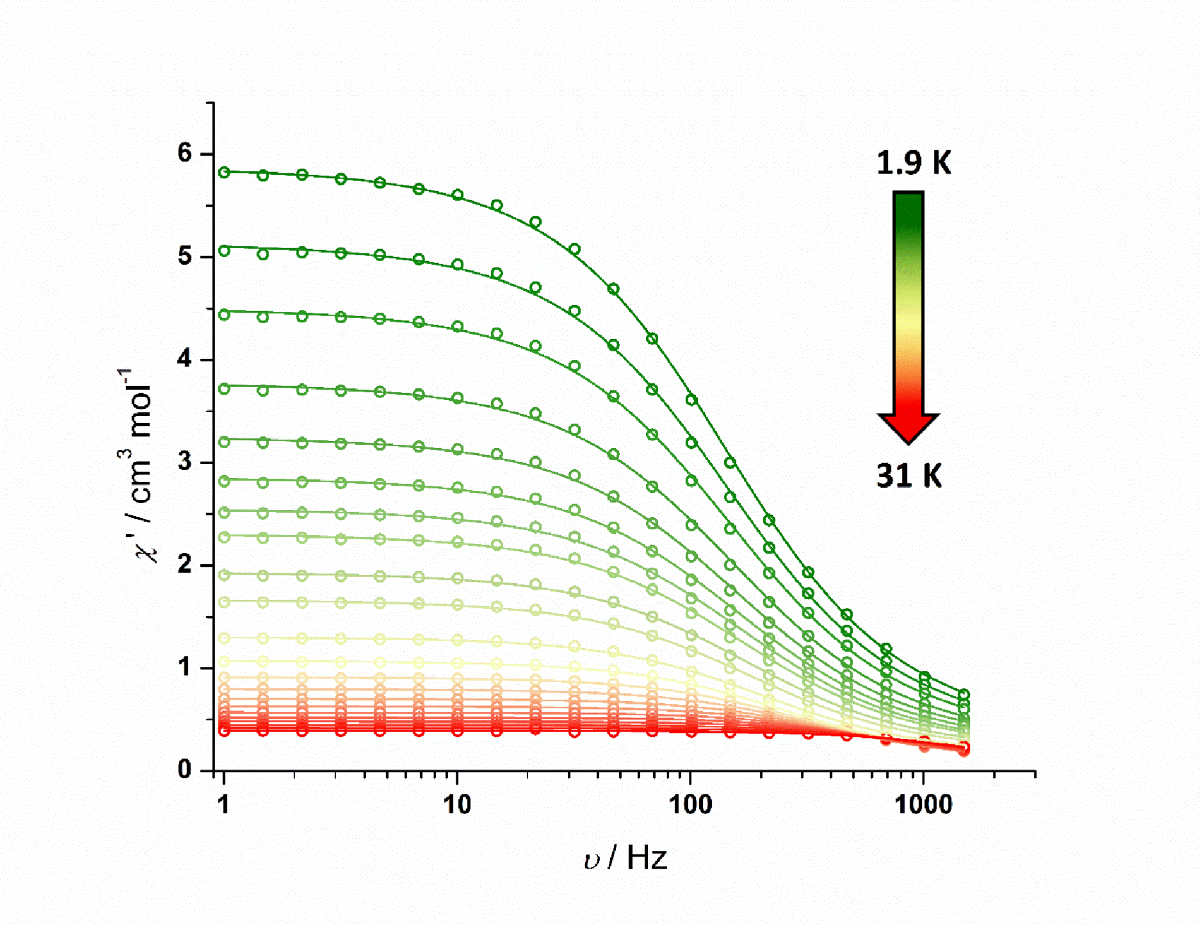

Figure S38. Frequency dependence of the in-phase susceptibility (χ') for [**1**][Na(15-crown-5)(THF)$_2$] in zero DC field at ν = 1-1488 Hz and temperatures of 1.9-31 K. Solid lines represent fits to the data using equation 1.[8]

$$\chi'(\nu_{ac}) = \chi_{\infty} + \frac{(\chi_{s} - \chi_{\infty})[1 + (2\pi\nu_{ac}\tau)^{1-\alpha}\sin(\alpha\pi/2)]}{1 + 2(2\pi\nu_{ac}\tau)^{1-\alpha}\sin(\alpha\pi/2) + (2\pi\nu_{ac}\tau)^{2(1-\alpha)}} \quad \text{Equation 1}$$

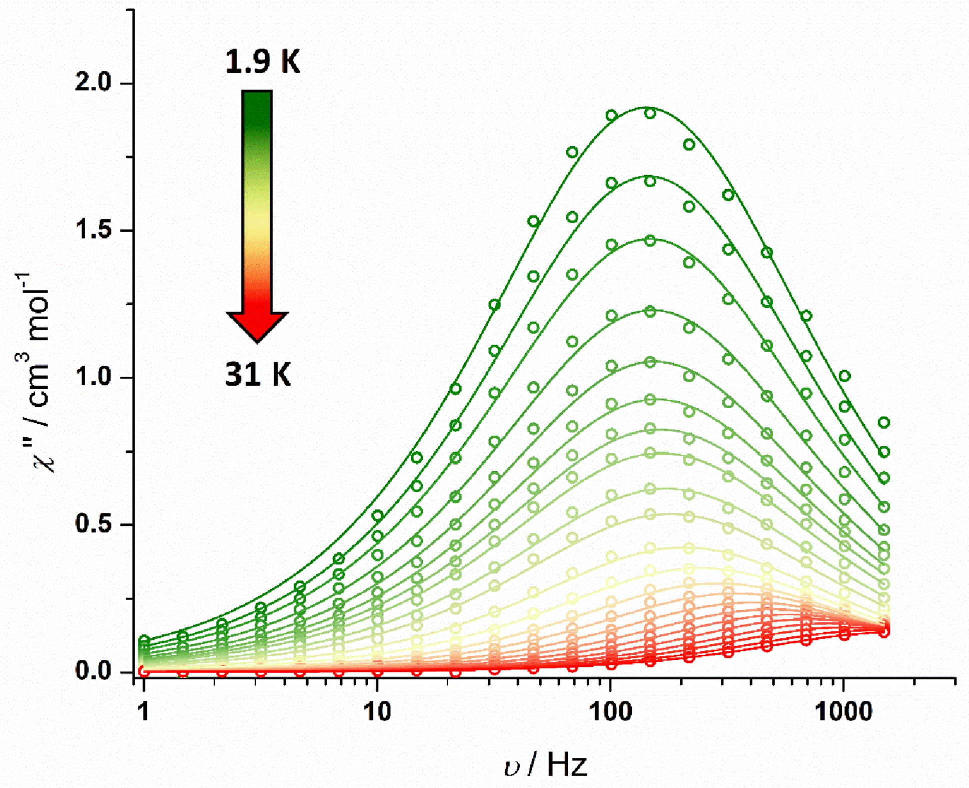

Figure S39. Frequency dependence of the out-of-phase susceptibility (χ'') for [**1**][Na(15-crown-5)(THF)$_2$] in zero DC field at ν = 1-1488 Hz and temperatures of 1.9-31 K. Solid lines represent fits to the data using equation 2.[8]

$$\chi''(\nu_{ac}) = \frac{(\chi_S - \chi_\infty)(2\pi\nu_{ac}\tau)^{1-\alpha}\cos(\alpha\pi/2)}{1+2(2\pi\nu_{ac}\tau)^{1-\alpha}\sin(\alpha\pi/2)+(2\pi\nu_{ac}\tau)^{2(1-\alpha)}} \quad \text{Equation 2}$$

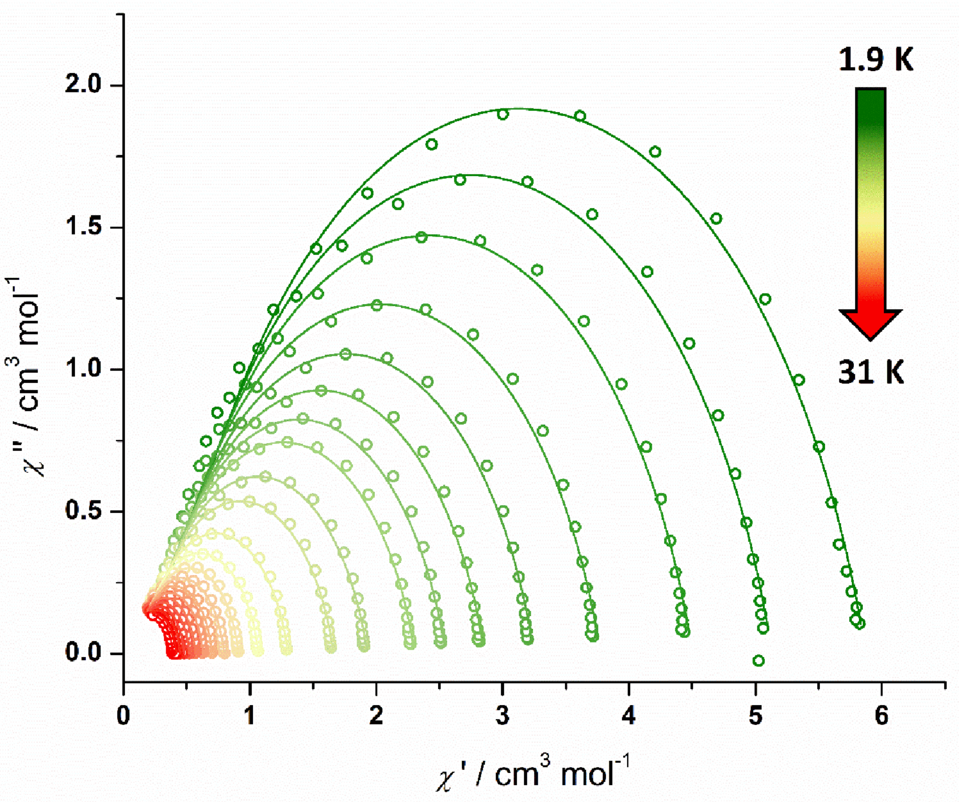

Figure S40. Cole-Cole plots for the AC susceptibilities in zero DC field for [**1**][Na(15-crown-5)(THF)$_2$] from 1.9-31 K. Solid lines represent fits to the data using equations 1 and 2.

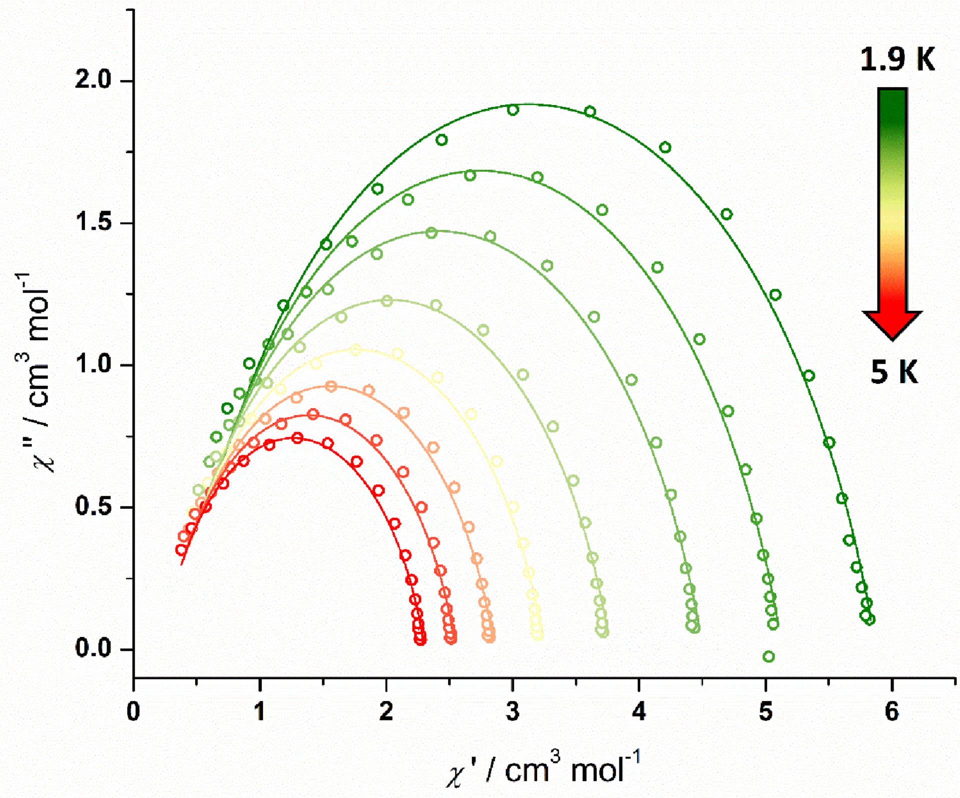

Figure S41. Cole-Cole plots for the AC susceptibilities in zero DC field for [**1**][Na(15-crown-5)(THF)$_2$] from 1.9-5 K. Solid lines represent fits to the data using equations 1 and 2.

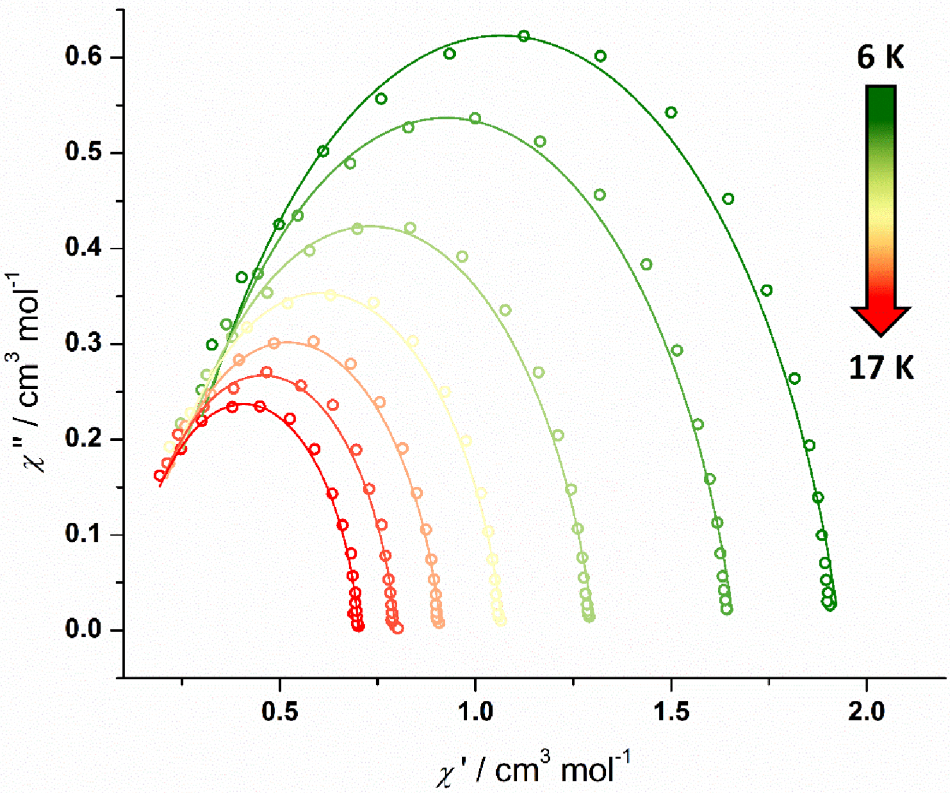

Figure S42. Cole-Cole plots for the AC susceptibilities in zero DC field for [**1**][Na(15-crown-5)(THF)$_2$] from 6-17 K. Solid lines represent fits to the data using equations 1 and 2.

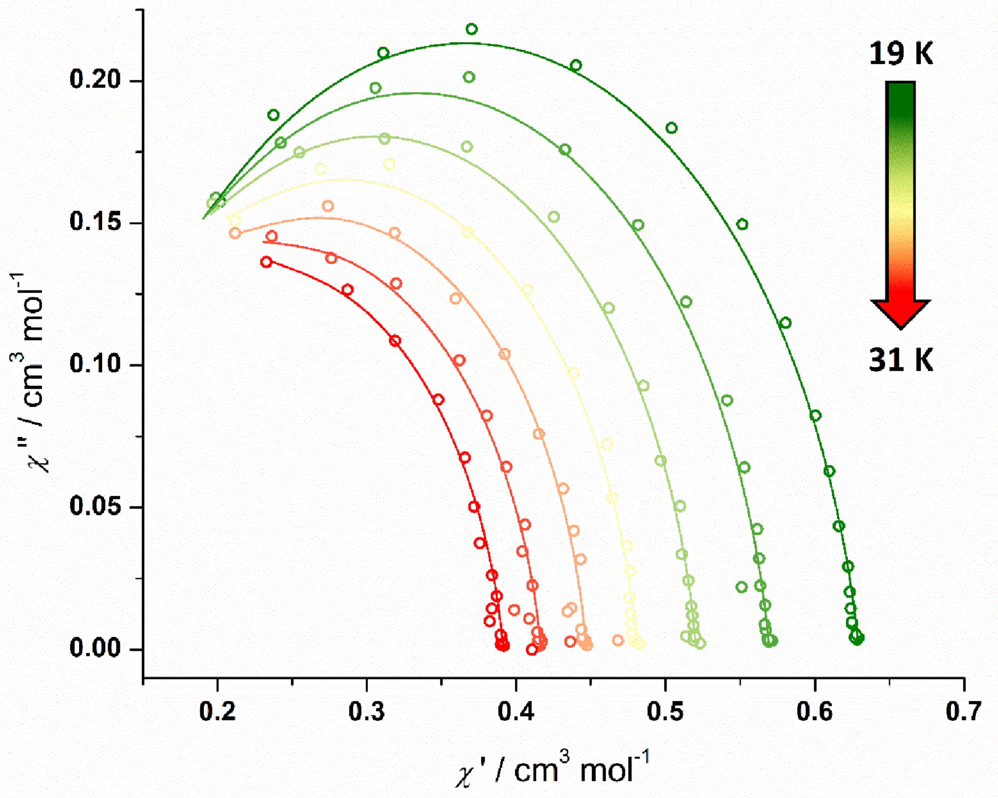

Figure S43. Cole-Cole plots for the AC susceptibilities in zero DC field for [**1**][Na(15-crown-5)(THF)$_2$] from 19-31 K. Solid lines represent fits to the data using equations 1 and 2.

Table S4. Relaxation fitting parameters for [**1**][Na(15-crown-5)(THF)$_2$] corresponding to Figures S40-S43.

T / K	χ_T / cm^3 mol^{-1}	χ_S / cm^3 mol^{-1}	α	τ / s
1.9	5.87479	0.37708	0.21770	0.00112
2.2	5.13241	0.35678	0.21102	0.00109
2.5	4.50309	0.32720	0.21135	0.00108
3	3.77442	0.28369	0.21171	0.00105
3.5	3.24832	0.26947	0.20846	0.00103
4	2.85615	0.25430	0.20495	0.00101
4.5	2.55000	0.22802	0.20672	0.00098
5	2.30379	0.22646	0.20170	0.00097
6	1.93099	0.19881	0.19878	0.00092
7	1.66217	0.18871	0.19081	0.00088
9	1.30290	0.15816	0.18161	0.00077
11	1.06990	0.13662	0.16689	0.00066
13	0.91093	0.13079	0.15348	0.00056
15	0.79619	0.11639	0.14302	0.00046
17	0.70214	0.11228	0.12881	0.00039
19	0.62967	0.10196	0.12501	0.00032
21	0.56957	0.09720	0.10859	0.00027
23	0.52052	0.09143	0.09930	0.00022
25	0.48092	0.08862	0.09847	0.00018
27	0.44692	0.08875	0.09372	0.00016
29	0.41671	0.07336	0.11116	0.00012
31	0.39144	0.06707	0.10905	0.00010

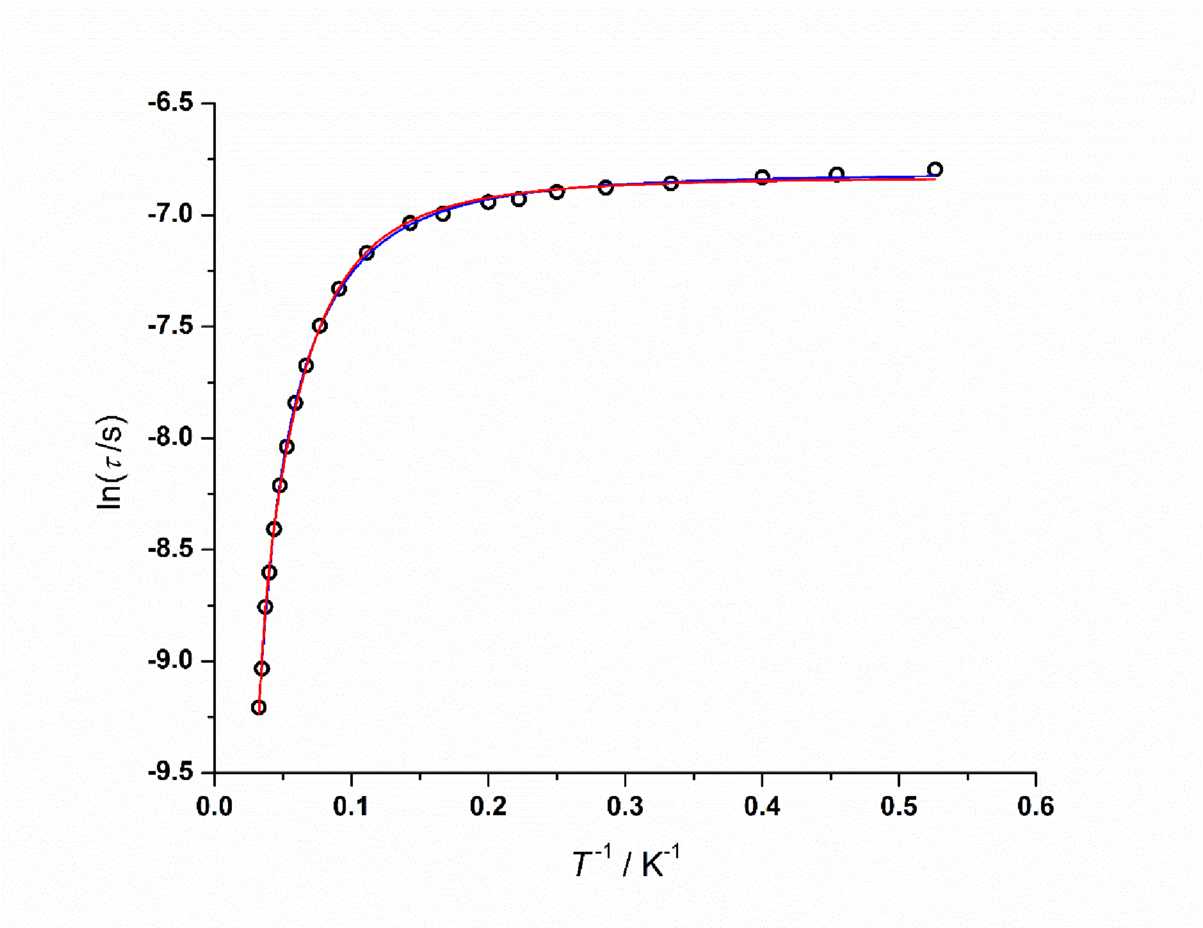

Figure S44. Plot of natural log of the relaxation time (τ) *vs.* inverse temperature for [**1**][Na(15-crown-5)(THF)$_2$].

The red line is the best fit (adjusted R^2 = 0.99918) to $\tau^{-1} = \tau_0^{-1}e^{-U_{\mathrm{eff}}/k_{\mathrm{B}}T} + CT^n + \tau_{QTM}^{-1}$, giving: U_{eff} (fixed) = 242 cm^{-1}, τ_0 = 6.0(9) × 10^{-9} s, C = 1.9(3) s^{-1} K^{-n}, n = 2.39(5) and τ_{QTM} = 1.08(1) × 10^{-3} s.

The blue line is the best fit (adjusted R^2 = 0.99948) to $\tau^{-1} = \tau_0^{-1}e^{-U_{\mathrm{eff}}/k_{\mathrm{B}}T} + CT^n + \tau_{QTM}^{-1}$, giving: U_{eff} = 127(17) cm^{-1}, τ_0 = 9.0(6) × 10^{-7} s, C = 3.5(8) s^{-1} K^{-n}, n = 2.17(8) and τ_{QTM} = 1.10(1) × 10^{-3} s.

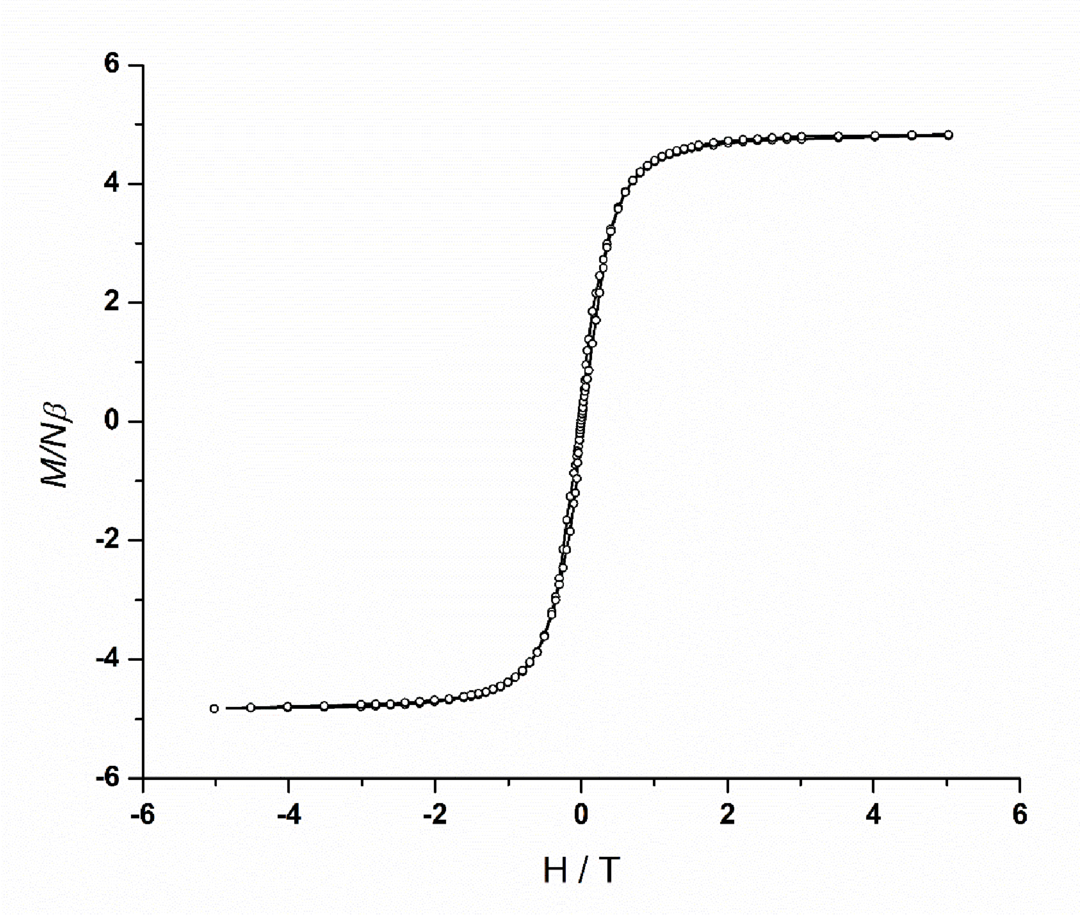

Figure S45. Magnetic hysteresis loops for [**1**][Na(15-crown-5)(THF)$_2$]. The data were continuously collected at 1.9 K under a varying field sweep rate (1.1 mT s^{-1} |0-1| T, 3.0 mT s^{-1} |1-2| T, 4.5 mT s^{-1} |2-3| T and 8.5 mT s^{-1} |3-5| T). Solid lines are a guide to the eye.

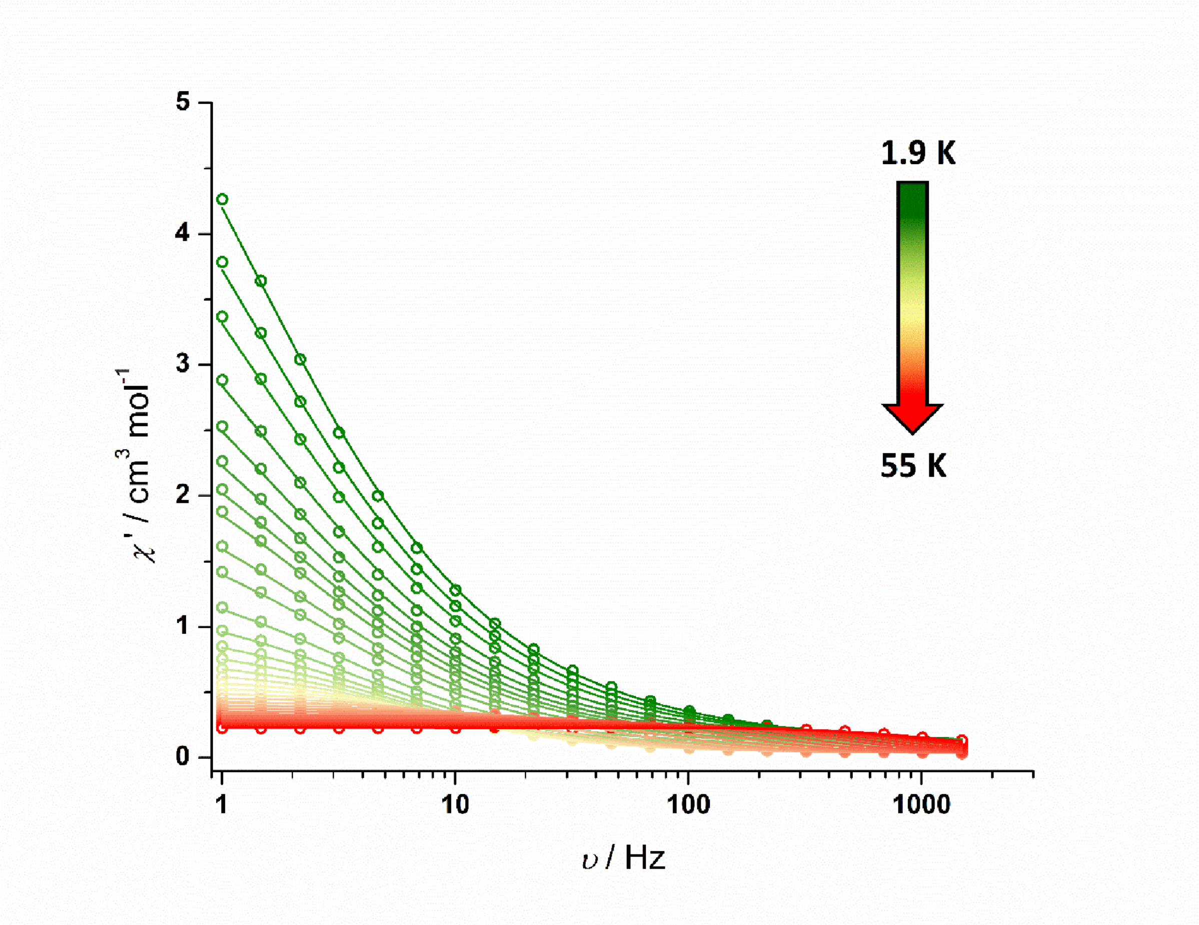

Figure S46. Frequency dependence of the in-phase susceptibility (χ') for **2** in zero DC field at ν = 1-1488 Hz and temperatures of 1.9-55 K. Solid lines represent fits to the data using equation 1.[8]

$$\chi'(\nu_{ac}) = \chi_{\infty} + \frac{(\chi_{s} - \chi_{\infty})[1 + (2\pi\nu_{ac}\tau)^{1-\alpha}\sin(\alpha\pi/2)]}{1 + 2(2\pi\nu_{ac}\tau)^{1-\alpha}\sin(\alpha\pi/2) + (2\pi\nu_{ac}\tau)^{2(1-\alpha)}} \qquad \text{Equation 1}$$

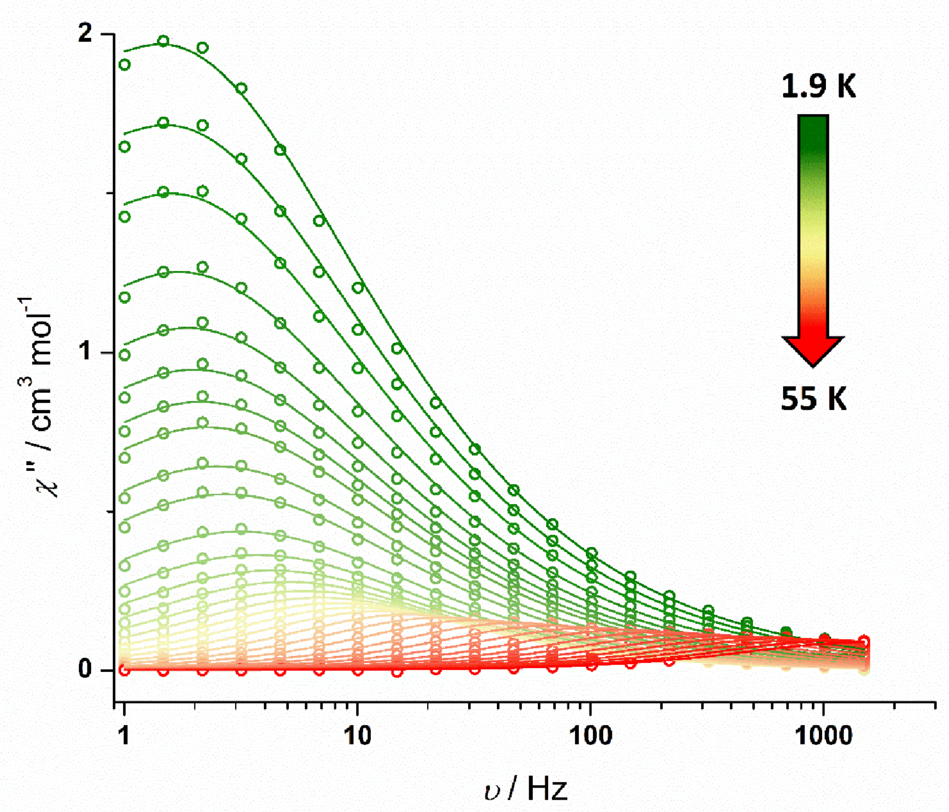

Figure S47. Frequency dependence of the out-of-phase susceptibility (χ'') for **2** in zero DC field at ν = 1-1488 Hz and temperatures of 1.9-55 K. Solid lines represent fits to the data using equation 2.[8]

$$\chi''(\nu_{ac}) = \frac{(\chi_s - \chi_\infty)(2\pi\nu_{ac}\tau)^{1-\alpha}\cos(\alpha\pi/2)}{1+2(2\pi\nu_{ac}\tau)^{1-\alpha}\sin(\alpha\pi/2)+(2\pi\nu_{ac}\tau)^{2(1-\alpha)}} \quad \text{Equation 2}$$

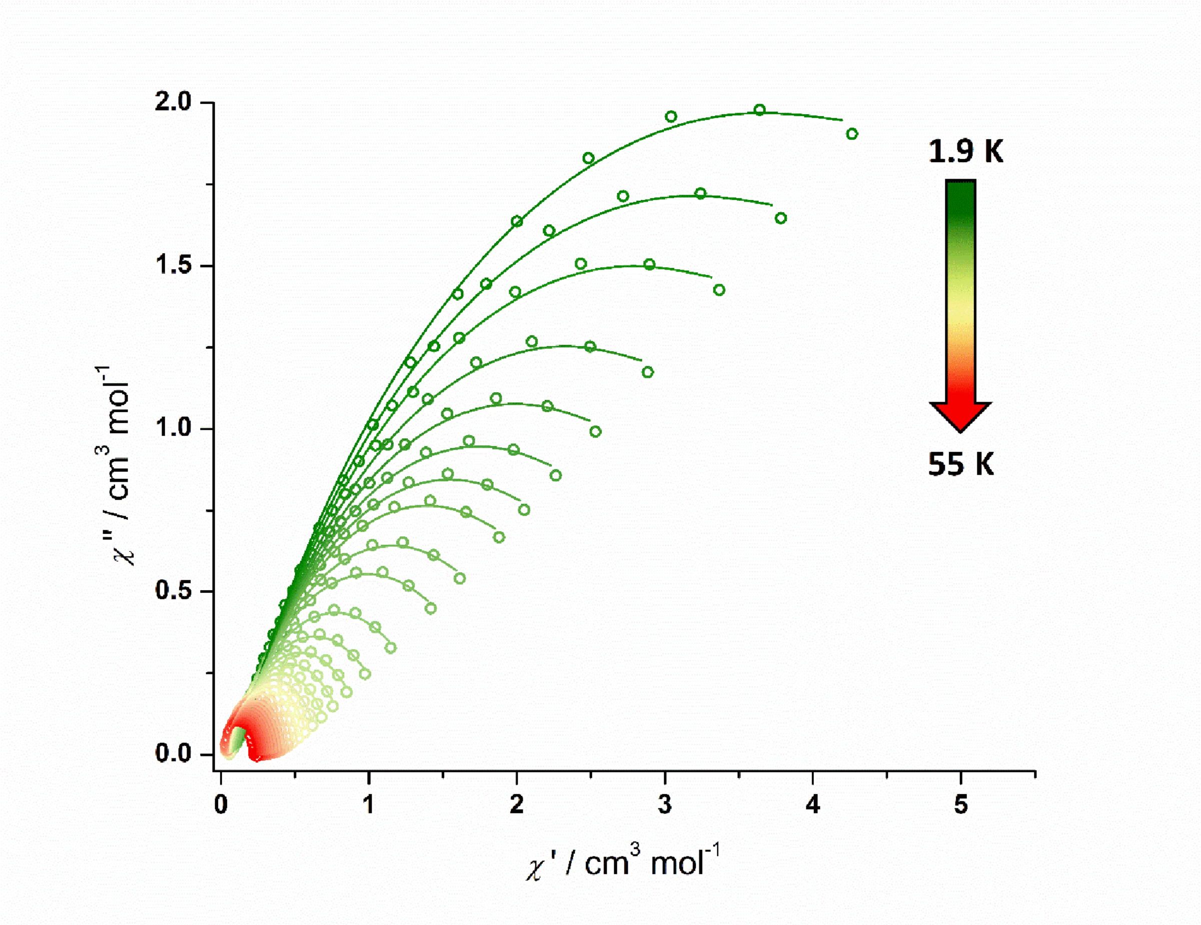

Figure S48. Cole-Cole plots for the AC susceptibilities in zero DC field for **2** from 1.9-55 K. Solid lines represent fits to the data using equations 1 and 2.

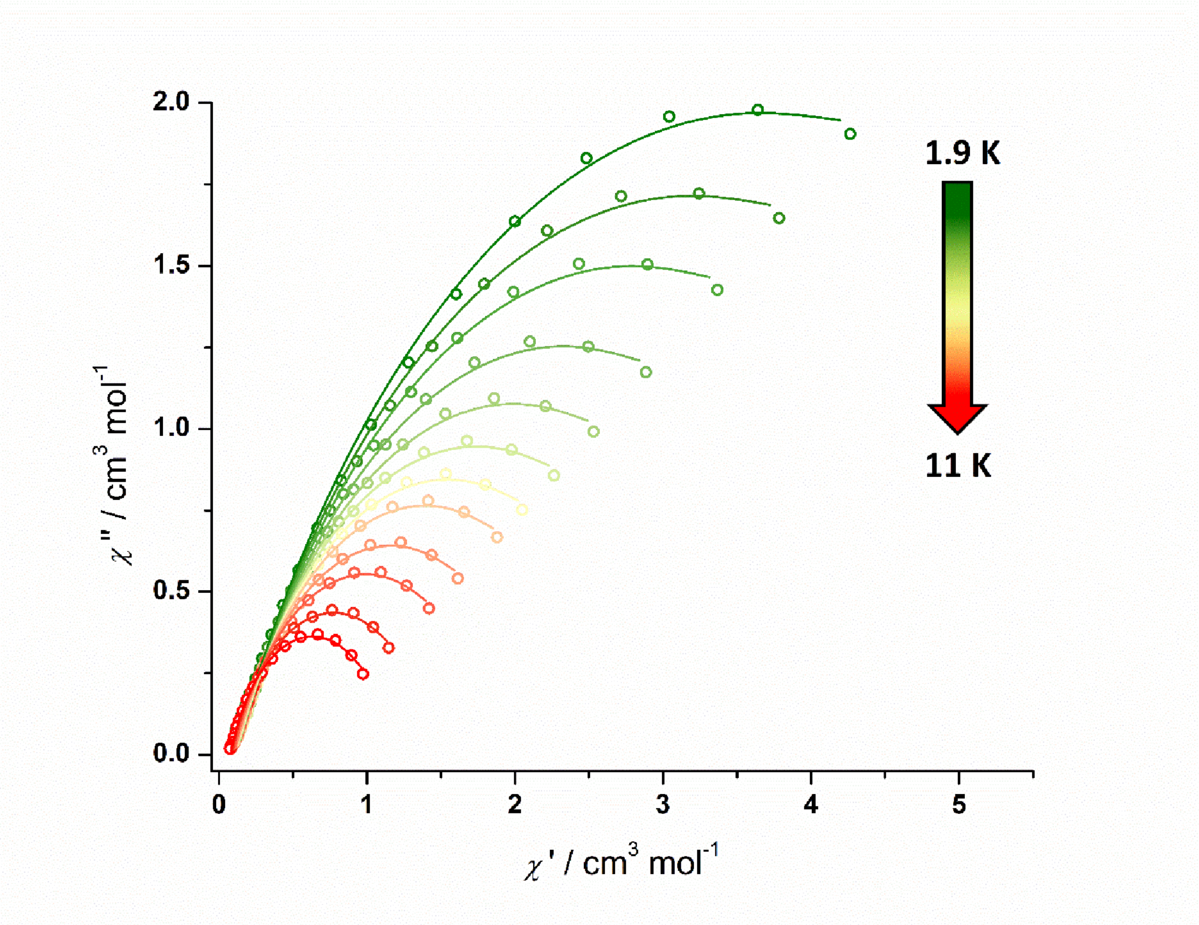

Figure S49. Cole-Cole plots for the AC susceptibilities in zero DC field for **2** from 1.9-11 K. Solid lines represent fits to the data using equations 1 and 2.

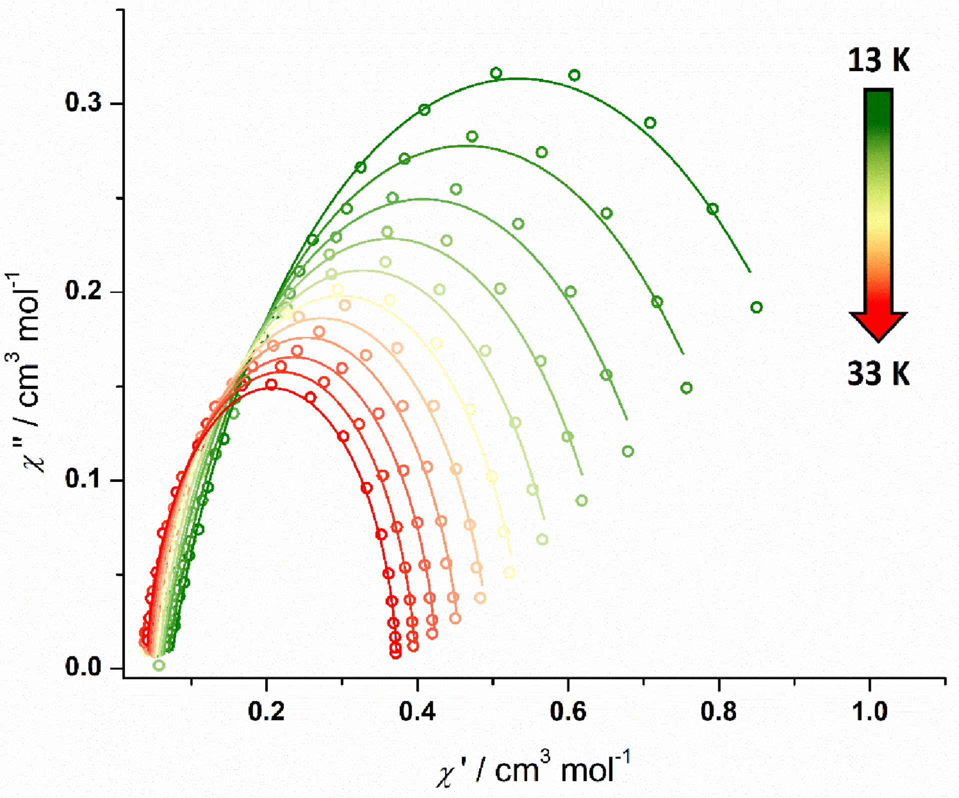

Figure S50. Cole-Cole plots for the AC susceptibilities in zero DC field for **2** from 13-33 K. Solid lines represent fits to the data using equations 1 and 2.

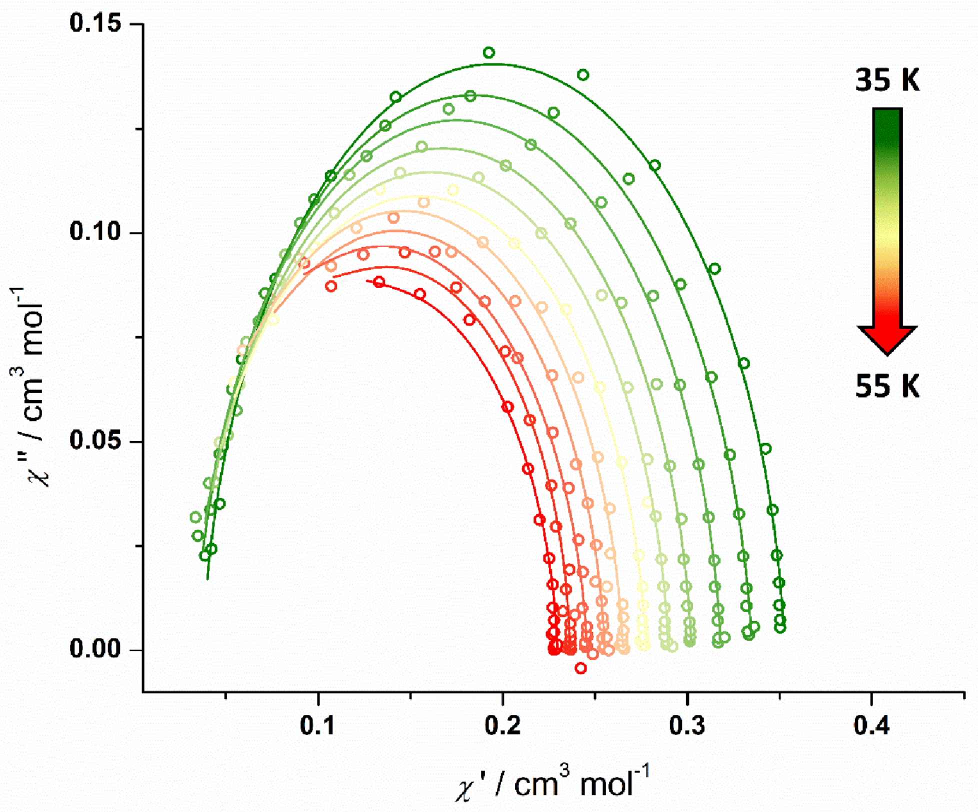

Figure S51. Cole-Cole plots for the AC susceptibilities in zero DC field for **2** from 35-55 K. Solid lines represent fits to the data using equations 1 and 2.

Table S5. Relaxation fitting parameters for **2** corresponding to Figures S48-S51.

T / K	χ_T / cm^3 mol^{-1}	χ_S / cm^3 mol^{-1}	α	τ / s
1.9	7.21172	0.10109	0.35247	0.11077
2.2	6.28422	0.10505	0.35128	0.10593
2.5	5.47238	0.11096	0.34708	0.10054
3	4.53654	0.11571	0.33962	0.09267
3.5	3.86217	0.11508	0.33206	0.08564
4	3.36595	0.11359	0.32510	0.08006
4.5	2.97852	0.11146	0.31796	0.07507
5	2.67465	0.10873	0.31182	0.07094
6	2.21822	0.10190	0.30177	0.06410
7	1.89151	0.09485	0.29212	0.05861
9	1.46392	0.08412	0.27744	0.05028
11	1.18624	0.07648	0.25698	0.04320
13	0.99793	0.07115	0.23653	0.03706
15	0.86174	0.06697	0.21653	0.03185
17	0.75416	0.06077	0.19875	0.02708
19	0.66872	0.05886	0.17333	0.02295
21	0.60055	0.05563	0.15062	0.01923
23	0.54490	0.05193	0.12930	0.01577
25	0.49850	0.04865	0.10946	0.01259
27	0.45821	0.04643	0.08886	0.00953
29	0.42660	0.04300	0.08204	0.00691
31	0.39789	0.04127	0.06665	0.00494
33	0.37348	0.03993	0.05887	0.00337
35	0.35275	0.03723	0.06163	0.00234
37	0.33505	0.03293	0.06886	0.00160
39	0.31818	0.03225	0.06322	0.00112
41	0.30222	0.03314	0.05881	0.00080
43	0.28984	0.03260	0.06114	0.00058
45	0.27777	0.03094	0.06818	0.00042
47	0.26516	0.02710	0.06614	0.00030
49	0.25523	0.02994	0.06019	0.00024
51	0.24575	0.02446	0.07296	0.00017
53	0.23636	0.03865	0.03343	0.00015
55	0.22971	0.03373	0.06338	0.00011

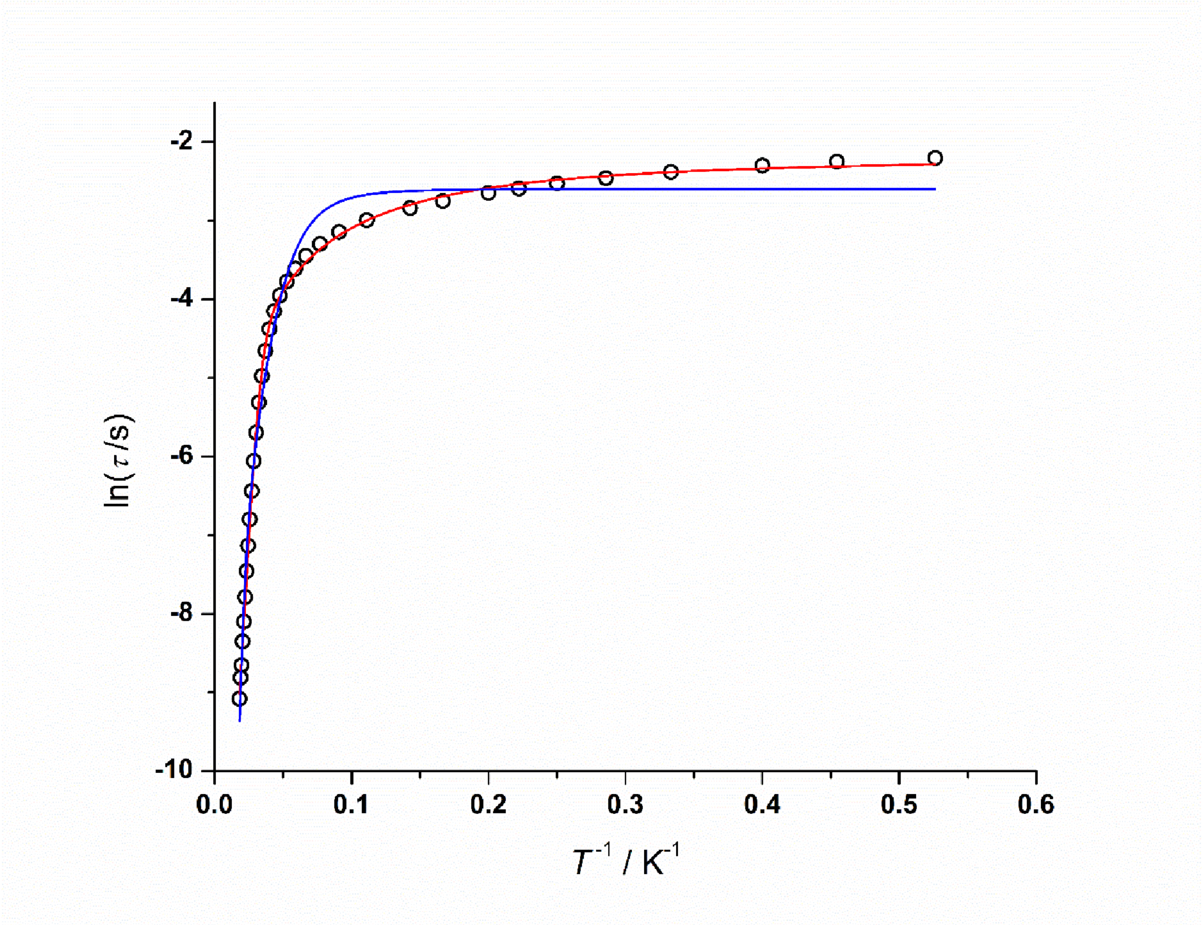

Figure S52. Plot of natural log of the relaxation time (τ) *vs.* inverse temperature for **2**.

The red line is the best fit (adjusted R^2 = 0.99945) to $\tau^{-1} = \tau_0^{-1}e^{-U_{\text{eff}}/k_{\text{B}}T} + CT^n + \tau_{QTM}^{-1}$, giving: U_{eff} = 213(3) cm^{-1}, τ_0 = 4.76(5) × 10^{-7} s, C = 0.34(8) s^{-1} K^{-n}, n = 1.58(8) and τ_{QTM} = 0.114(5) s.

The blue line is the best fit (adjusted R^2 = 0.98858) to $\tau^{-1} = \tau_0^{-1}e^{-U_{\text{eff}}/k_{\text{B}}T} + CT^n + \tau_{QTM}^{-1}$, giving: U_{eff} (fixed) = 447 cm^{-1}, τ_0 = 9.8(2) × 10^{-10} s, C = 5(5) × 10^{-5} s^{-1} K^{-n}, n = 4.5(3) and τ_{QTM} = 0.075(5) s.

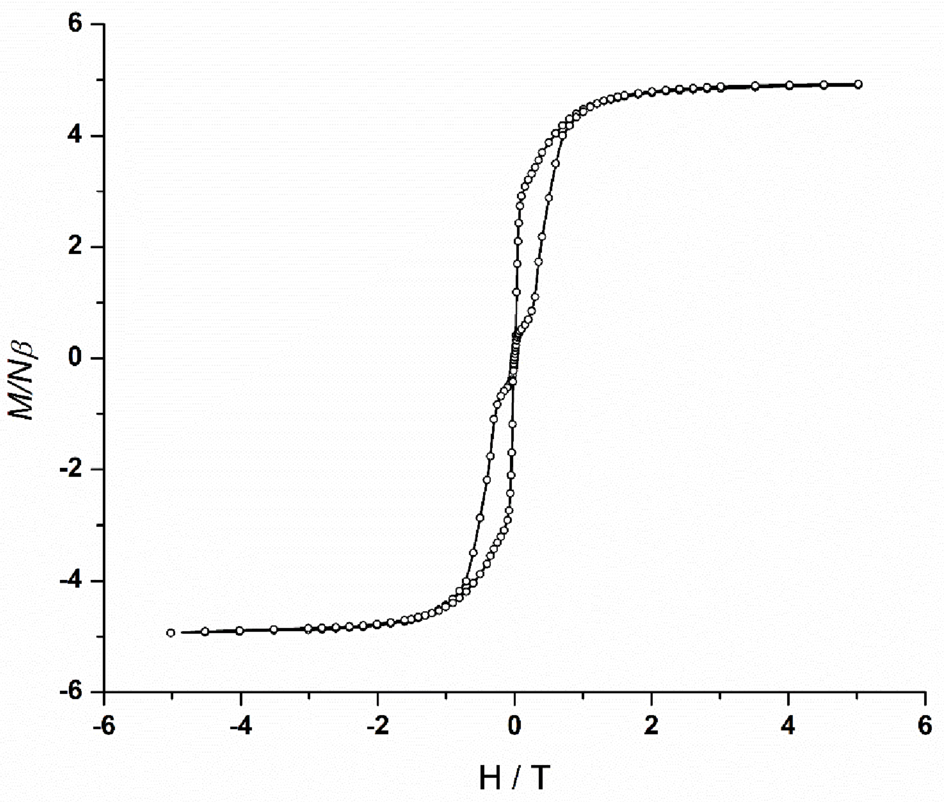

Figure S53. Magnetic hysteresis loops for **2**. The data were continuously collected at 1.9 K under a varying field sweep rate (1.1 mT s^{-1} |0-1| T, 3.0 mT s^{-1} |1-2| T, 4.5 mT s^{-1} |2-3| T and 8.5 mT s^{-1} |3-5| T). Solid lines are a guide to the eye.

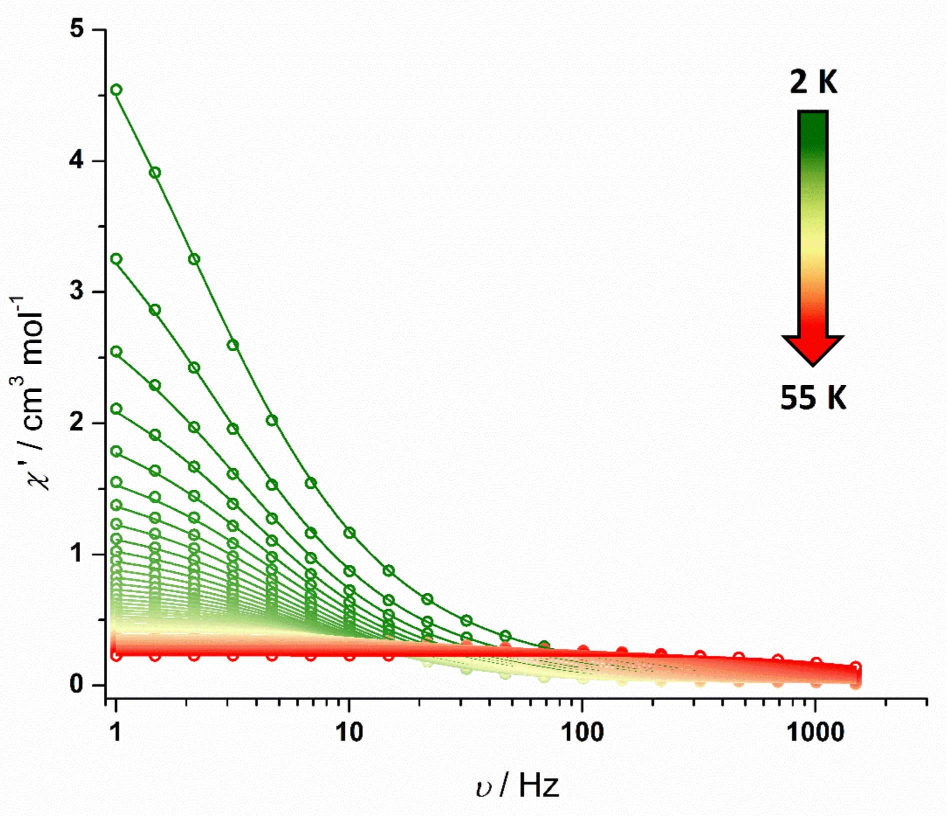

Figure S54. Frequency dependence of the in-phase susceptibility (χ') for [**3**][K(18-crown-6)(THF)$_2$] in zero DC field at ν = 1-1488 Hz and temperatures of 2-55 K. Solid lines represent fits to the data using equation 1.[8]

$$\chi'(\nu_{ac}) = \chi_\infty + \frac{(\chi_s - \chi_\infty)[1+(2\pi\nu_{ac}\tau)^{1-\alpha}\sin(\alpha\pi/2)]}{1+2(2\pi\nu_{ac}\tau)^{1-\alpha}\sin(\alpha\pi/2)+(2\pi\nu_{ac}\tau)^{2(1-\alpha)}} \qquad \text{Equation 1}$$

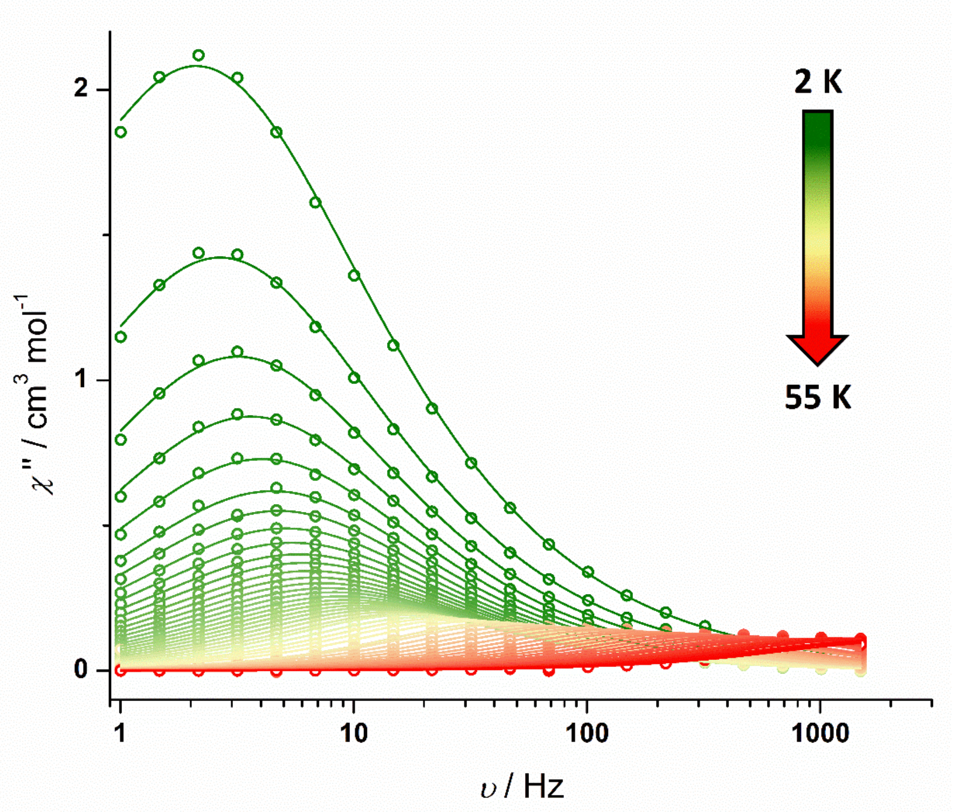

Figure S55. Frequency dependence of the out-of-phase susceptibility (χ'') for [**3**][K(18-crown-6)(THF)$_2$] in zero DC field at ν = 1-1488 Hz and temperatures of 2-55 K. Solid lines represent fits to the data using equation 2.[8]

$$\chi''(\nu_{ac}) = \frac{(\chi_s - \chi_\infty)(2\pi\nu_{ac}\tau)^{1-\alpha}\cos(\alpha\pi/2)}{1 + 2(2\pi\nu_{ac}\tau)^{1-\alpha}\sin(\alpha\pi/2) + (2\pi\nu_{ac}\tau)^{2(1-\alpha)}} \quad \text{Equation 2}$$

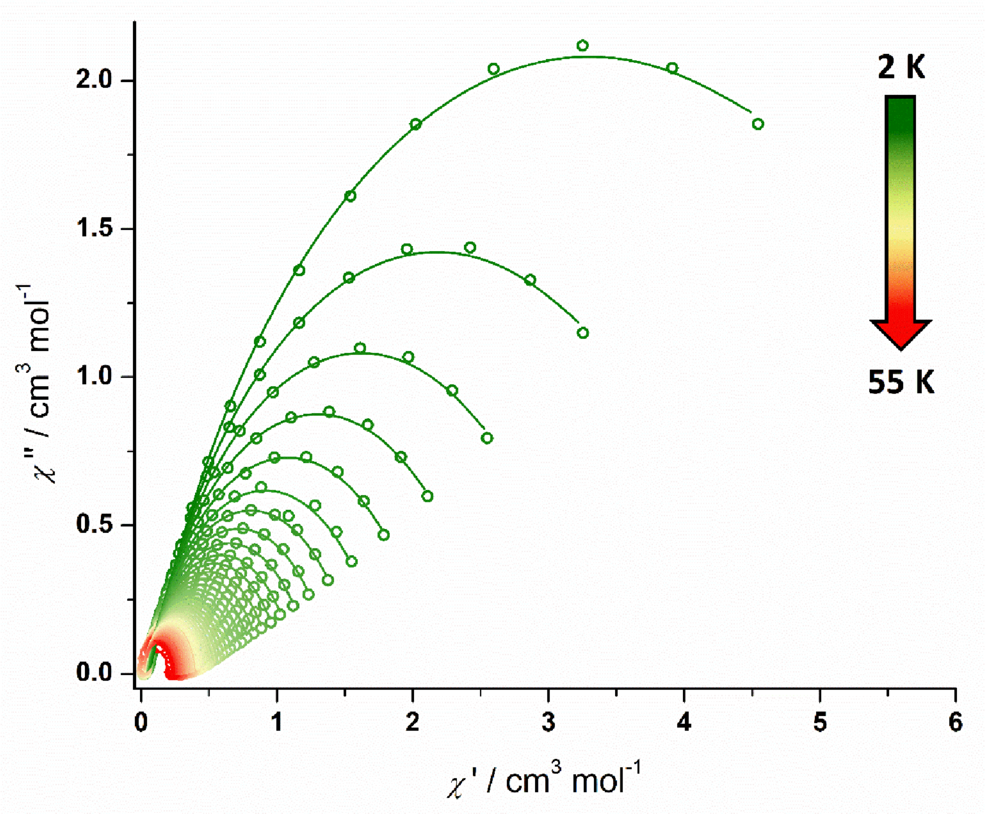

Figure S56. Cole-Cole plots for the AC susceptibilities in zero DC field for [**3**][K(18-crown-6)(THF)$_2$] from 2-55 K. Solid lines represent fits to the data using equations 1 and 2.

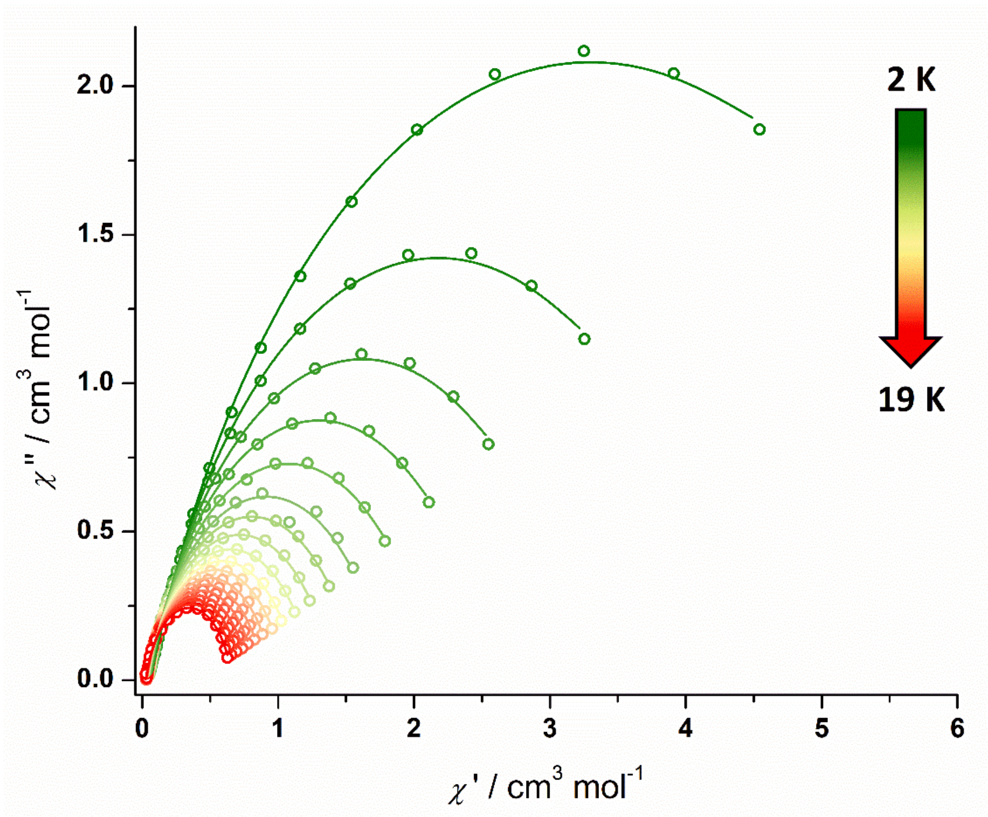

Figure S57. Cole-Cole plots for the AC susceptibilities in zero DC field for [**3**][K(18-crown-6)(THF)$_2$] from 2-19 K. Solid lines represent fits to the data using equations 1 and 2.

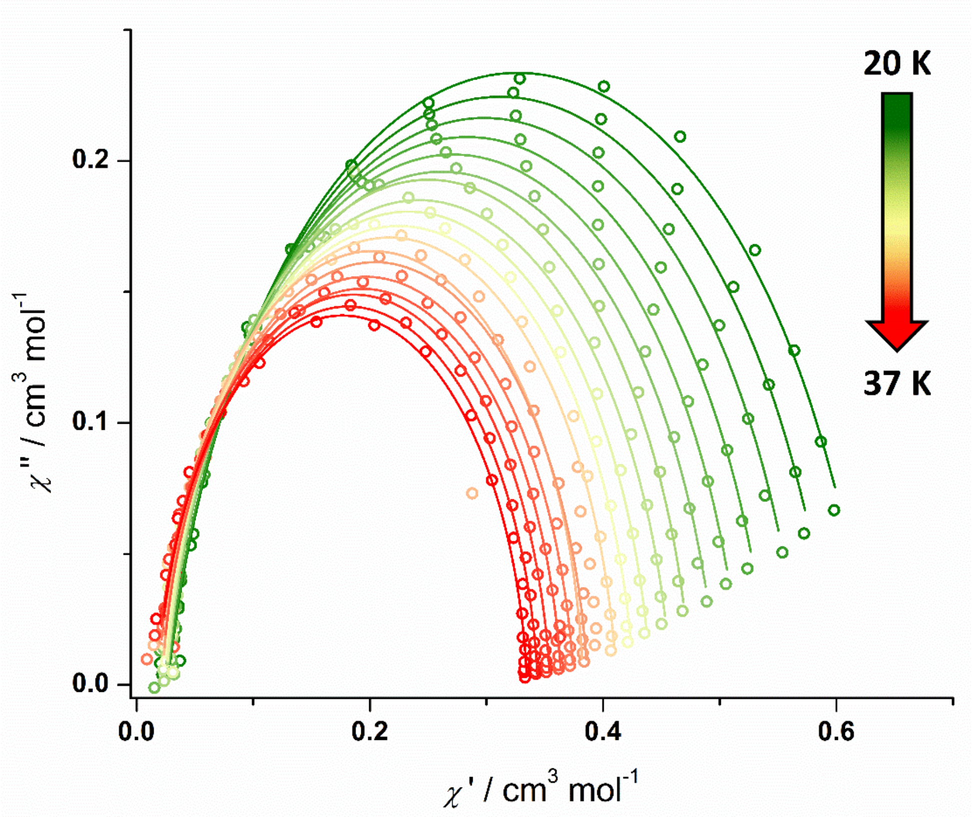

Figure S58. Cole-Cole plots for the AC susceptibilities in zero DC field for [**3**][K(18-crown-6)(THF)$_2$] from 20-37 K. Solid lines represent fits to the data using equations 1 and 2.

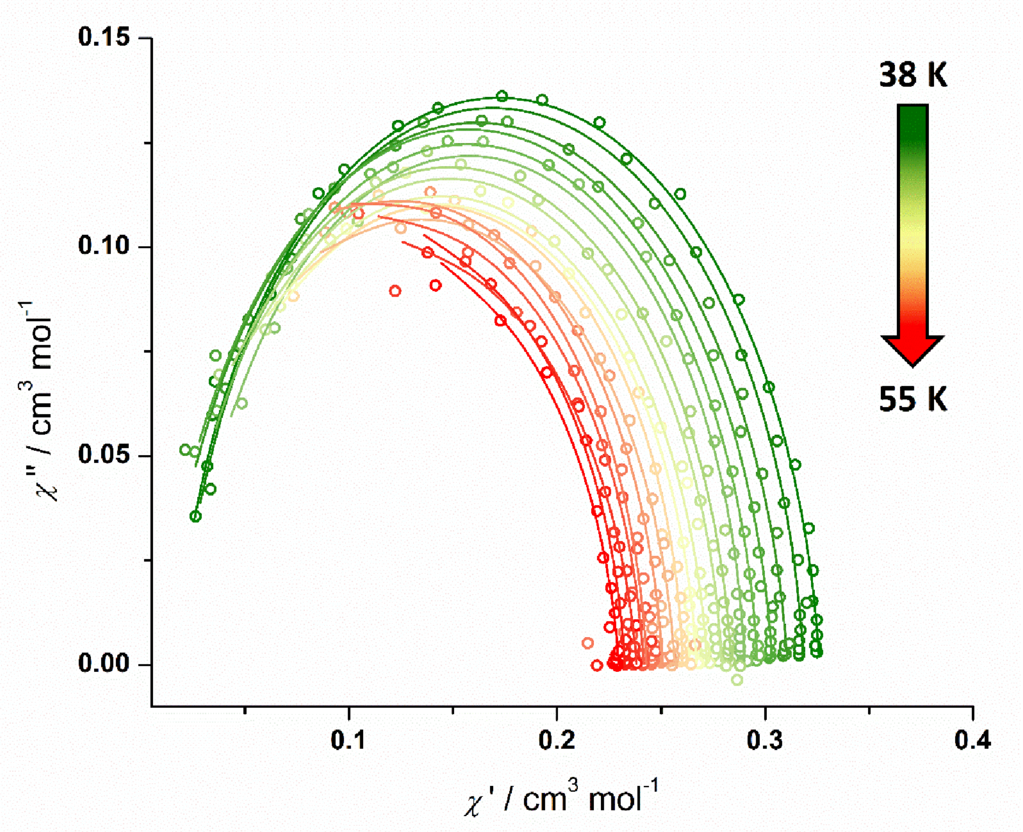

Figure S59. Cole-Cole plots for the AC susceptibilities in zero DC field for [**3**][K(18-crown-6)(THF)$_2$] from 38-55 K. Solid lines represent fits to the data using equations 1 and 2.

Table S6. Relaxation fitting parameters for [**3**][K(18-crown-6)(THF)$_2$] corresponding to Figures S56-S59.

T / K	χ_T / cm^3 mol^{-1}	χ_S / cm^3 mol^{-1}	α	τ / s
2	6.53947	0.05827	0.26765	0.07548
3	4.29565	0.06177	0.24091	0.05967
4	3.18636	0.05897	0.22348	0.04989
5	2.54420	0.05341	0.21362	0.04393
6	2.10828	0.04978	0.20870	0.03949
7	1.78101	0.04648	0.20479	0.03543
8	1.56988	0.04271	0.19788	0.03345
9	1.39377	0.04069	0.19528	0.03116
10	1.25477	0.03680	0.19542	0.02923
11	1.14068	0.03549	0.19443	0.02745
12	1.04791	0.03284	0.18873	0.02588
13	0.96607	0.03420	0.18356	0.02446
14	0.89700	0.03142	0.17978	0.02312
15	0.83941	0.03096	0.17646	0.02187
16	0.78752	0.03147	0.17146	0.02064
17	0.73830	0.02926	0.16309	0.01933
18	0.69689	0.02885	0.15973	0.01808
19	0.66125	0.02729	0.15765	0.01685
20	0.62763	0.02669	0.14985	0.01576
21	0.59715	0.02622	0.14262	0.01457
22	0.57017	0.02665	0.13455	0.01345
23	0.54218	0.02471	0.12494	0.01218
24	0.51865	0.02416	0.11668	0.01099
25	0.49744	0.02568	0.10737	0.00983
26	0.47559	0.02486	0.08789	0.00897
27	0.45835	0.02562	0.08825	0.00765
28	0.44086	0.02497	0.07794	0.00655
29	0.42657	0.02306	0.07816	0.00563
30	0.41177	0.02134	0.07343	0.00469
31	0.38278	0.02250	0.04052	0.00380
32	0.38683	0.02191	0.06632	0.00332
33	0.37514	0.01846	0.07440	0.00272
34	0.36432	0.02179	0.06728	0.00227
35	0.35456	0.01734	0.06664	0.00187
36	0.34410	0.01856	0.06391	0.00155
37	0.33495	0.01823	0.06148	0.00128
38	0.32615	0.01769	0.06936	0.00107
39	0.31849	0.01954	0.06042	0.00089
40	0.31074	0.01217	0.07704	0.00072
41	0.30351	0.01122	0.07154	0.00061
42	0.29628	0.01813	0.05742	0.00053
43	0.28999	0.02495	0.04029	0.00046
44	0.28374	0.01421	0.06627	0.00037
45	0.27931	0.01645	0.06539	0.00032
46	0.27213	0.01650	0.07120	0.00027
47	0.26692	0.02029	0.05918	0.00025

48	0.26041	0.02214	0.03862	0.00021
49	0.25647	0.01292	0.07252	0.00018
50	0.25068	0.00000	0.06540	0.00014
51	0.24295	0.00000	0.05159	0.00013
52	0.24167	0.00000	0.07388	0.00011
53	0.23828	0.00000	0.10205	0.00010
54	0.23259	0.00000	0.06764	0.00009
55	0.22973	0.00000	0.08804	0.00008

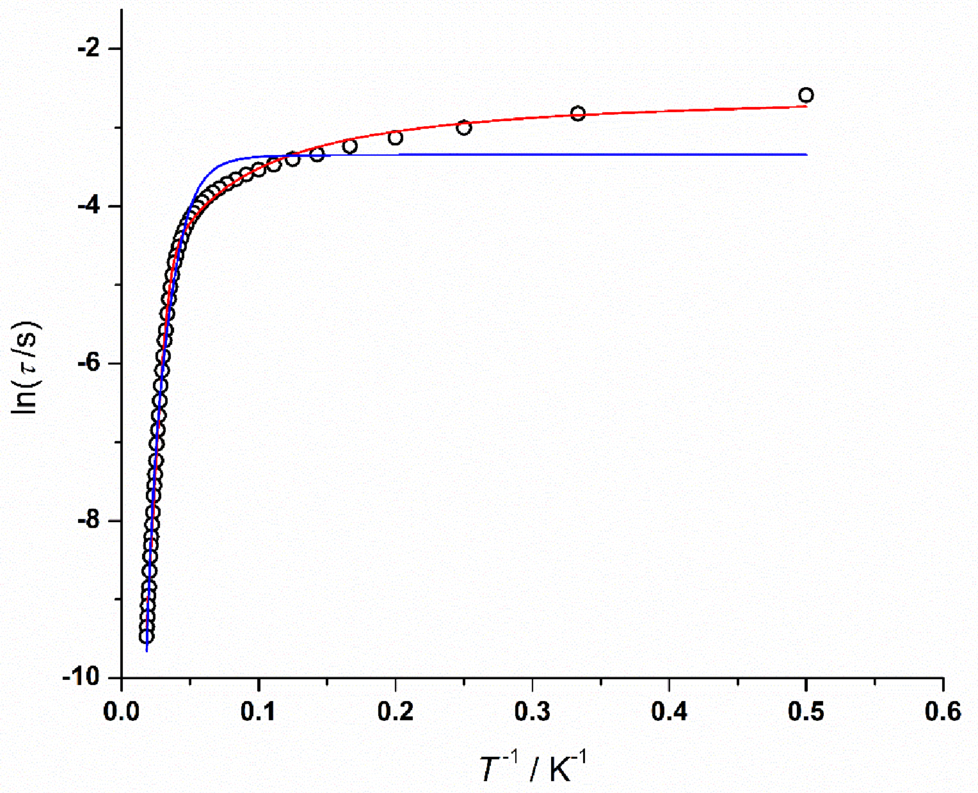

Figure S60. Plot of natural log of the relaxation time (τ) *vs.* inverse temperature for [**3**][K(18-crown-6)(THF)$_2$].

The red line is the best fit (adjusted R^2 = 0.99899) to $\tau^{-1} = \tau_0^{-1}e^{-U_{\text{eff}}/k_{\text{B}}T} + CT^n + \tau_{QTM}^{-1}$, giving: U_{eff} = 222(3) cm^{-1}, τ_0 = 2.69(3) × 10^{-7} s, C = 0.8(2) s^{-1} K^{-n}, n = 1.38(9) and τ_{QTM} = 0.076(7) s.

The blue line is the best fit (adjusted R^2 = 0.99013) to $\tau^{-1} = \tau_0^{-1}e^{-U_{\text{eff}}/k_{\text{B}}T} + CT^n + \tau_{QTM}^{-1}$, giving: U_{eff} (fixed) = 434 cm^{-1}, τ_0 = 1.2(2) × 10^{-9} s, C = 5(4) × 10^{-6} s^{-1} K^{-n}, n = 5.2(3) and τ_{QTM} = 0.035(2) s.

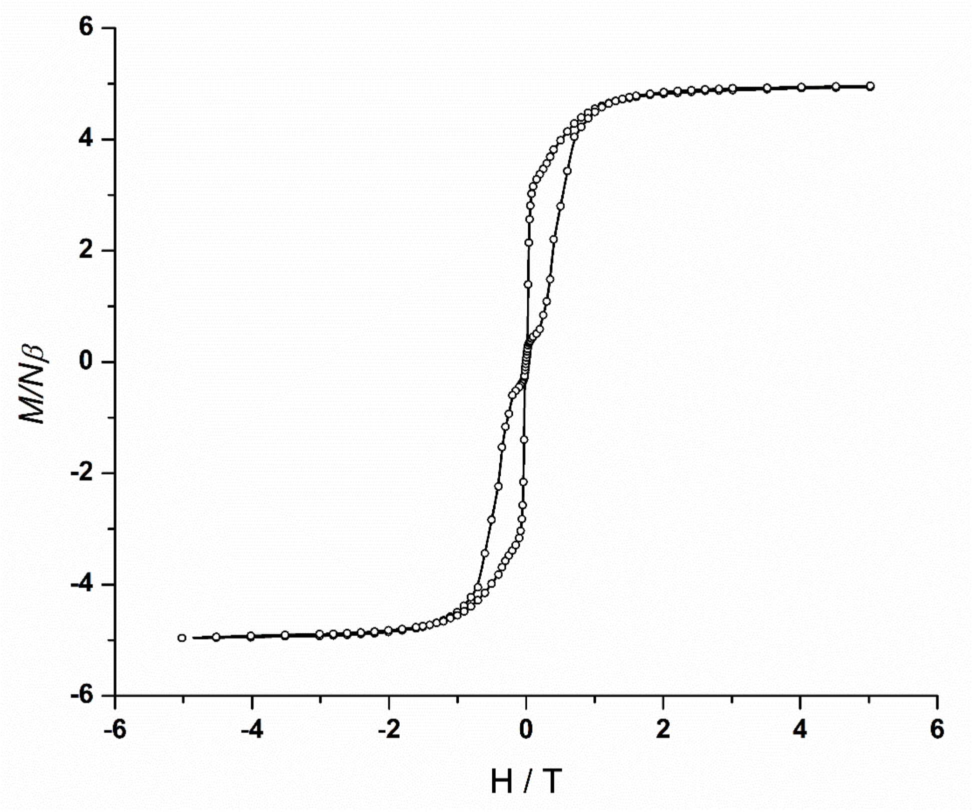

Figure S61. Magnetic hysteresis loops for [**3**][K(18-crown-6)(THF)$_2$]. The data were continuously collected at 1.9 K under a varying field sweep rate (1.1 mT s^{-1} |0-1| T, 3.0 mT s^{-1} |1-2| T, 4.5 mT s^{-1} |2-3| T and 8.5 mT s^{-1} |3-5| T). Solid lines are a guide to the eye.

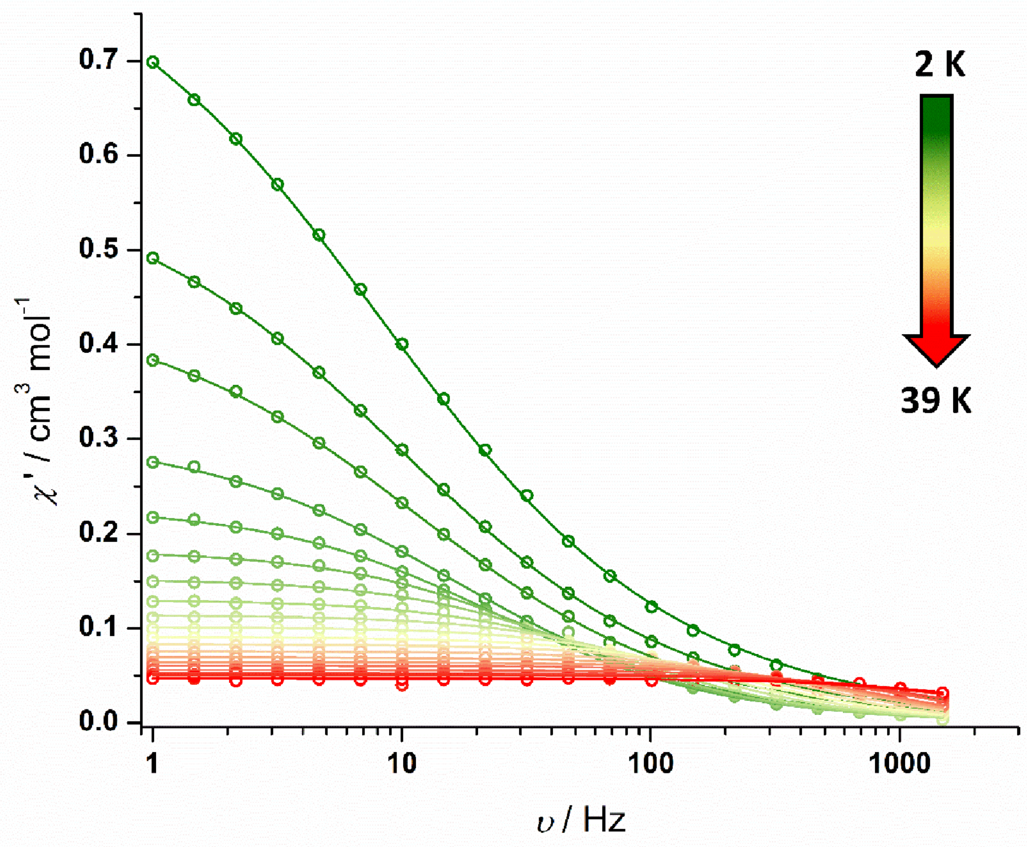

Figure S62. Frequency dependence of the in-phase susceptibility (χ') for [**1a**][Na(15-crown-5)(THF)$_2$] in zero DC field at ν = 1-1488 Hz and temperatures of 2-39 K. Solid lines represent fits to the data using equation 1.[2]

$$\chi'(\nu_{ac}) = \chi_\infty + \frac{(\chi_s - \chi_\infty)[1+(2\pi\nu_{ac}\tau)^{1-\alpha}\sin(\alpha\pi/2)]}{1+2(2\pi\nu_{ac}\tau)^{1-\alpha}\sin(\alpha\pi/2)+(2\pi\nu_{ac}\tau)^{2(1-\alpha)}} \quad \text{Equation 1}$$

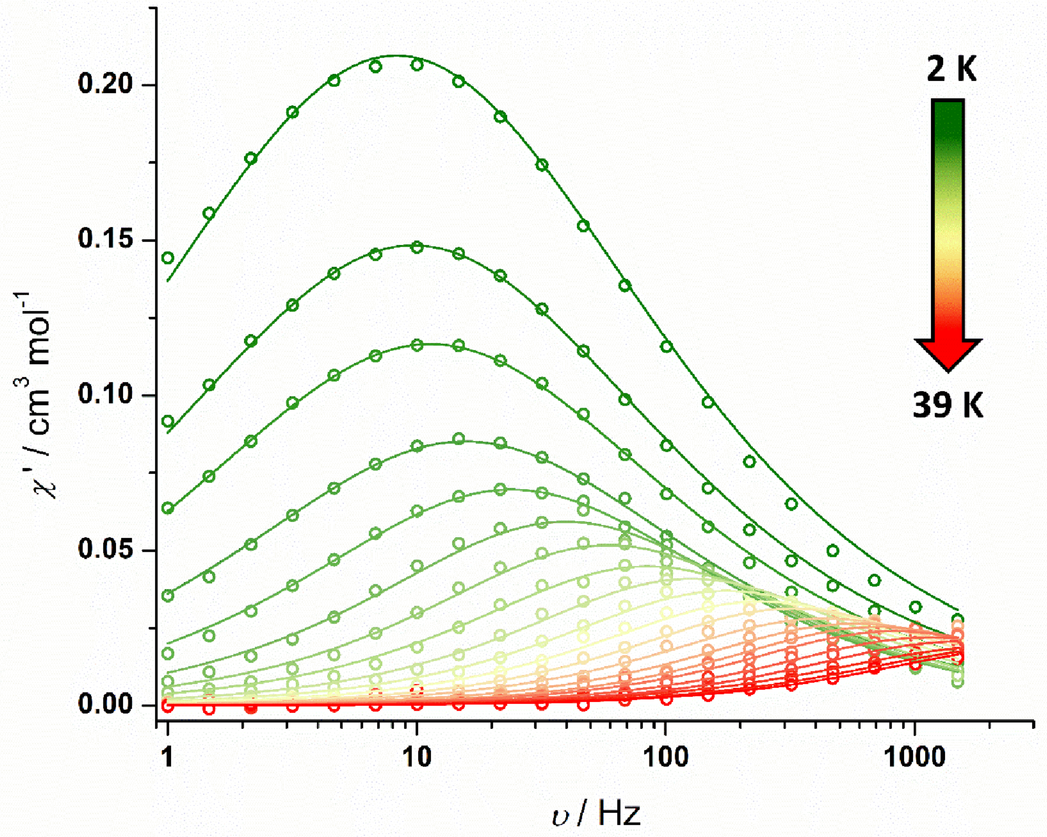

Figure S63. Frequency dependence of the out-of-phase susceptibility (χ'') for [**1a**][Na(15-crown-5)(THF)$_2$] in zero DC field at ν= 1-1488 Hz and temperatures of 2-39 K. Solid lines represent fits to the data using equation 2.[2]

$$\chi''(\nu_{ac}) = \frac{(\chi_s - \chi_\infty)(2\pi\nu_{ac}\tau)^{1-\alpha}\cos(\alpha\pi/2)}{1+2(2\pi\nu_{ac}\tau)^{1-\alpha}\sin(\alpha\pi/2)+(2\pi\nu_{ac}\tau)^{2(1-\alpha)}} \quad \text{Equation 2}$$

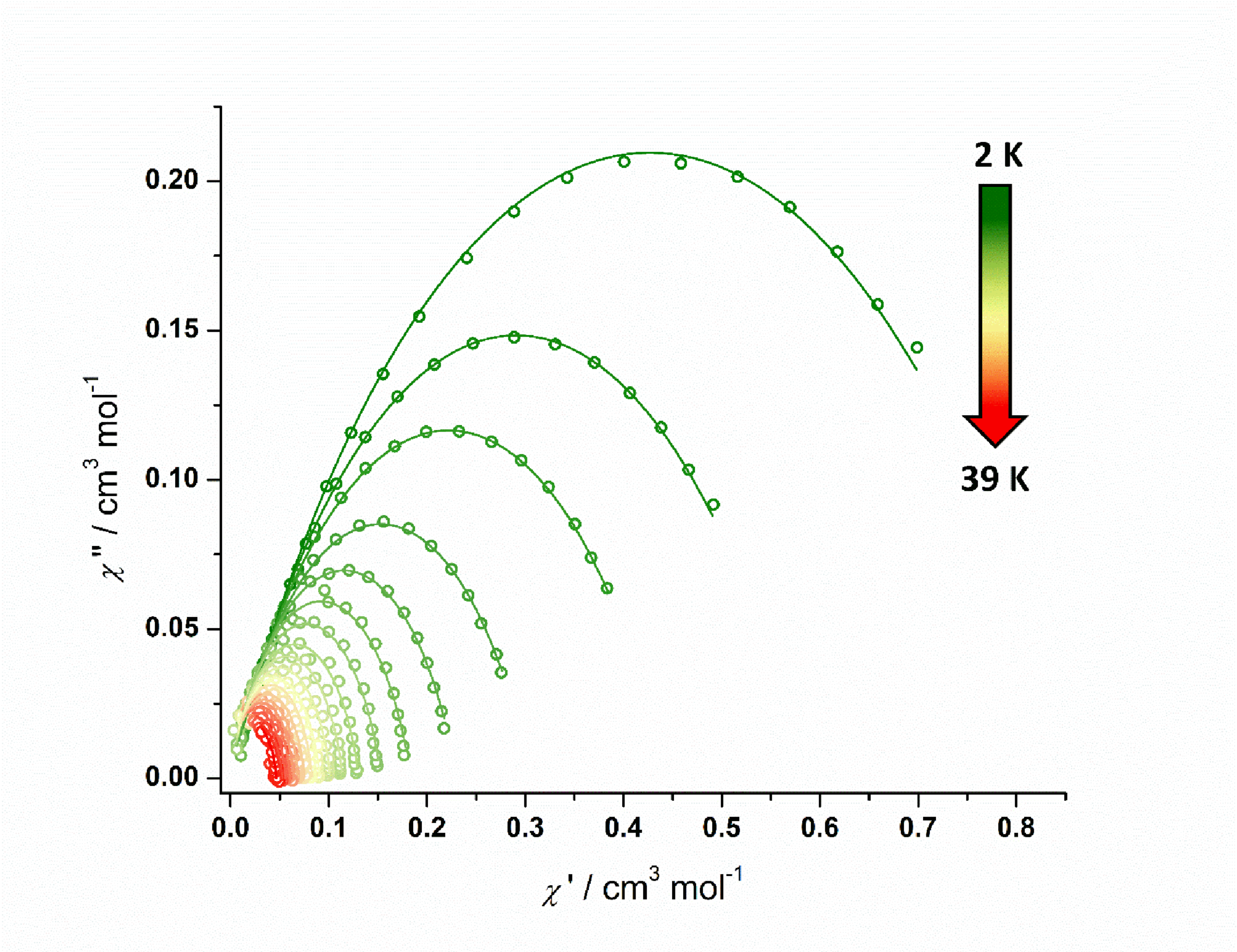

Figure S64. Cole-Cole plots for the AC susceptibilities in zero DC field for [**1a**][Na(15-crown-5)(THF)$_2$] from 2-39 K. Solid lines represent fits to the data using equations 1 and 2.

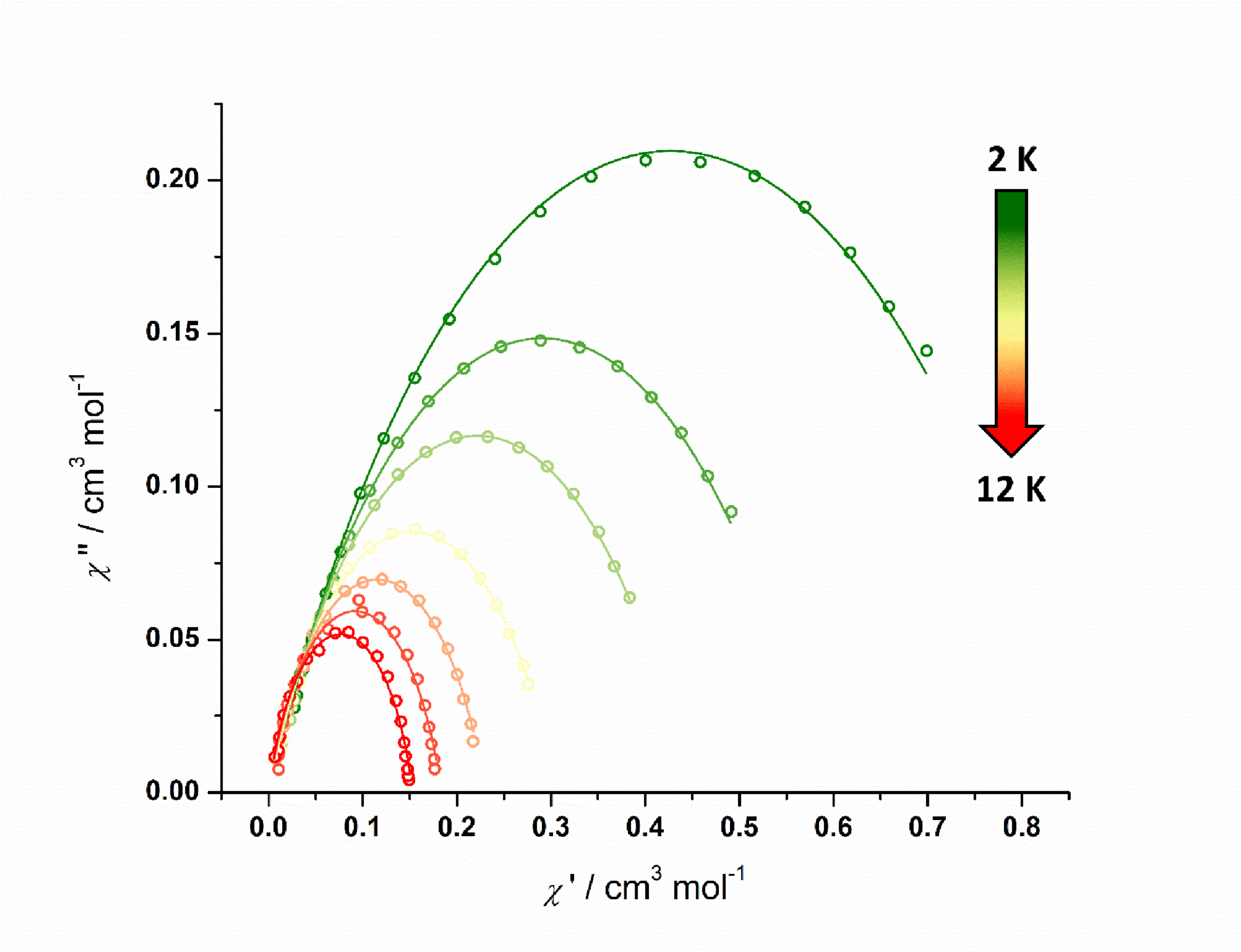

Figure S65. Cole-Cole plots for the AC susceptibilities in zero DC field for [**1a**][Na(15-crown-5)(THF)$_2$] from 2-12 K. Solid lines represent fits to the data using equations 1 and 2.

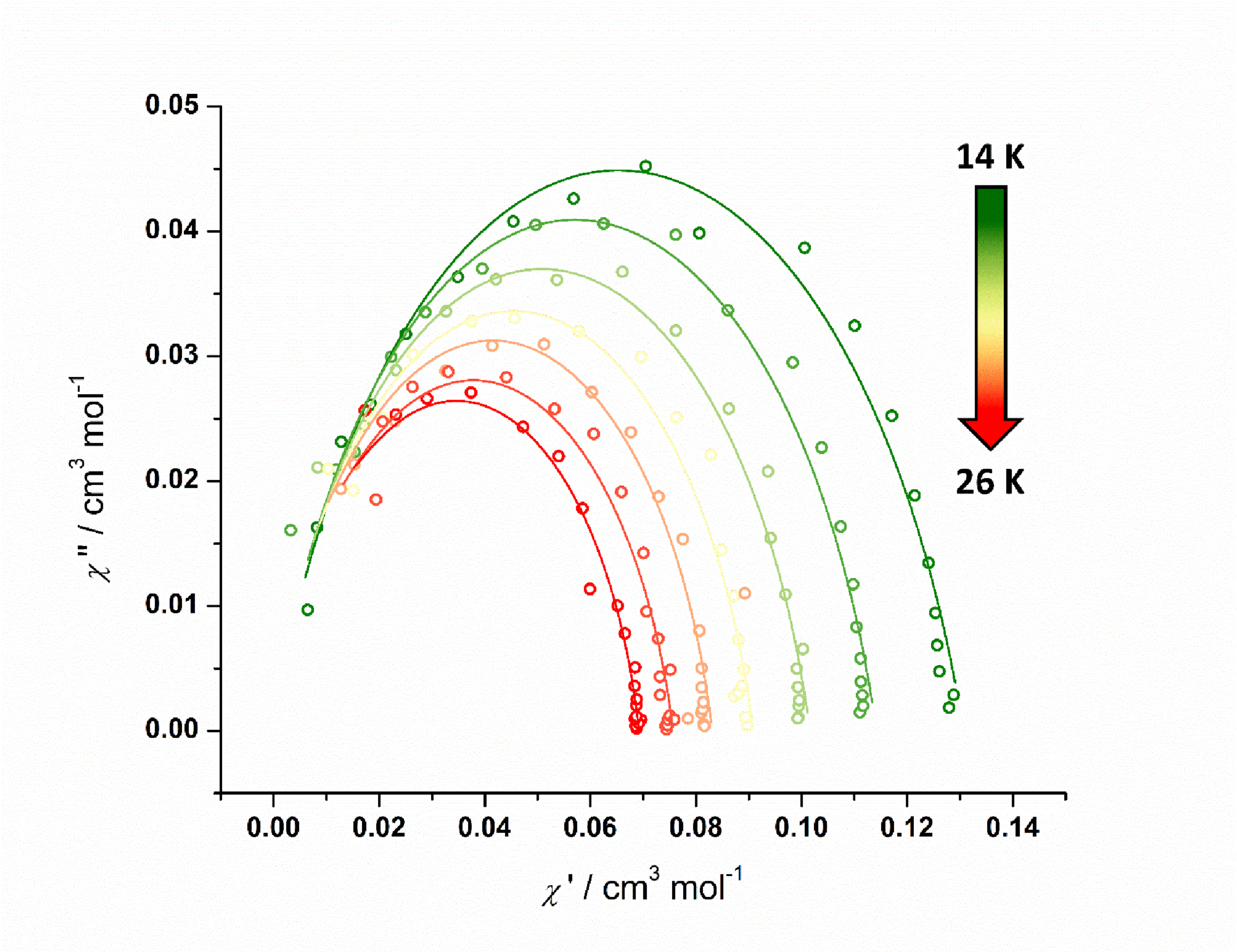

Figure S66. Cole-Cole plots for the AC susceptibilities in zero DC field for [**1a**][Na(15-crown-5)(THF)$_2$] from 14-26 K. Solid lines represent fits to the data using equations 1 and 2.

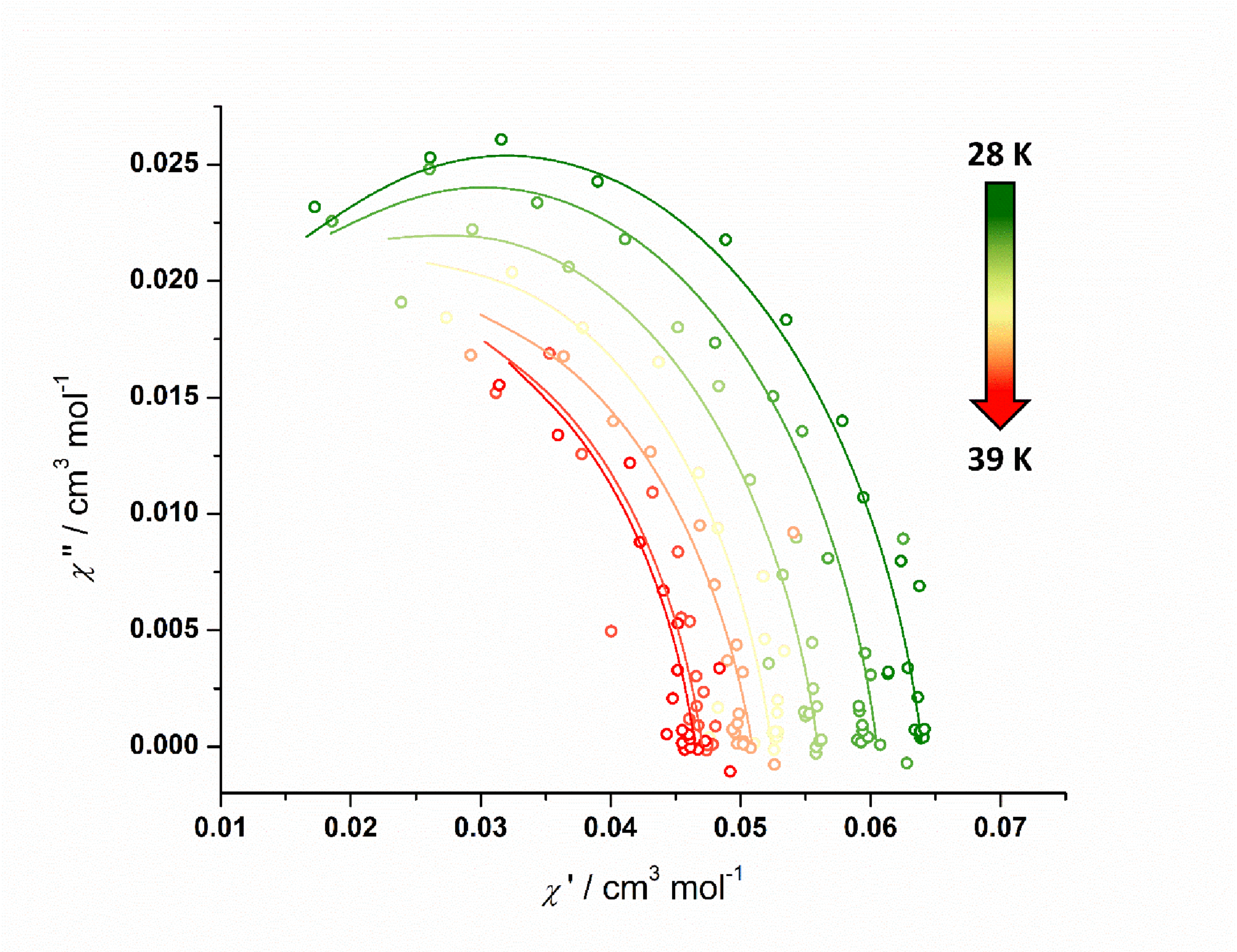

Figure S67. Cole-Cole plots for the AC susceptibilities in zero DC field for [**1a**][Na(15-crown-5)(THF)$_2$] from 28-39 K. Solid lines represent fits to the data using equations 1 and 2.

Table S7. Relaxation fitting parameters for [**1a**][Na(15-crown-5)(THF)$_2$] corresponding to Figures S64-S67.

T / K	χ_T / cm^3 mol^{-1}	χ_S / cm^3 mol^{-1}	α	τ / s
2	0.85346	5.55 x 10^{-15}	0.41618	0.01920
3	0.58117	9.08 x 10^{-15}	0.39604	0.01639
4	0.44367	1.86 x 10^{-14}	0.38097	0.01405
6	0.30503	3.45 x 10^{-14}	0.34789	0.01015
8	0.23017	6.82 x 10^{-14}	0.30248	0.00661
10	0.18356	1.31 x 10^{-13}	0.26453	0.00401
12	0.15310	1.39 x 10^{-13}	0.23703	0.00270
14	0.13066	2.49 x 10^{-13}	0.22726	0.00188
16	0.11415	5.15 x 10^{-13}	0.20116	0.00126
18	0.10158	8.12 x 10^{-13}	0.19155	0.00091
20	0.09082	9.99 x 10^{-13}	0.18162	0.00066
22	0.08306	1.66 x 10^{-12}	0.17084	0.00049
24	0.07568	2.43 x 10^{-12}	0.17984	0.00037
26	0.06920	4.28 x 10^{-12}	0.16138	0.00028
28	0.06397	5.81 x 10^{-12}	0.13707	0.00023
30	0.06049	8.32 x 10^{-12}	0.13644	0.00019
32	0.05594	1.37 x 10^{-11}	0.14400	0.00014
34	0.05239	1.82 x 10^{-11}	0.14605	0.00011
36	0.05092	2.58 x 10^{-11}	0.18531	0.00008
38	0.04706	3.64 x 10^{-11}	0.15633	0.00007
39	0.04651	5.43 x 10^{-11}	0.15424	0.00006

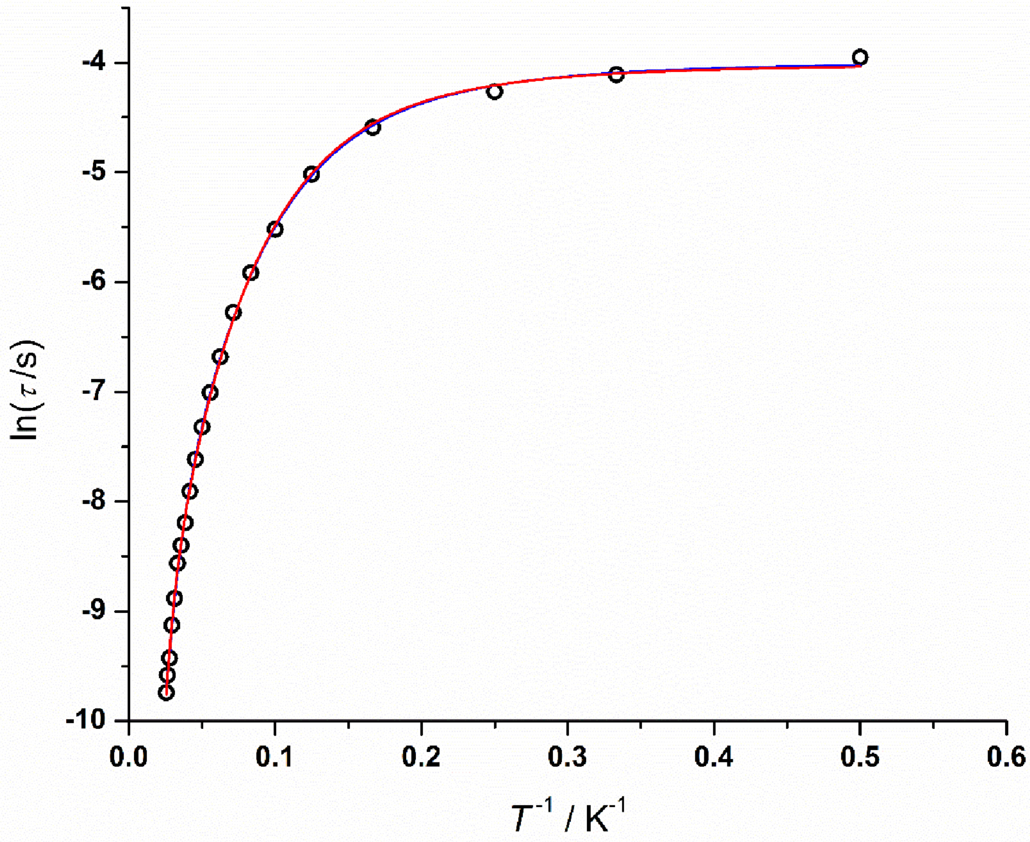

Figure S68. Plot of natural log of the relaxation time (τ) *vs.* inverse temperature for [**1a**][Na(15-crown-5)(THF)$_2$].

The red line is the best fit (adjusted R^2 = 0.99946) to $\tau^{-1} = \tau_0^{-1}e^{-U_{\mathrm{eff}}/k_{\mathrm{B}}T} + CT^n + \tau_{QTM}^{-1}$, giving: U_{eff} (fixed) = 242 cm^{-1}, τ_0 = 2.2(3) × 10^{-8} s, C = 0.18(2) s^{-1} K^{-n}, n = 3.01(4) and τ_{QTM} = 1.82(6) × 10^{-2} s.

The blue line is the best fit (adjusted R^2 = 0.99951) to $\tau^{-1} = \tau_0^{-1}e^{-U_{\mathrm{eff}}/k_{\mathrm{B}}T} + CT^n + \tau_{QTM}^{-1}$, giving: U_{eff} = 181(25) cm^{-1}, τ_0 = 2(2) × 10^{-7} s, C = 0.22(4) s^{-1} K^{-n}, n = 2.93(6) and τ_{QTM} = 1.84(6) × 10^{-2} s.

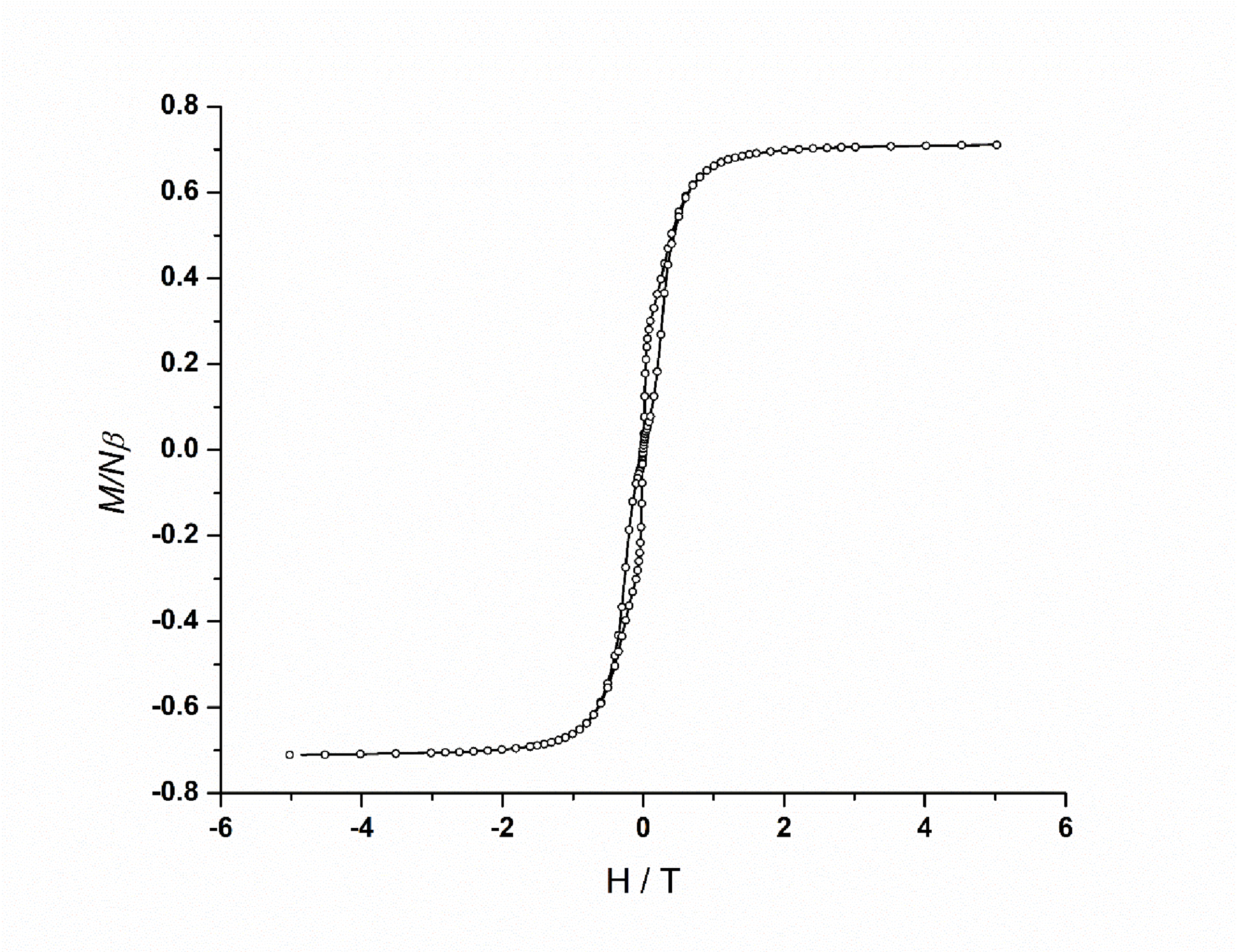

Figure S69. Magnetic hysteresis loops for [**1a**][Na(15-crown-5)(THF)$_2$]. The data were continuously collected at 1.9 K under a varying field sweep rate (1.1 mT s^{-1} |0-1| T, 3.0 mT s^{-1} |1-2| T, 4.5 mT s^{-1} |2-3| T and 8.5 mT s^{-1} |3-5| T). Solid lines are a guide to the eye.

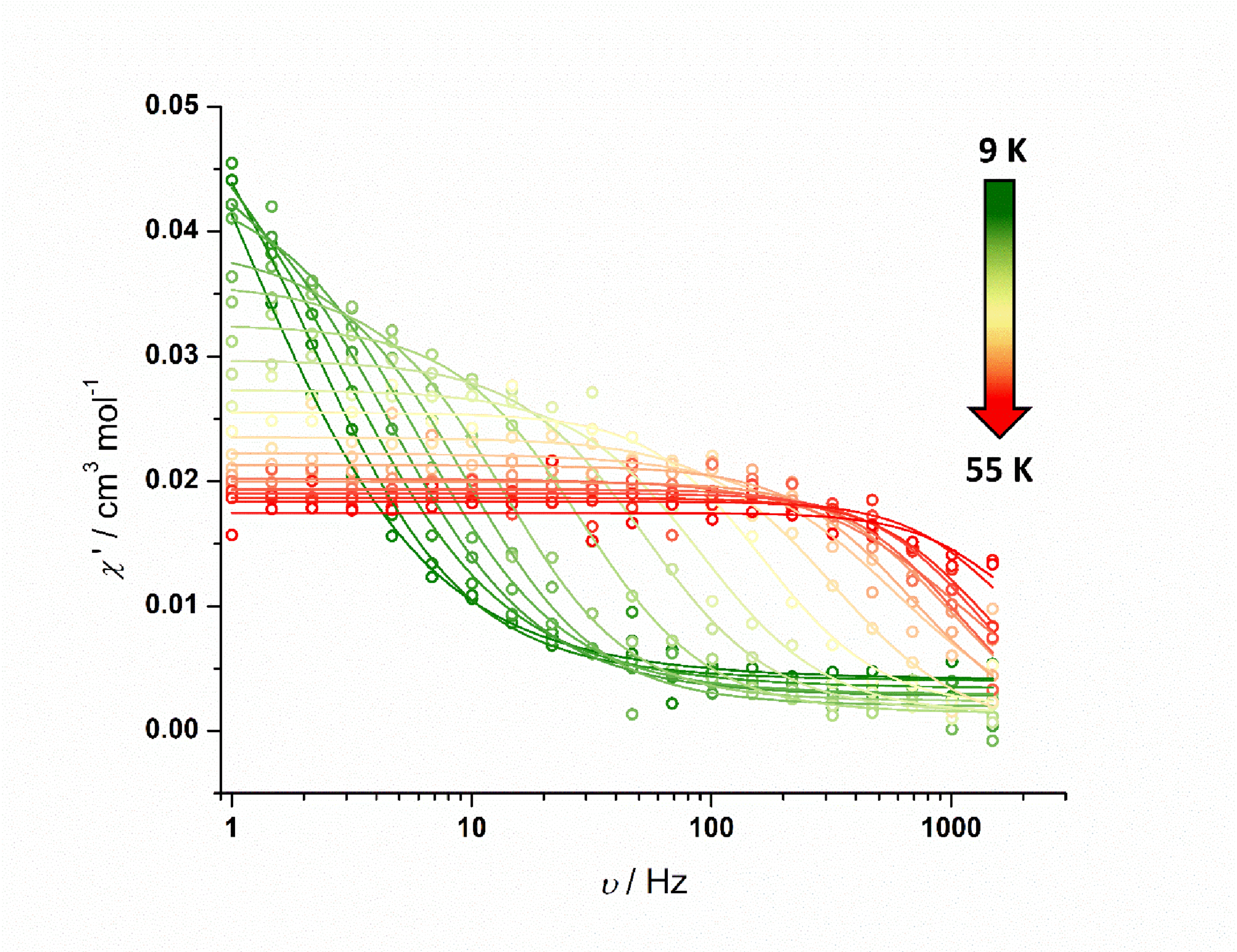

Figure S70. Frequency dependence of the in-phase susceptibility (χ') for [**3a**][K(18-crown-6)(THF)$_2$] in zero DC field at ν = 1-1488 Hz and temperatures of 9-55 K. Solid lines represent fits to the data using equation 1.[2]

$$\chi'(\nu_{ac}) = \chi_\infty + \frac{(\chi_S - \chi_\infty)[1 + (2\pi\nu_{ac}\tau)^{1-\alpha}\sin(\alpha\pi/2)]}{1 + 2(2\pi\nu_{ac}\tau)^{1-\alpha}\sin(\alpha\pi/2) + (2\pi\nu_{ac}\tau)^{2(1-\alpha)}} \qquad \text{Equation 1}$$

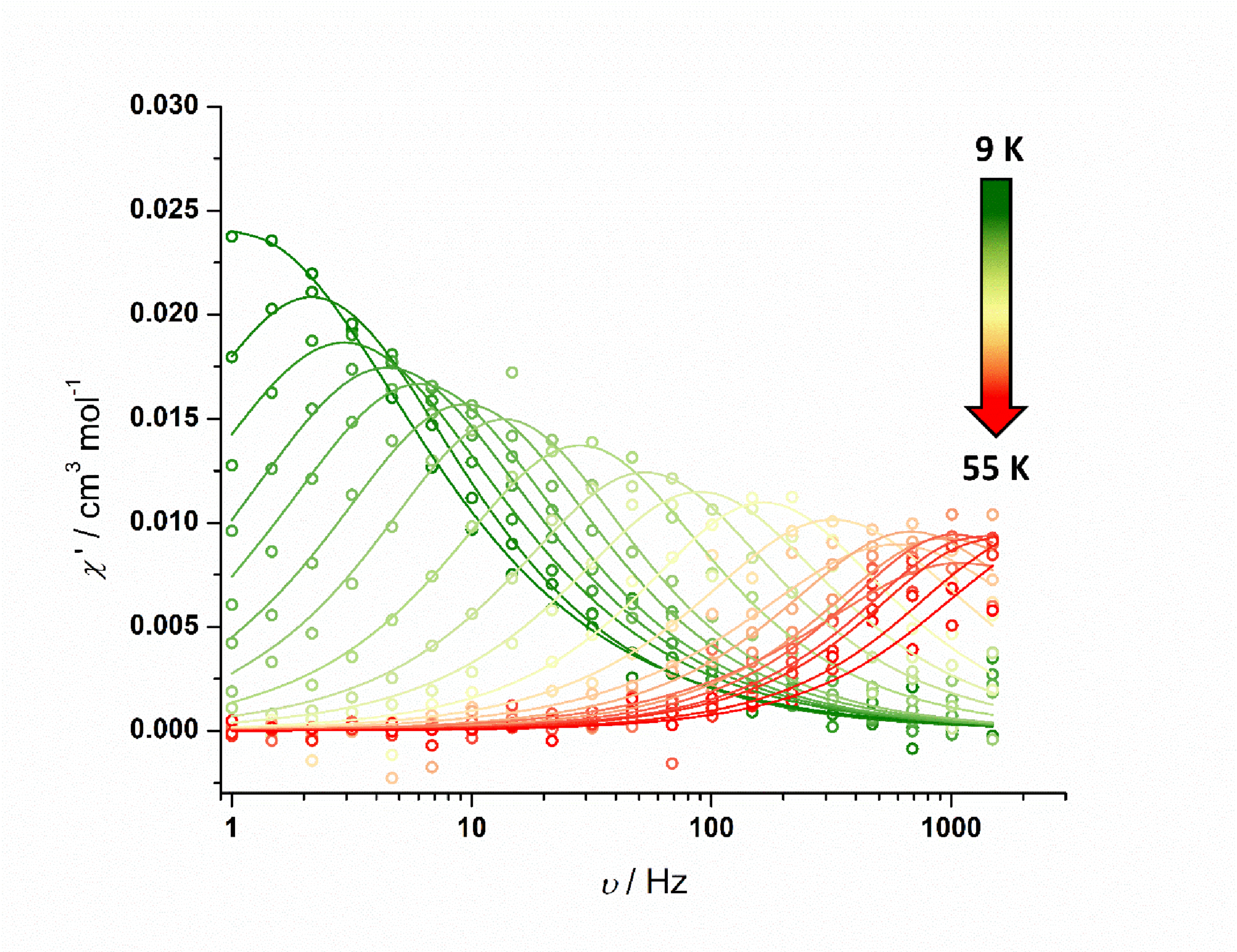

Figure S71. Frequency dependence of the out-of-phase susceptibility (χ'') for [**3a**][K(18-crown-6)(THF)$_2$] in zero DC field at ν = 1-1488 Hz and temperatures of 9-55 K. Solid lines represent fits to the data using equation 2.[2]

$$\chi''(\nu_{ac}) = \frac{(\chi_s - \chi_\infty)(2\pi\nu_{ac}\tau)^{1-\alpha}\cos(\alpha\pi/2)}{1+2(2\pi\nu_{ac}\tau)^{1-\alpha}\sin(\alpha\pi/2)+(2\pi\nu_{ac}\tau)^{2(1-\alpha)}}$$ Equation 2

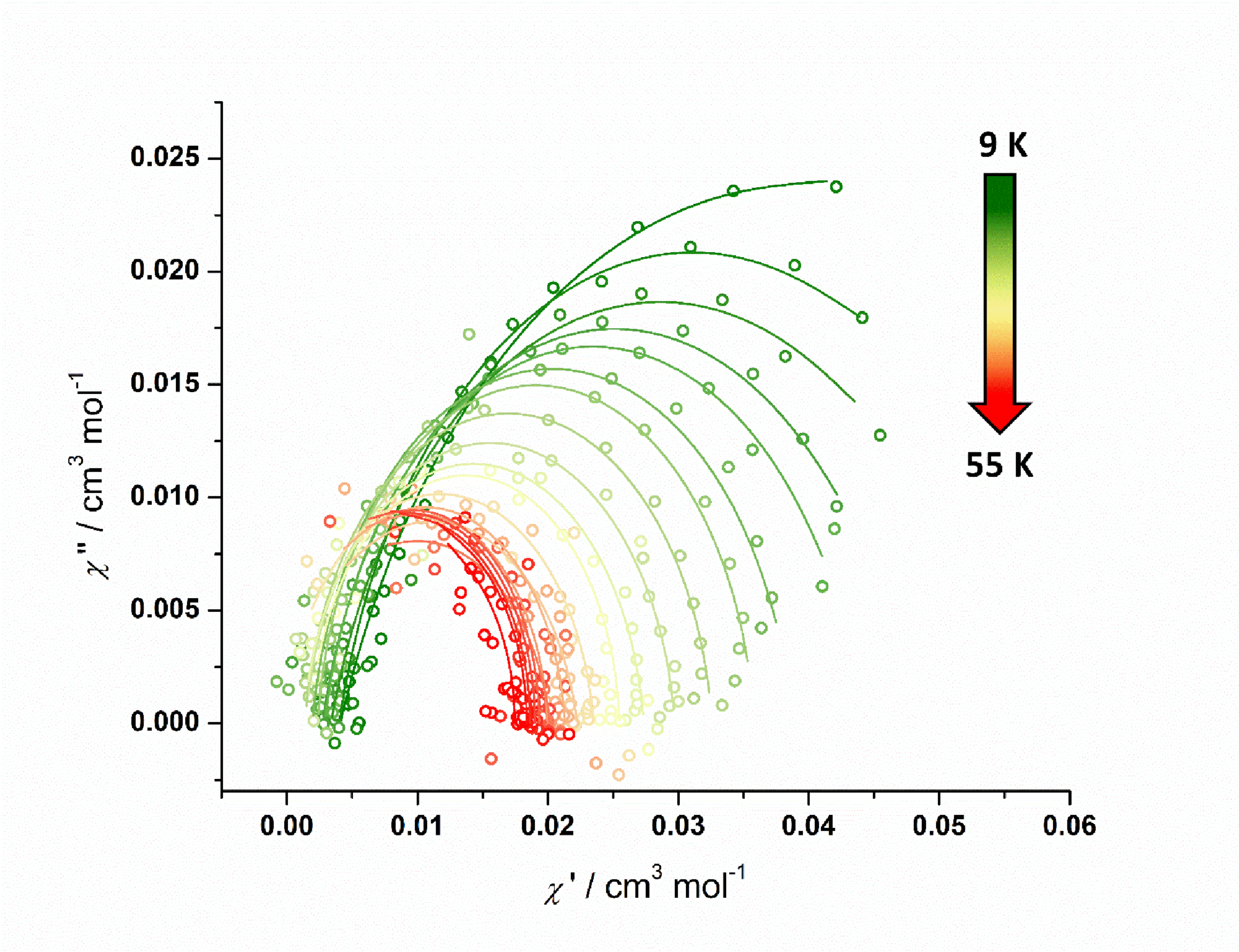

Figure S72. Cole-Cole plots for the AC susceptibilities in zero DC field for [**3a**][K(18-crown-6)(THF)$_2$] from 9-55 K. Solid lines represent fits to the data using equations 1 and 2.

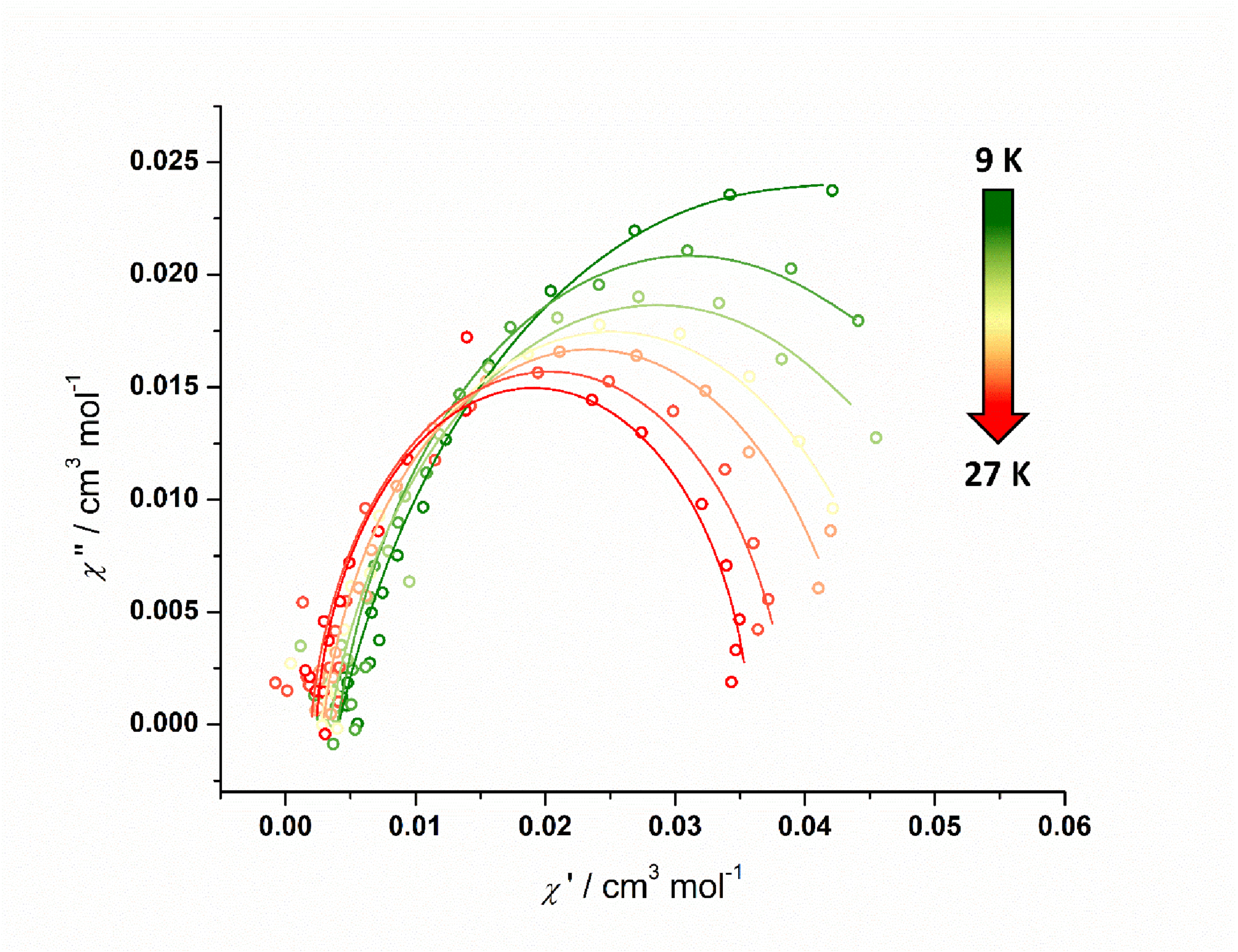

Figure S73. Cole-Cole plots for the AC susceptibilities in zero DC field for [**3a**][K(18-crown-6)(THF)$_2$] from 9-27 K. Solid lines represent fits to the data using equations 1 and 2.

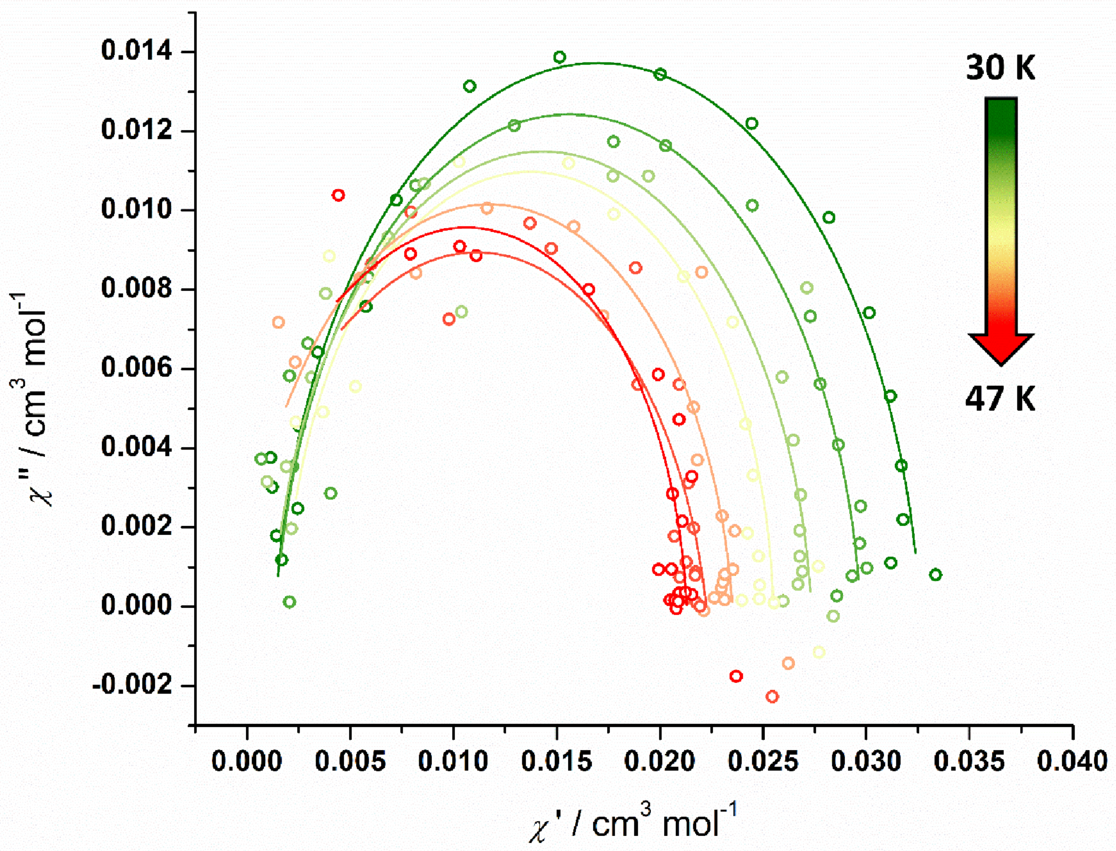

Figure S74. Cole-Cole plots for the AC susceptibilities in zero DC field for [**3a**][K(18-crown-6)(THF)$_2$] from 30-47 K. Solid lines represent fits to the data using equations 1 and 2.

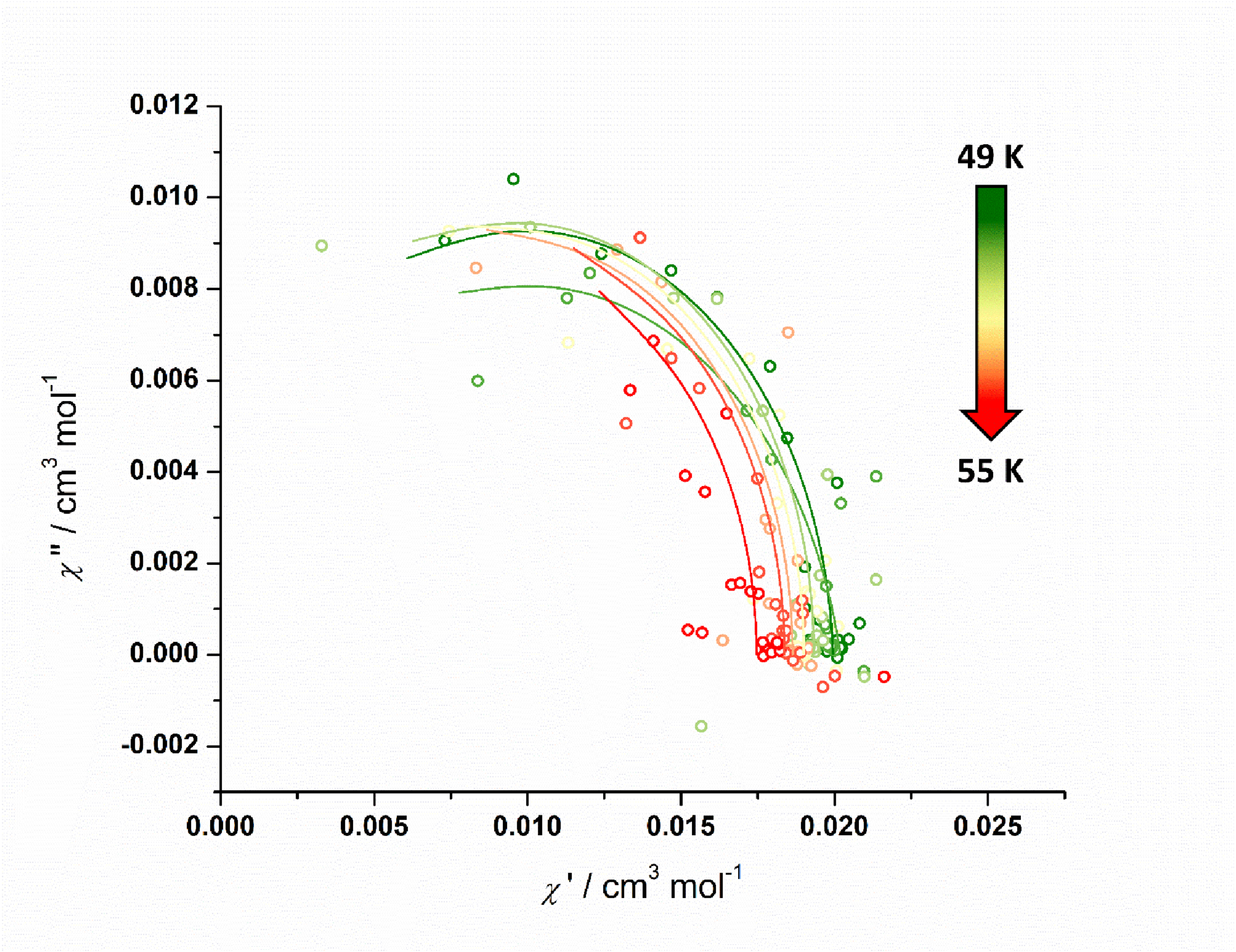

Figure S75. Cole-Cole plots for the AC susceptibilities in zero DC field for [**3a**][K(18-crown-6)(THF)$_2$] from 49-55 K. Solid lines represent fits to the data using equations 1 and 2.

Table S8. Relaxation fitting parameters for [**3a**][K(18-crown-6)(THF)$_2$] corresponding to Figures S72-S75.

T / K	χ_T / cm^3 mol^{-1}	χ_S / cm^3 mol^{-1}	α	τ / s
9	0.07464	4.09 x 10^{-3}	0.23796	0.14384
12	0.05813	4.01 x 10^{-3}	0.15582	0.07407
15	0.05382	3.39 x 10^{-3}	0.18150	0.05409
18	0.04750	2.73 x 10^{-3}	0.14775	0.03588
21	0.04402	2.89 x 10^{-3}	0.12282	0.02625
24	0.03871	1.96 x 10^{-3}	0.08905	0.01694
27	0.03579	2.39 x 10^{-3}	0.05724	0.01164
30	0.03258	1.39 x 10^{-3}	0.06957	0.00562
33	0.02972	1.45 x 10^{-3}	0.07017	0.00302
36	0.02733	1.29 x 10^{-3}	0.06746	0.00178
39	0.02549	1.91 x 10^{-3}	0.03209	0.00096
42	0.02351	1.65 x 10^{-9}	0.08154	0.00050
45	0.02224	1.82 x 10^{-9}	0.12825	0.00027
47	0.02129	3.02 x 10^{-9}	0.05541	0.00023
49	0.01996	1.82 x 10^{-9}	0.03369	0.00017
50	0.02019	4.36 x 10^{-11}	0.13278	0.00015
51	0.01933	4.93 x 10^{-11}	3.69 x 10^{-14}	0.00015
52	0.01900	6.62 x 10^{-11}	5.19 x 10^{-14}	0.00013
53	0.01864	9.88 x 10^{-11}	4.92 x 10^{-14}	0.00011
54	0.01837	1.45 x 10^{-10}	7.06 x 10^{-14}	0.00008
55	0.01746	1.99 x 10^{-10}	9.38 x 10^{-14}	0.00007

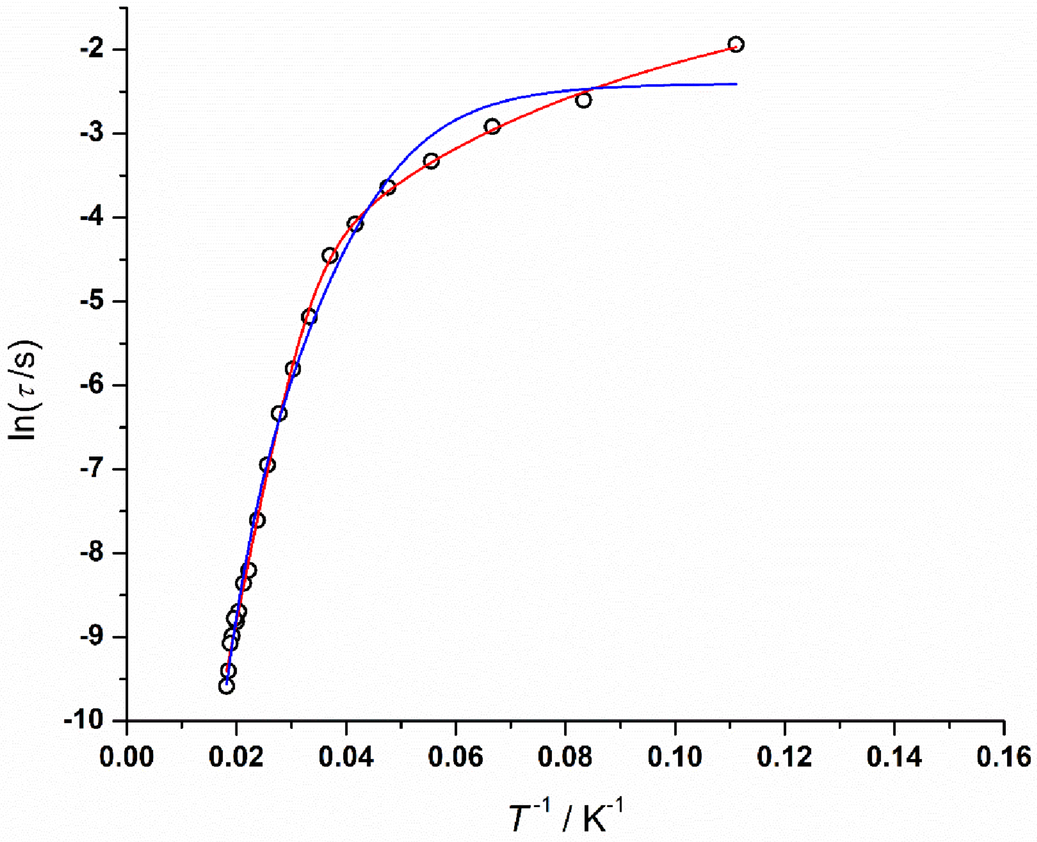

Figure S76. Plot of natural log of the relaxation time (τ) *vs.* inverse temperature for [**3a**][K(18-crown-6)(THF)$_2$].

The red line is the best fit (adjusted R^2 = 0.99863) to $\tau^{-1} = \tau_0^{-1}e^{-U_{\text{eff}}/k_{\text{B}}T} + CT^n + \tau_{QTM}^{-1}$, giving: U_{eff} = 233(8) cm^{-1}, τ_0 = 1.9(5) × 10^{-7} s, C = 0.04(5) s^{-1} K^{-n}, n = 2.3(4) and τ_{QTM} = 0.6(4) s.

The blue line is the best fit (adjusted R^2 = 0.99339) to $\tau^{-1} = \tau_0^{-1}e^{-U_{\text{eff}}/k_{\text{B}}T} + CT^n + \tau_{QTM}^{-1}$, giving: U_{eff} (fixed) = 434 cm^{-1}, τ_0 = 1.6(7) × 10^{-9} s, C = 4(5) × 10^{-7} s^{-1} K^{-n}, n = 5.9(4) and τ_{QTM} = 0.09(1) s.

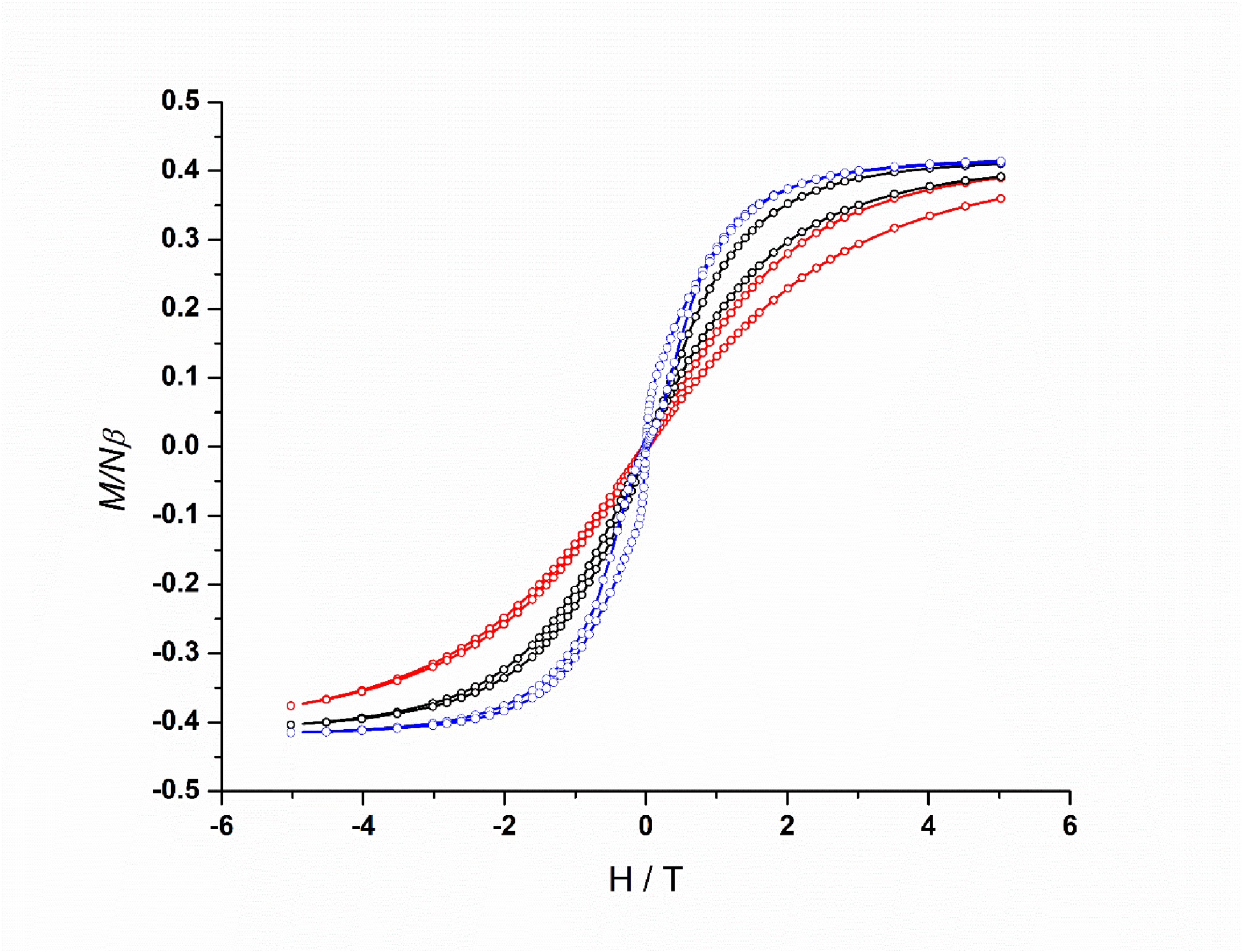

Figure S77. Magnetic hysteresis loops for [**3a**][K(18-crown-6)(THF)$_2$]. The data were continuously collected at either 2 K (blue circles), 3 K (black circles) or 5 K (red circles) under a varying field sweep rate (1.1 mT s^{-1} |0-1| T, 3.0 mT s^{-1} |1-2| T, 4.5 mT s^{-1} |2-3| T and 8.5 mT s^{-1} |3-5| T). Solid lines are a guide to the eye.

Computational Details

The geometries used in the calculations were extracted from the respective crystal structures. Non-coordinated counterions were removed from the structures. The positions of hydrogen atoms were optimized using density functional theory (DFT), while the positions of heavier atoms were frozen to their crystal-structure coordinates. The DFT calculations were carried out using the *ADF* 2019 code.[1] The pure PBE exchange-correlation functional[2] was used along with the empirical DFT-D3 dispersion correction[3] utilizing the Becke–Johnson damping function.[4] Scalar relativistic effects were treated using the zeroth-order regular approximation (ZORA) as implemented in *ADF*.[5] Valence-triple-ζ-quality Slater-type basis sets with two sets of polarization functions (TZ2P), specifically designed for ZORA calculations, were used for all atoms in the geometry optimizations.[6] Static electron correlation effects at the Dy ion were simulated by averaging the orbital occupations of the nine 4*f* electrons over the seven 4*f* orbitals. In practice this means that the occupations of the seven highest β orbitals were set to 0.285714, while the occupations of other orbitals were kept as integers. The "NumericalQuality" keyword in *ADF* was set to "Good" and the geometry convergence thresholds were increased to 10^{-4}, 10^{-4}, 10^{-3} and 10^{-1} atomic units for energy, energy gradient, bond length and bond angle, respectively.

The multireference calculations were carried out using the *OpenMolcas* 19.11 quantum chemistry code.[7] State-averaged complete active space self-consistent field (SA-CASSCF) calculations were first carried out.[8] The active space consisted of the nine 4*f* electrons in the seven 4*f* orbitals. All 21 sextet, 224 quartet and 490 doublet states were solved in three separate SA calculations. Spin-orbit coupling (SOC) was then taken into account using the spin-orbit restricted active space state interaction (SO-RASSI) methodology.[9] 21 sextet, 128 quartet and 130 doublet states, corresponding to an energy cut-off of 50,000 cm^{-1}, were included in the SO-RASSI treatment. The SOC operator was constructed using the atomic mean-field integral (AMFI) formalism[10] in the basis of the SA-CASSCF eigenstates and diagonalized to yield the final spin-orbit coupled eigenstates. The static magnetic properties (g-tensors, *ab initio* crystal-field parameters and transition magnetic moments) were calculated using the SINGLE_ANISO_OPEN module[11] in OpenMolcas. Scalar relativistic effects were treated using the scalar exact two-component (X2C) transformation.[12] Roos' relativistically contracted atomic natural orbital (ANO-RCC) basis sets were used throughout.[13] The Dy ions were treated using a VQZP basis. The H, B and C atoms in the conjugated skeletons of the Cb, Cp and Pn ligands and the coordinated $[BH_4]^-$ anions were treated using VTZP basis sets. The Si atoms were treated using a VTZP basis sets, whereas the C and H atoms in the substituent groups were treated using VDZP and VDZ basis sets, respectively. The K atom was treated using a VDZP basis set, whereas the remaining atoms were treated using a minimal basis. Cholesky decomposition with a threshold of 10^{-8} atomic units was used in storage of the two-electron integrals.

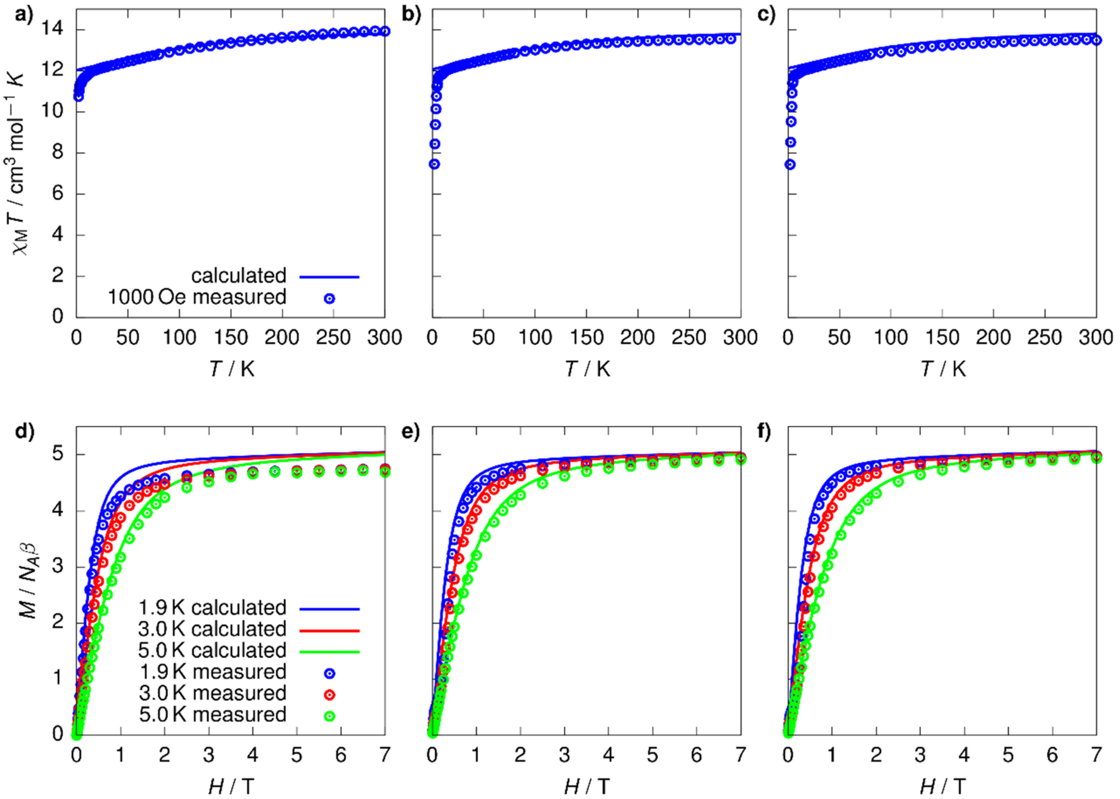

Figure S78. Comparison of the experimental and calculated $\chi_M T(T)$ and $M(H)$ data for **1** (a, d), **2** (b, e) and **3** (c, f) under the conditions indicated.

Table S9. Properties of the eight lowest KDs of **1** corresponding to the crystal-field split states of the ground $^6H_{15/2}$ multiplet.

**KD**	***E* / cm−1**	***gx***	***gy***	***gz***	**θ *a***
KD1	0	0.00892	0.01548	19.62434	0.0°
KD2	242	0.23432	0.37985	16.30245	8.4°
KD3	386	1.69921	1.91848	13.37681	36.9°
KD4	486	2.64726	4.99014	8.97824	30.8°
KD5	580	3.73311	4.59844	9.54713	88.4°
KD6	675	1.14778	1.51743	13.79530	89.8°
KD7	821	0.07935	0.11918	17.03248	90.1°
KD8	1142	0.00149	0.00240	19.78590	90.0°

a The angle between the principal magnetic axis of the given doublet and the that of the ground doublet.

Table S10. Properties of the eight lowest KDs of **2** corresponding to the crystal-field split states of the ground $^6H_{15/2}$ multiplet.

**KD**	***E* / cm−1**	***gx***	***gy***	***gz***	**θ *a***
KD1	0	0.00065	0.00095	19.65219	0.0°
KD2	236	0.00877	0.00958	16.82059	1.0°
KD3	447	0.06025	0.06905	14.36307	2.0°
KD4	602	0.42376	0.50981	11.66440	3.4°
KD5	719	2.41778	2.65534	8.29950	8.1°
KD6	800	3.60593	5.76957	10.08113	88.7°
KD7	892	0.48072	1.11393	15.79259	90.8°
KD8	1162	0.00285	0.00492	19.62304	89.9°

a The angle between the principal magnetic axis of the given doublet and the that of the ground doublet.

Table S11. Properties of the eight lowest KDs of **3** corresponding to the crystal-field split states of the ground $^6H_{15/2}$ multiplet.

**KD**	***E* / cm−1**	***gx***	***gy***	***gz***	**θ *a***
KD1	0	0.00049	0.00072	19.69399	0.0°
KD2	228	0.00591	0.00597	16.95159	2.2°
KD3	434	0.03664	0.04400	14.50732	4.2°
KD4	593	0.21194	0.27	11.78959	2.8°
KD5	722	1.17392	1.42428	8.78852	6.6°
KD6	816	6.98066	6.02358	4.49167	16.0°
KD7	897	1.06355	2.90099	14.22865	92.4°
KD8	1100	0.01334	0.02608	19.35561	89.8°

a The angle between the principal magnetic axis of the given doublet and the that of the ground doublet.

Table S12. *Ab initio* CF parameters (in cm^{-1}) calculated for **1** given in the Iwahara-Chibotaru notation.[22]

k	*q*	**Re(*Bkq*)**	**Im(*Bkq*)**	**|*Bkq*|**
2	0	−488.628052	0.000000	488.628052
2	1	−1.770343	−26.114585	26.174523
2	2	248.968094	−8.533045	249.114281
4	0	−18.877515	−0.000000	18.877515
4	1	0.974853	17.193406	17.221020
4	2	−9.848078	−0.884634	9.887730
4	3	2.788306	9.903302	10.288345
4	4	10.711146	−4.144374	11.484968
6	0	−15.420268	−0.000000	15.420268
6	1	0.873194	−1.712827	1.922562
6	2	20.605365	0.991802	20.629220
6	3	1.668957	−9.564200	9.708725
6	4	−7.108821	−0.216820	7.112127
6	5	0.400810	−3.513403	3.536191
6	6	9.189532	−0.112310	9.190219
8	0	0.771366	0.000000	0.771366
8	1	−0.038032	−0.183194	0.187100
8	2	−0.773408	−0.012016	0.773501
8	3	−0.052150	0.318554	0.322794
8	4	−0.003933	−0.009346	0.010140
8	5	−0.007086	0.021004	0.022167
8	6	−0.067805	0.004848	0.067979
8	7	0.003012	−0.019040	0.019277
8	8	0.037793	−0.004706	0.038085
10	0	0.001493	−0.000000	0.001493
10	1	−0.000290	0.023759	0.023761
10	2	−0.000019	−0.000693	0.000693
10	3	−0.002309	0.004601	0.005148
10	4	0.002866	0.001061	0.003057
10	5	−0.001251	0.008620	0.008710
10	6	−0.002040	−0.000177	0.002048
10	7	−0.000152	−0.006268	0.006270
10	8	0.005415	0.000487	0.005437
10	9	0.000632	−0.001791	0.001900
10	10	0.001658	−0.001210	0.002053
12	0	0.004163	0.000000	0.004163
12	1	−0.000266	−0.001727	0.001748
12	2	−0.002904	0.000020	0.002904

12	3	−0.000035	0.000795	0.000795
12	4	0.001609	0.000088	0.001611
12	5	0.000199	−0.000832	0.000855
12	6	−0.000495	−0.000045	0.000497
12	7	−0.000050	0.000243	0.000248
12	8	−0.000151	−0.000031	0.000154
12	9	−0.000016	0.000098	0.000100
12	10	−0.000263	−0.000008	0.000263
12	11	0.000006	−0.000109	0.000109
12	12	0.000275	−0.000008	0.000275
14	0	−0.000022	−0.000000	0.000022
14	1	0.000001	0.000000	0.000001
14	2	0.000016	−0.000001	0.000016
14	3	0.000002	0.000002	0.000002
14	4	−0.000008	−0.000001	0.000008
14	5	−0.000001	−0.000001	0.000001
14	6	0.000005	0.000000	0.000005
14	7	0.000000	0.000001	0.000001
14	8	−0.000000	−0.000000	0.000000
14	9	−0.000000	0.000000	0.000000
14	10	0.000001	0.000000	0.000001
14	11	−0.000000	0.000000	0.000000
14	12	−0.000000	−0.000000	0.000000
14	13	0.000000	−0.000000	0.000000
14	14	0.000000	0.000000	0.000000

a The CF parameters are only listed for non-negative values of *q*. The values with negative *q* are given by *Bk−q* = (−1)*qBkq**.

Table S13. *Ab initio* CF parameters (in cm^{-1}) calculated for **2** given in the Iwahara-Chibotaru notation.[22]

k	*q*	Re(*Bkq*)	Im(*Bkq*)	|*Bkq*|
2	0	−598.848671	−0.000000	598.848671
2	1	6.099383	−2.065023	6.439471
2	2	157.784173	−4.200693	157.840080
4	0	20.038778	0.000000	20.038778
4	1	−0.027160	3.717197	3.717296
4	2	15.663675	0.018395	15.663686
4	3	−1.438030	0.066054	1.439546
4	4	17.226368	−11.056282	20.469224
6	0	−10.133563	0.000000	10.133563
6	1	−1.685848	−2.056558	2.659232
6	2	19.148669	0.389814	19.152637
6	3	1.237093	−0.171646	1.248944
6	4	−15.146470	4.364466	15.762745
6	5	−0.367230	−0.971369	1.038468
6	6	2.487988	0.286683	2.504451
8	0	0.491596	−0.000000	0.491596
8	1	0.074040	0.039969	0.084139
8	2	−0.702026	0.016362	0.702217
8	3	−0.008526	0.061605	0.062192
8	4	0.297566	−0.120664	0.321100
8	5	0.004751	0.013286	0.014109
8	6	−0.093150	0.016564	0.094611
8	7	−0.000960	−0.004616	0.004715
8	8	0.005773	−0.004927	0.007590
10	0	0.025381	0.000000	0.025381
10	1	−0.000739	0.000785	0.001078
10	2	0.002926	−0.000518	0.002972
10	3	0.000618	−0.002780	0.002848
10	4	0.001464	0.002493	0.002892
10	5	0.002070	0.001131	0.002359
10	6	−0.008608	0.002444	0.008949
10	7	0.000558	−0.001492	0.001593
10	8	−0.003962	0.006133	0.007301
10	9	−0.000558	−0.000504	0.000751
10	10	0.000771	−0.000574	0.000961
12	0	0.004190	−0.000000	0.004190
12	1	0.000812	−0.000517	0.000962
12	2	−0.002293	0.000365	0.002322

12	3	−0.000290	0.000285	0.000407
12	4	0.001333	−0.000161	0.001342
12	5	−0.000022	−0.000147	0.000148
12	6	−0.000393	0.000113	0.000409
12	7	−0.000061	0.000062	0.000087
12	8	0.000435	−0.000348	0.000557
12	9	0.000044	0.000059	0.000073
12	10	−0.000108	0.000029	0.000112
12	11	−0.000004	−0.000012	0.000012
12	12	0.000024	0.000005	0.000025
14	0	−0.000020	−0.000000	0.000020
14	1	−0.000005	0.000000	0.000005
14	2	0.000016	−0.000002	0.000016
14	3	0.000000	0.000001	0.000001
14	4	−0.000008	0.000001	0.000008
14	5	−0.000001	0.000000	0.000001
14	6	0.000005	−0.000002	0.000006
14	7	−0.000000	−0.000000	0.000000
14	8	−0.000000	0.000000	0.000001
14	9	0.000000	−0.000000	0.000000
14	10	−0.000000	0.000001	0.000001
14	11	0.000000	−0.000000	0.000000
14	12	−0.000000	0.000000	0.000001
14	13	−0.000000	−0.000000	0.000000
14	14	−0.000000	0.000000	0.000000

a The CF parameters are only listed for non-negative values of *q*. The values with negative *q* are given by *Bk−q* = (−1)*qBkq**.

Table S14. *Ab initio* CF parameters (in cm^{-1}) calculated for **3** given in the Iwahara-Chibotaru notation.[22]

k	*q*	Re(*Bkq*)	Im(*Bkq*)	|*Bkq*|
2	0	−602.759450	−0.000000	602.759450
2	1	−14.624570	1.786592	14.733295
2	2	115.142108	−6.743509	115.339412
4	0	28.003772	0.000000	28.003772
4	1	0.941639	2.197372	2.390634
4	2	15.883818	−1.183679	15.927862
4	3	2.194066	6.045896	6.431701
4	4	3.428588	−5.773983	6.715214
6	0	−8.845629	−0.000000	8.845629
6	1	2.966482	−2.714969	4.021327
6	2	19.575301	1.564558	19.637726
6	3	−5.022562	−1.624568	5.278764
6	4	−11.132759	1.112996	11.188257
6	5	−0.301582	−0.106896	0.319967
6	6	2.298968	0.083095	2.300469
8	0	0.380161	0.000000	0.380161
8	1	−0.114972	0.069175	0.134178
8	2	−0.668717	−0.030083	0.669393
8	3	0.123283	0.096387	0.156490
8	4	0.208807	−0.038502	0.212327
8	5	0.006196	−0.003587	0.007159
8	6	−0.068204	0.006675	0.068530
8	7	−0.002586	−0.004553	0.005236
8	8	−0.001971	−0.002936	0.003536
10	0	0.023100	−0.000000	0.023100
10	1	−0.001548	0.000033	0.001548
10	2	0.009260	−0.001715	0.009418
10	3	−0.005910	−0.001898	0.006207
10	4	0.000849	0.001104	0.001392
10	5	−0.002281	0.002566	0.003434
10	6	−0.006417	−0.000171	0.006419
10	7	−0.001652	−0.001906	0.002522
10	8	0.000176	0.001687	0.001696
10	9	−0.000312	0.000163	0.000352
10	10	−0.000235	−0.000399	0.000463
12	0	0.004158	0.000000	0.004158
12	1	−0.001702	−0.000906	0.001928
12	2	−0.001929	0.000382	0.001966

12	3	0.000785	0.000249	0.000823
12	4	0.001198	0.000030	0.001199
12	5	−0.000259	−0.000374	0.000454
12	6	−0.000226	0.000065	0.000235
12	7	0.000205	0.000076	0.000219
12	8	0.000230	−0.000067	0.000240
12	9	0.000012	−0.000001	0.000012
12	10	−0.000066	0.000007	0.000066
12	11	−0.000001	−0.000000	0.000001
12	12	0.000018	0.000001	0.000018
14	0	−0.000018	−0.000000	0.000018
14	1	0.000008	0.000001	0.000008
14	2	0.000013	−0.000000	0.000013
14	3	−0.000006	0.000001	0.000006
14	4	−0.000008	−0.000000	0.000008
14	5	0.000004	0.000001	0.000004
14	6	0.000003	−0.000001	0.000003
14	7	0.000000	−0.000000	0.000000
14	8	−0.000000	0.000000	0.000000
14	9	−0.000000	−0.000000	0.000000
14	10	0.000000	0.000000	0.000000
14	11	−0.000000	−0.000000	0.000000
14	12	−0.000000	0.000000	0.000000
14	13	−0.000000	−0.000000	0.000000
14	14	0.000000	0.000000	0.000000

a The CF parameters are only listed for non-negative values of *q*. The values with negative *q* are given by *Bk−q* = (−1)*qBkq**.

Table S15. *Ab initio* CF parameters (in cm^{-1}) calculated for **4** given in the Iwahara-Chibotaru notation.[22]

K	q	Re(B_{kq})	Im(B_{kq})	$\|B_{kq}\|$
2	0	–452.849182	0.000000	452.849182
2	1	–4.901812	17.931554	18.589470
2	2	249.902621	0.512218	249.903146
4	0	–31.139485	0.000000	31.139485
4	1	3.519068	–8.690605	9.376057
4	2	–13.484925	2.031378	13.637071
4	3	8.744463	–5.606551	10.387446
4	4	0.589446	0.230511	0.632915
6	0	–26.536591	0.000000	26.536591
6	1	1.171412	–3.678629	3.860637
6	2	18.417542	1.625791	18.489161
6	3	2.145201	0.508272	2.204592
6	4	–0.511454	1.960035	2.025665
6	5	1.673191	2.063102	2.656306
6	6	13.261329	–0.455491	13.269150
8	0	1.008508	0.000000	1.008508
8	1	–0.068769	0.078357	0.104254
8	2	–0.629652	–0.014232	0.629813
8	3	–0.058093	0.078469	0.097633
8	4	–0.112857	–0.037945	0.119065
8	5	–0.014431	–0.001525	0.014511
8	6	–0.035694	0.008319	0.036651
8	7	0.009347	0.016353	0.018836
8	8	0.064795	–0.002348	0.064837
10	0	–0.003646	0.000000	0.003646
10	1	–0.001295	0.004492	0.004675
10	2	–0.011026	–0.006425	0.012762
10	3	–0.004545	0.000720	0.004602
10	4	–0.000301	–0.002750	0.002766
10	5	–0.001426	–0.004501	0.004721
10	6	–0.009111	–0.000425	0.009121
10	7	0.000626	–0.000270	0.000682
10	8	0.007280	0.000794	0.007323
10	9	0.003045	–0.002038	0.003664
10	10	–0.001046	0.000632	0.001222
12	0	0.004928	0.000000	0.004928
12	1	–0.000314	–0.000275	0.000417
12	2	–0.003409	–0.000283	0.003421

12	3	–0.000143	–0.000113	0.000183
12	4	0.000933	–0.000096	0.000938
12	5	–0.000017	–0.000261	0.000261
12	6	–0.000252	0.000189	0.000315
12	7	–0.000016	0.000279	0.000279
12	8	–0.000102	0.000003	0.000102
12	9	–0.000013	0.000043	0.000045
12	10	–0.000127	0.000071	0.000145
12	11	0.000088	0.000115	0.000144
12	12	0.000543	–0.000040	0.000544
14	0	–0.000029	0.000000	0.000029
14	1	0.000001	0.000001	0.000002
14	2	0.000015	0.000000	0.000015
14	3	0.000003	–0.000001	0.000003
14	4	0.000002	0.000002	0.000003
14	5	0.000000	0.000002	0.000002
14	6	0.000005	0.000000	0.000005
14	7	–0.000001	0.000001	0.000001
14	8	–0.000002	0.000000	0.000002
14	9	0.000000	0.000000	0.000000
14	10	0.000001	0.000000	0.000001
14	11	0.000000	–0.000001	0.000001
14	12	–0.000001	0.000000	0.000001
14	13	0.000000	0.000000	0.000000
14	14	0.000001	0.000000	0.000001

[a] The CF parameters are only listed for non-negative values of q. The values with negative q are given by $B_{k-q} = (-1)^q B_{kq}{}^*$.

Table S16. Squared magnitudes of projections of the *ab initio* CF eigenstates calculated for **1** onto angular momentum eigenstates with angular momentum J = 15/2 and projection M.

M	KD1		KD2		KD3		KD4		KD5		KD6		KD7		KD8	
−15/2	0.109	0.846	0.000	0.001	0.006	0.022	0.001	0.013	0.000	0.001	0.000	0.001	0.000	0.000	0.000	0.000
−13/2	0.000	0.000	0.002	0.843	0.003	0.063	0.011	0.025	0.043	0.001	0.007	0.000	0.001	0.001	0.000	0.000
−11/2	0.005	0.038	0.000	0.009	0.086	0.344	0.033	0.343	0.008	0.061	0.002	0.057	0.006	0.007	0.001	0.001
−9/2	0.000	0.000	0.000	0.116	0.002	0.043	0.041	0.106	0.432	0.011	0.166	0.005	0.038	0.030	0.008	0.003
−7/2	0.000	0.002	0.000	0.005	0.046	0.167	0.002	0.000	0.039	0.085	0.020	0.369	0.097	0.118	0.012	0.038
−5/2	0.000	0.000	0.000	0.016	0.000	0.064	0.055	0.140	0.025	0.005	0.137	0.027	0.216	0.161	0.114	0.040
−3/2	0.000	0.000	0.001	0.003	0.038	0.053	0.037	0.047	0.074	0.130	0.002	0.011	0.110	0.172	0.087	0.235
−1/2	0.000	0.000	0.001	0.004	0.012	0.051	0.057	0.086	0.042	0.045	0.166	0.029	0.028	0.016	0.336	0.127
1/2	0.000	0.000	0.004	0.001	0.051	0.012	0.086	0.057	0.045	0.042	0.029	0.166	0.016	0.028	0.127	0.336
3/2	0.000	0.000	0.003	0.001	0.053	0.038	0.047	0.037	0.130	0.074	0.011	0.002	0.172	0.110	0.235	0.087
5/2	0.000	0.000	0.016	0.000	0.064	0.000	0.140	0.055	0.005	0.025	0.027	0.137	0.161	0.216	0.040	0.114
7/2	0.002	0.000	0.005	0.000	0.167	0.046	0.000	0.002	0.085	0.039	0.369	0.020	0.118	0.097	0.038	0.012
9/2	0.000	0.000	0.116	0.000	0.043	0.002	0.106	0.041	0.011	0.432	0.005	0.166	0.030	0.038	0.003	0.008
11/2	0.038	0.005	0.009	0.000	0.344	0.086	0.343	0.033	0.061	0.008	0.057	0.002	0.007	0.006	0.001	0.001
13/2	0.000	0.000	0.843	0.002	0.063	0.003	0.025	0.011	0.001	0.043	0.000	0.007	0.001	0.001	0.000	0.000
15/2	0.846	0.109	0.001	0.000	0.022	0.006	0.013	0.001	0.001	0.000	0.001	0.000	0.000	0.000	0.000	0.000

Table S17. Squared magnitudes of projections of the *ab initio* CF eigenstates calculated for **2** onto angular momentum eigenstates with angular momentum J = 15/2 and projection M.

M	**KD1**		**KD2**		**KD3**		**KD4**		**KD5**		**KD6**		**KD7**		**KD8**	
−15/2	0.708	0.250	0.000	0.000	0.000	0.042	0.000	0.000	0.000	0.000	0.000	0.000	0.000	0.000	0.000	0.000
−13/2	0.000	0.000	0.879	0.063	0.000	0.000	0.051	0.007	0.000	0.000	0.000	0.000	0.000	0.000	0.000	0.000
−11/2	0.030	0.010	0.000	0.000	0.004	0.896	0.001	0.000	0.001	0.056	0.001	0.001	0.000	0.001	0.000	0.000
−9/2	0.000	0.000	0.050	0.004	0.000	0.002	0.763	0.098	0.003	0.005	0.043	0.010	0.013	0.005	0.004	0.000
−7/2	0.001	0.000	0.000	0.000	0.000	0.053	0.003	0.001	0.008	0.742	0.020	0.035	0.027	0.078	0.001	0.030
−5/2	0.000	0.000	0.003	0.000	0.000	0.000	0.063	0.008	0.008	0.005	0.375	0.106	0.213	0.090	0.127	0.001
−3/2	0.000	0.000	0.000	0.000	0.000	0.003	0.000	0.001	0.002	0.153	0.008	0.051	0.104	0.351	0.003	0.321
−1/2	0.000	0.000	0.000	0.000	0.000	0.000	0.003	0.001	0.016	0.003	0.278	0.072	0.094	0.023	0.508	0.004
1/2	0.000	0.000	0.000	0.000	0.000	0.000	0.001	0.003	0.003	0.016	0.072	0.278	0.023	0.094	0.004	0.508
3/2	0.000	0.000	0.000	0.000	0.003	0.000	0.001	0.000	0.153	0.002	0.051	0.008	0.351	0.104	0.321	0.003
5/2	0.000	0.000	0.000	0.003	0.000	0.000	0.008	0.063	0.005	0.008	0.106	0.375	0.090	0.213	0.001	0.127
7/2	0.000	0.001	0.000	0.000	0.053	0.000	0.001	0.003	0.742	0.008	0.035	0.020	0.078	0.027	0.030	0.001
9/2	0.000	0.000	0.004	0.050	0.002	0.000	0.098	0.763	0.005	0.003	0.010	0.043	0.005	0.013	0.000	0.004
11/2	0.010	0.030	0.000	0.000	0.896	0.004	0.000	0.001	0.056	0.001	0.001	0.001	0.001	0.000	0.000	0.000
13/2	0.000	0.000	0.063	0.879	0.000	0.000	0.007	0.051	0.000	0.000	0.000	0.000	0.000	0.000	0.000	0.000
15/2	0.250	0.708	0.000	0.000	0.042	0.000	0.000	0.000	0.000	0.000	0.000	0.000	0.000	0.000	0.000	0.000

Table S18. Squared magnitudes of projections of the *ab initio* CF eigenstates calculated for **3** onto angular momentum eigenstates with angular momentum J = 15/2 and projection M.

M	**KD1**		**KD2**		**KD3**		**KD4**		**KD5**		**KD6**		**KD7**		**KD8**	
−15/2	0.941	0.023	0.000	0.000	0.034	0.000	0.001	0.000	0.000	0.000	0.000	0.000	0.000	0.000	0.000	0.000
−13/2	0.000	0.000	0.751	0.210	0.001	0.000	0.037	0.000	0.001	0.000	0.000	0.000	0.000	0.000	0.000	0.000
−11/2	0.033	0.001	0.002	0.001	0.927	0.001	0.006	0.000	0.028	0.001	0.000	0.000	0.000	0.000	0.000	0.000
−9/2	0.000	0.000	0.027	0.008	0.012	0.000	0.910	0.000	0.009	0.000	0.005	0.024	0.000	0.003	0.000	0.001
−7/2	0.001	0.000	0.000	0.000	0.023	0.000	0.015	0.000	0.831	0.031	0.006	0.024	0.038	0.016	0.014	0.001
−5/2	0.000	0.000	0.001	0.000	0.001	0.000	0.026	0.000	0.011	0.003	0.108	0.532	0.057	0.166	0.011	0.083
−3/2	0.000	0.000	0.000	0.000	0.002	0.000	0.003	0.000	0.072	0.001	0.049	0.001	0.384	0.173	0.287	0.028
−1/2	0.000	0.000	0.000	0.000	0.000	0.000	0.001	0.000	0.006	0.006	0.023	0.227	0.051	0.111	0.052	0.523
1/2	0.000	0.000	0.000	0.000	0.000	0.000	0.000	0.001	0.006	0.006	0.227	0.023	0.111	0.051	0.523	0.052
3/2	0.000	0.000	0.000	0.000	0.000	0.002	0.000	0.003	0.001	0.072	0.001	0.049	0.173	0.384	0.028	0.287
5/2	0.000	0.000	0.000	0.001	0.000	0.001	0.000	0.026	0.003	0.011	0.532	0.108	0.166	0.057	0.083	0.011
7/2	0.000	0.001	0.000	0.000	0.000	0.023	0.000	0.015	0.031	0.831	0.024	0.006	0.016	0.038	0.001	0.014
9/2	0.000	0.000	0.008	0.027	0.000	0.012	0.000	0.910	0.000	0.009	0.024	0.005	0.003	0.000	0.001	0.000
11/2	0.001	0.033	0.001	0.002	0.001	0.927	0.000	0.006	0.001	0.028	0.000	0.000	0.000	0.000	0.000	0.000
13/2	0.000	0.000	0.210	0.751	0.000	0.001	0.000	0.037	0.000	0.001	0.000	0.000	0.000	0.000	0.000	0.000
15/2	0.023	0.941	0.000	0.000	0.000	0.034	0.000	0.001	0.000	0.000	0.000	0.000	0.000	0.000	0.000	0.000

Table S19. Magnitudes of transition magnetic moment matrix elements (in Bohr magneton) calculated for **1**.

Inital KD	Final KD	Climbing transition	Crossing transition
1	1	3.270723	0.004067
1	2	1.816594	0.015690
1	3	0.439783	0.044220
1	4	0.112066	0.028040
1	5	0.069273	0.031111
1	6	0.031182	0.052896
1	7	0.024782	0.019968
1	8	0.006085	0.006893
2	2	3.112271	0.103044
2	3	2.513508	0.235163
2	4	1.142419	0.203697
2	5	0.146728	0.155801
2	6	0.061691	0.047503
2	7	0.054300	0.050329
2	8	0.017630	0.022692
3	3	3.117397	0.680722
3	4	3.108069	0.779783
3	5	0.820663	0.326853
3	6	0.117437	0.190814
3	7	0.063101	0.020960
3	8	0.027766	0.028347
4	4	2.001637	1.338568
4	5	3.034389	1.247653
4	6	0.364887	0.544258
4	7	0.129622	0.110907
4	8	0.018501	0.026233
5	5	0.907703	2.224675
5	6	2.384944	1.347259
5	7	0.178099	0.339365
5	8	0.083089	0.083618
6	6	0.336503	2.542494
6	7	1.696840	1.262215
6	8	0.090388	0.098003
7	7	0.598727	2.865092
7	8	1.082637	1.308627
8	8	3.328911	0.433699

Table S20. Magnitudes of transition magnetic moment matrix elements (in Bohr magneton) calculated for **2**.

Inital KD	Final KD	Climbing transition	Crossing transition
1	1	3.275365	0.000267
1	2	1.797116	0.000483
1	3	0.241095	0.000276
1	4	0.194111	0.000889
1	5	0.049208	0.007495
1	6	0.019410	0.008521
1	7	0.009726	0.008292
1	8	0.003668	0.002831
2	2	2.873634	0.003059
2	3	2.354716	0.003847
2	4	0.294039	0.001616
2	5	0.188009	0.005528
2	6	0.055897	0.040384
2	7	0.029922	0.032788
2	8	0.013183	0.011148
3	3	2.508224	0.021568
3	4	2.763925	0.023921
3	5	0.313509	0.057102
3	6	0.075179	0.015980
3	7	0.042545	0.049037
3	8	0.036371	0.028431
4	4	2.086771	0.155993
4	5	3.058253	0.135879
4	6	0.350908	0.357397
4	7	0.289936	0.222460
4	8	0.123805	0.092096
5	5	1.554916	0.849227
5	6	3.091049	0.414051
5	7	0.525459	1.048066
5	8	0.250642	0.221877
6	6	0.735296	2.729987
6	7	2.198322	1.044597
6	8	0.343576	0.305524
7	7	1.244040	2.703586
7	8	0.925055	1.429811
8	8	3.408605	0.280834

Table S21. Magnitudes of transition magnetic moment matrix elements (in Bohr magneton) calculated for **3**.

Inital KD	Final KD	Climbing transition	Crossing transition
1	1	3.282332	0.000202
1	2	1.784177	0.000376
1	3	0.278847	0.000301
1	4	0.246321	0.000789
1	5	0.073616	0.003553
1	6	0.011179	0.004669
1	7	0.004807	0.003969
1	8	0.001894	0.001925
2	2	2.976238	0.001981
2	3	2.345128	0.002679
2	4	0.315360	0.001868
2	5	0.234010	0.004523
2	6	0.096692	0.022154
2	7	0.025883	0.028724
2	8	0.012181	0.007446
3	3	2.609040	0.013461
3	4	2.781676	0.013885
3	5	0.236505	0.021645
3	6	0.069991	0.039196
3	7	0.081107	0.041371
3	8	0.046125	0.008263
4	4	2.092984	0.080474
4	5	3.097611	0.077712
4	6	0.329325	0.212422
4	7	0.235932	0.163263
4	8	0.141990	0.070857
5	5	1.654753	0.434816
5	6	3.193685	0.418440
5	7	0.414633	0.954147
5	8	0.335904	0.255383
6	6	0.980518	2.144649
6	7	2.707761	1.082968
6	8	0.446506	0.411842
7	7	1.460122	2.558953
7	8	0.946497	1.488574
8	8	3.279057	0.654074

References

1. J. P. Durrant, J. Tang, A. Mansikkamäki, R. A. Layfield, *Chem. Commun.* **2020**, *4*, 4.
2. K. E. du Plooy, J. du Toit, D. C. Levendis, N. J. Coville, *J. Organometalic Chem.* **1996**, *508*, 231.
3. F. G. N. Cloke, M. C. Kuchta, R. M. Harker, P. B. Hitchcock, J. S. Parry, *Organometallics* **2000**, *19*, 5795.
4. O. V. Dolomanov, L. J. Bourhis, R. J. Gildea, J. A. K. Howard, H. Puschmann, *J. Appl. Cryst.*, **2009**, *42*, 339.
5. G. M. Sheldrick, *Acta Cryst.*, **2015**, *A71*, 3.
6. G. M. Sheldrick, *Acta Cryst.*, **2015**, *C71*, 3.
7. D. Gatteschi, R. Sessoli, J. Villain, *Molecular Nanomagnets* (Oxford Univ. Press, 2006).
8. Y–N. Guo, G–F. Xu, Y. Guo, J. Tang, *Dalton Trans.*, **2011**, *40*, 9953.
9. a) ADF2019. SCM, Theoretical Chemistry, Vrije Universiteit Amsterdam, The Neatherlands. http://www.scm.com. 2019; b) G. te Velde, F. M. Bickelhaupt, E. J. Baerendes, C. Fonseca Guerra, S. J. A. Gisbergen, J. G. Snijders, T. Ziegler. *J. Comp. Chem*. **2001**, *22*, 931–967; c) C. Fonseca Guerra, J. G. Snijders, G. te Velde, E. J. Baerends. *Theor. Chem. Acc*. **1998**, *99*, 391–403.
10. a) J. P. Perdew, K. Burke, M. Ernzerhof. *Phys. Rev. Lett*., **1996**, *77*, 3865–3868; b) J. P. Perdew, K. Burke, M. Ernzerhof. *Phys. Rev. Lett*., **1996**, *78*, 1396.
11. S. Grimme, J. Antony, S. Ehrlich, H. Krieg. *J. Chem. Phys*. **2010**, *132*, 154104.
12. S. Grimme, S. Ehrlich, L. Goerigk. *J. Comp. Chem*. **2011**, *32*, 1456–1465.
13. a) E. van Lenthe, E. J. Baerends, J. G. Snijders. *J. Chem. Phys*. **1993**, *99*, 4597–4610; b) E. van Lenthe, E. J. Baerends, J. G. Snijders. *J. Chem. Phys*. **1994**, *101*, 9783–9792; c) E. van Lenthe, R. van Leeuwen, E. J. Baerends, J. G. Snijders. *Int. J. Quantum. Chem*. **1996**, *57*, 281–293.
14. E. van Lenthe, E. J. Baerends. *J. Comp. Chem*. **2003**, *24*, 1142–1156.
15. I. F. Galván, M. Vacher, A. Alavi, C. Angeli, F. Aquilante, J. Autschbach, J. J. Bao, S. I. Bokarev, N. A. Bogdanov, R. K. Carlson, L. F. Chibotaru, J. Creutzberg, N. Dattani, M. G. Delcey, S. S. Dong, A. Dreuw, L. Freitag, L. M. Frutos, L. Gagliardi, F. Gendron, A. Giussani, L. González, G. Grell, M. Guo, C. E. Hoyer, M. Johansson, S. Keller, S. Knecht, G. Kovačević, E. Källman, G. L. Manni, M. Lundberg, Y. Ma, S. Mai, J. P. Malhado, P. Å. Malmqvist, P. Marquetand, S. A. Mewes, J. Norell, M. Olivucci, M. Oppel, Q. M. Phung, K. Pierloot, F. Plasser, M. Reiher, A. M. Sand, I. Schapiro, P. Sharma, C. J. Stein, L. K. Sørensen, D. G. Truhlar, M. Ugandi, L. Ungur, A. Valentini, S. Vancoillie, V. Veryazov, O. Weser, T. A. Wesołowski, P.-O. Widmark, S. Wouters, A. Zech, J. P. Zobel, R. Lindh. J. Chem. Theory Comput., **2019**, *15*, 5925–5964.
16. a) B. O. Roos in Advances in Chemical Physics, Ab Initio Methods in Quantum Chemistry II, Vol. 69 (Ed.: K. P. Lawley), Wiley, New York, 1987, pp. 399–455; b) P. Siegbahn, A. Heiberg, B. Roos, B. Levy. *Phys. Scripta*, **1980**, *21*, 323–327; c) B. O. Roos, P. R. Taylor, P. E. M. Siegbahn. *Chem. Phys*., **1980**, *48*, 157–173; d) P. E. M. Siegbahn, J. Almlöf, A. Heiberg, B. Roos. *J. Chem. Phys*., **1981**, *74*, 2384–2396; e) B. O. Roos, R. Lindh, P. Å. Malmqvist, V. Veryazov, P.-O. Widmark. Multiconfigurational Quantum Chemistry. Wiley, Hoboken, NJ, 2016.
17. P. Å. Malmqvist, B. O. Roos, B. Schimmelpfenning. *Chem. Phys. Lett*., **2002**, *357*, 230–240.
18. a) B. A. Heß, C. M. Marian, U. Wahlgren, O. Gropen. *Chem. Phys. Lett*., **1996**, *251*, 365–371; b) O. Christiansen, J. Gauss, B. Schimmelpfennig. *Phys. Chem. Chem. Phys*., **2000**, *2*, 965–971.
19. a) L. F. Chibotaru, L. Ungur. *J. Chem. Phys*., **2012**, *137*, 064112; b) L. Ungur, L. F. Chibotaru. Computational Modelling of Magnetic Properties of Lanthanide Compounds in Lanthanide and Actinides in Molecular Magnetism. Eds. R. A. Layfield, M. Murugesu. Wiley-VCH, Weinheim, Germany, 2015; c) L. Ungur, L. F. Chibotaru. *Chem. Eur. J*. **2017**, *23*, 3708–3718.
20. a) W. Kutzelnigg, W. Liu. *J. Chem. Phys*., **2005**, *123*, 241102; b) M. Filatov. *J. Chem. Phys*. **2006**, *125*, 107101; c) D. Peng, M. Reiher. *Theor. Chem. Acc*., **2012**, *131*, 1.
21. a) P.-O. Widmark, P.-Å. Malmqvist, B. O. Roos. *Theor. Chim. Acta*, **1990**, *77*, 1432–2234; b) B. Roos, V. Veryazov, P.-O. Widmark. *Theor. Chem. Acc*. **2004**, *111*, 345–351; c) B. O. Roos, R. Lindh, P.-Å. Malmqvist, V. Veryazov, P.-O. Widmark. *J. Phys. Chem. A*, **2004**, *108*, 2851–2858; d) B. O. Roos, R. Lindh, P.-Å. Malmqvist, V. Veryazov, P.-O. Widmark, A. C. Borin. *J. Phys. Chem. A*. **2008**, *112*, 11431–11435.
22. a) N. Iwahara, L. F. Chibotaru. *Phys. Rev. B*. **2015**, 91, 174438; b) N. Iwahara, L. Ungur, L. F. Chibotaru. *Phys. Rev. B.*, **2018**, *98*, 054436.
